# Hydrogen-Bond-Assisted Hydroxylation of *o*-Fluorobenzamides: A Transition-Metal-Free Approach to Salicylamides

**DOI:** 10.3390/molecules31111887

**Published:** 2026-06-01

**Authors:** Ting Chen, Huiwen Lei, Ting Huang, Hanjun Chen, Shuo Li, Fuqiang Liu, Hai-Chao Xu, Jinhai Shen

**Affiliations:** 1 College of Environment and Public Health, Xiamen Huaxia University, Xiamen 361024, China; chent@hxxy.edu.cn (T.C.); leihwen@163.com (H.L.); 15207041950@163.com (T.H.); chenhanj5@126.com (H.C.); 15943889975@163.com (S.L.); 2 College of Chemistry, Fuzhou University, Fuzhou 350108, China; lfqsmiling@126.com; 3 College of Chemistry and Chemical Engineering, Xiamen University, Xiamen 361005, China; 4 Fujian Key Laboratory of Molecular Medicine (Huaqiao University), Xiamen 361021, China

**Keywords:** salicylamides, S_N_Ar, hydroxylation, alkoxylation, hydrogen bonding

## Abstract

A transition-metal-free method for the selective hydroxylation of *o*-fluorobenzamides has been developed, providing efficient access to salicylamides under basic conditions. The reaction proceeds with KO*t*-Bu and H_2_O in DMSO, affording the desired products in good to excellent yields with broad functional group tolerance and high *ortho* selectivity. Mechanistic studies indicate that the reaction critically depends on the hydrogen-bond donor ability and conformational flexibility of the amide. This work provides a practical approach to salicylamides and related derivatives and highlights hydrogen-bond-controlled reactivity in nucleophilic aromatic substitution.

## 1. Introduction

**Figure 1:**
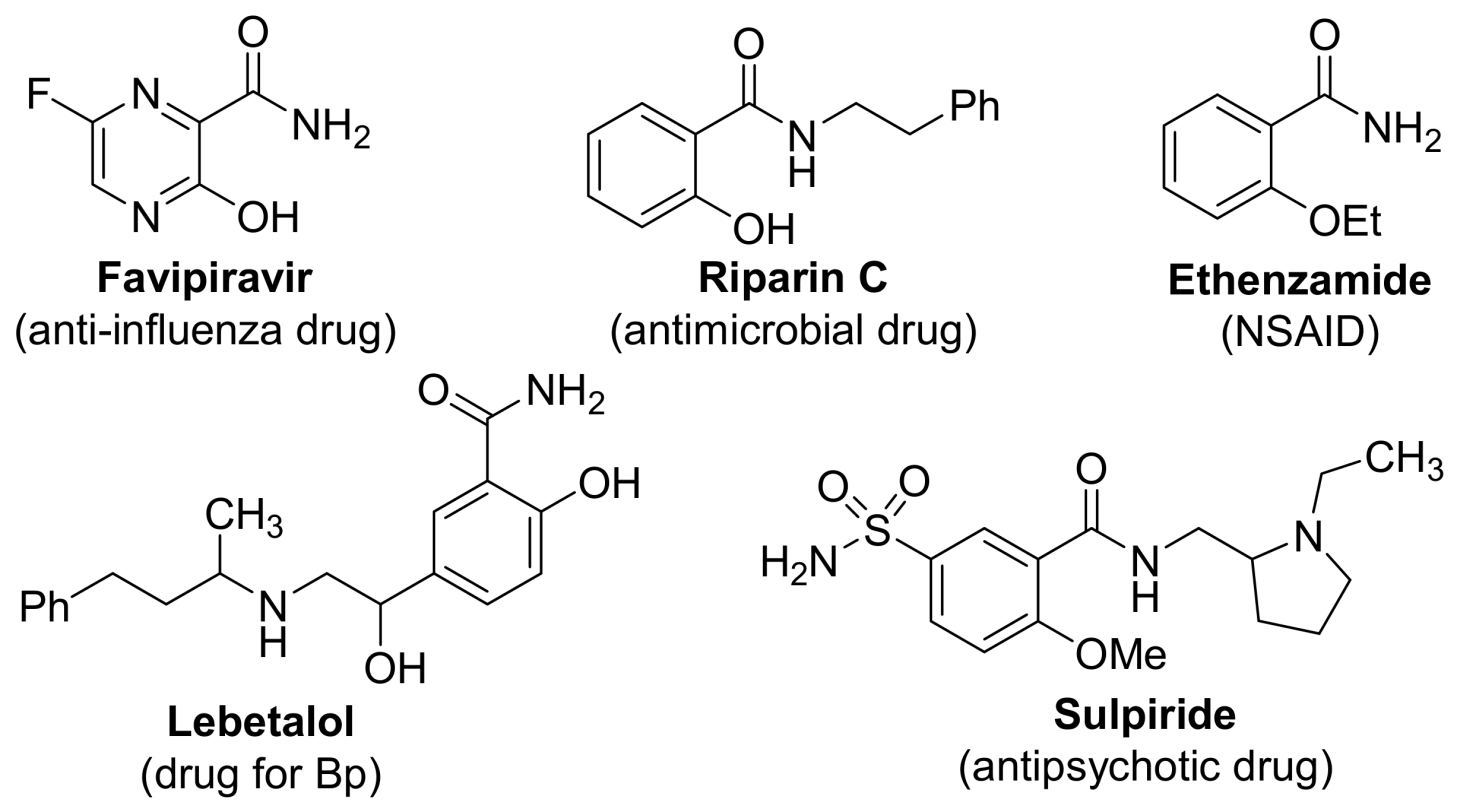
Selected pharmaceutically important drug molecules with the *ortho*-hydroxy benzamide backbone.

Salicylamide derivatives represent an important class of compounds widely found in pharmaceuticals [1], agrochemicals [2], and functional materials [3,4,5,6]. These structural motifs are present in numerous bioactive molecules and serve as key intermediates in the synthesis of nonsteroidal anti-inflammatory drugs (NSAIDs) and other medicinally relevant compounds (Figure 1) [7,8,9,10,11,12]. Accordingly, the development of efficient and selective methods for accessing salicylamides remains an important objective in synthetic chemistry.

**Scheme 1:**
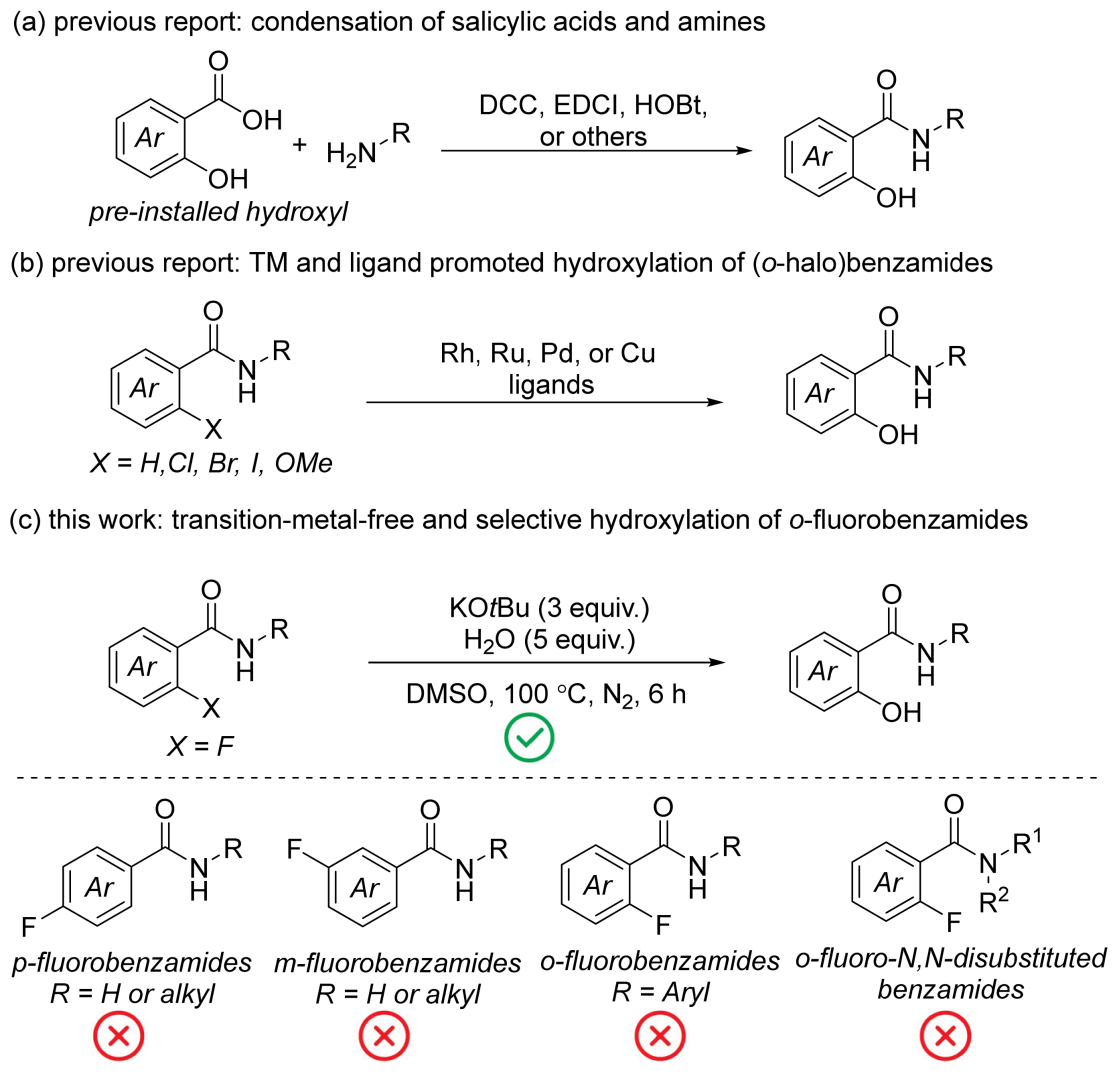
Synthesis of salicylamides.

Traditionally, salicylamides are prepared from salicylic acid derivatives through amide formation or via multi-step sequences involving prefunctionalized phenolic substrates (Scheme 1a) [13]. However, these approaches often require pre-installed hydroxyl groups or harsh conditions and exhibit a limited substrate scope. Alternatively, transition-metal-catalyzed hydroxylation of arenes and aryl halides has emerged as a powerful strategy for the direct construction of phenolic compounds (Scheme 1b) [14,15,16,17,18]. For example, copper-mediated ortho-hydroxylation of benzamides using directing groups has been reported, enabling regioselective functionalization of aromatic C–H or C–X bonds [19,20,21,22,23,24,25]. Despite these advances, such methods typically require transition-metal catalysts, specially designed directing groups, or additional ligands, which may limit their practicality and operational simplicity.

*o*-Halobenzamides are readily accessible and structurally versatile building blocks in organic synthesis, widely used in cross-coupling reactions and medicinal chemistry [26]. In principle, these substrates could provide a straightforward entry to salicylamides via nucleophilic aromatic substitution (S_N_Ar). However, conventional S_N_Ar reactions are primarily governed by electronic activation and typically require strongly electron-deficient substrates [27,28,29,30]. Simple benzamide substituents are often insufficient to promote efficient substitution under mild conditions, making the direct hydroxylation of *o*-halobenzamides without the assistance of transition metals highly challenging and underdeveloped.

In our previous studies on *o*-fluorobenzamides [31,32], we found that *N*-benzyl-substituted *o*-fluorobenzamides (tertiary amides) readily underwent base-promoted cascade reactions to afford 3-hydroxyisoindolinones and 2-(2-aminobenzoyl)benzoic acids. Interestingly, when the corresponding secondary amide substrates bearing an N–H bond were employed under similar basic conditions, the reaction pathway changed dramatically, and only *ortho*-hydroxylation products (salicylamides) were obtained. This distinct structure-dependent reactivity suggested that the amide N–H functionality may play a critical role in controlling the reaction outcome.

Based on these observations and our continuing interest in the chemistry of amides, herein we report a simple and transition-metal-free hydroxylation of *o*-fluorobenzamides to afford salicylamides under basic conditions (Scheme 1). The reaction proceeds using KO*t*-Bu (3.0 equiv) and H_2_O (5.0 equiv) at 100 °C under a nitrogen atmosphere for 6 h, providing the corresponding *o*-hydroxybenzamides in good yields. Notably, the reaction exhibits a pronounced dependence on both substitution pattern and amide N–H functionality: *o*-fluorobenzamides bearing an N–H bond undergo efficient hydroxylation, whereas *para*-substituted analogs, N-aryl, and N,N-disubstituted derivatives remain unreactive under identical conditions.

Compared with existing methods, this protocol features several advantages, including the absence of transition-metal catalysts, the use of simple and inexpensive reagents, and operational simplicity. More importantly, the distinct structure–reactivity relationship observed in this system suggests that factors beyond classical electronic activation play a decisive role in controlling reactivity. As illustrated in Scheme 1, the requirement for an *ortho*-amide N–H group indicates a hydrogen-bond-assisted activation mode, in which conformational preorganization enables efficient nucleophile delivery. This hydrogen-bond-controlled S_N_Ar reactivity provides a new perspective for overcoming the intrinsic limitations of electronically driven aromatic substitution.

## 2. Results and Discussion

The reaction conditions were systematically optimized using *N*-benzyl *o*-fluorobenzamide **1a** as the model substrate under a nitrogen atmosphere (Table 1). Initially, various bases were evaluated in DMSO at 120 °C for 6 h. Strong inorganic bases such as KOH and NaOH afforded good yields (80–84%, entries 1–2), while weaker bases including K_2_CO_3_ and Cs_2_CO_3_ resulted in significantly decreased reactivity (entries 3–4). Organic bases such as DBU and Et_3_N were completely ineffective (entries 7–8). Notably, KO*t*-Bu provided the highest yield (85%, entry 5) and was therefore selected for further studies. Subsequently, the loading of KO*t*-Bu was investigated. Increasing the amount from 1.0 to 3.0 equivalents led to a gradual improvement in yield (68% to 85%, entries 5, 9–10), while a further increase to 4.0 equivalents showed no additional benefit (entry 11). The solvent effect was then examined. Among the solvents tested, DMSO proved to be optimal, delivering the highest yield (85%). Other polar aprotic solvents such as DMF and NMP afforded only moderate yields, whereas non-polar solvents (e.g., toluene and 1,4-dioxane) completely suppressed the reaction (entries 12–15). Notably, protic solvents such as *tert*-butanol (*t*-BuOH) were also ineffective, and no desired product was observed under these conditions (entry 16). Further optimization of temperature revealed that 100 °C was optimal, affording the desired product in 87% yield. Both lower and higher temperatures led to diminished or comparable yields (entries 17–20). Notably, the reaction could still proceed under an air atmosphere, affording the desired product in approximately 68% yield (entry 21). Repeated experiments under non-inert conditions provided comparable results, suggesting reasonable reproducibility and a certain degree of tolerance toward air and moisture, although slightly lower yields were observed compared with those obtained under a nitrogen atmosphere. In the absence of water, only a trace amount of the desired product was observed (entry 22). The optimized reaction conditions were identified as follows: 3 equiv of KO*t*-Bu as the additive and DMSO as the solvent at 100 °C for 6 h under a nitrogen atmosphere (entry 18).

**Scheme 2:**
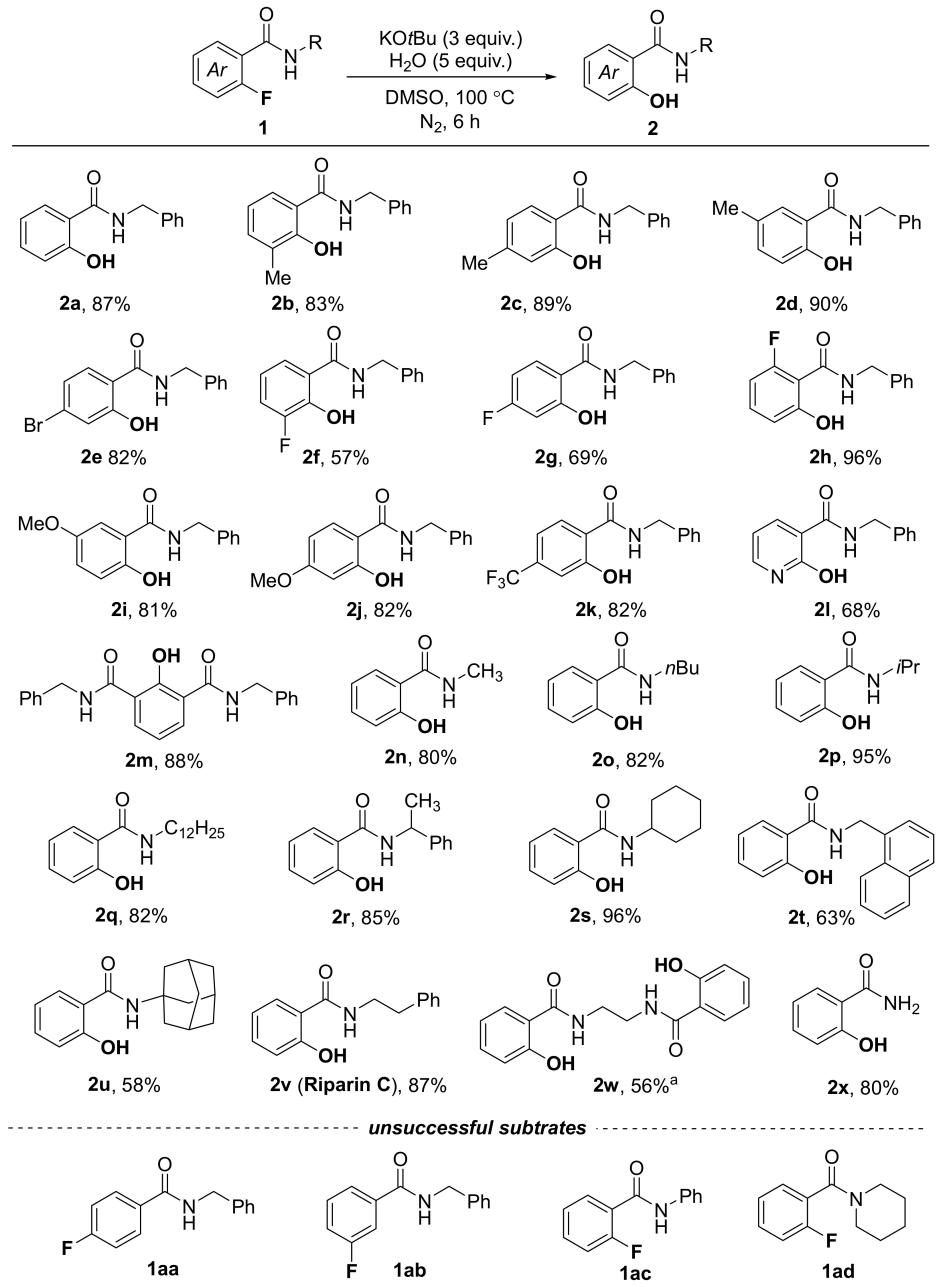
Substrate scope for the transition-metal-free hydroxylation of *o*-fluorobenzamides. ^a^ 6 equiv of KO*t*-Bu was used.

With the optimized conditions in hand, we next explored the substrate scope of this transformation (Scheme 2). We first investigated the functional group tolerance on the aromatic ring of *o*-fluorobenzamides. In terms of steric effects, substrates bearing methyl substituents at the 3-, 4-, or 5-position all afforded the corresponding salicylamides in good yields (83–89%). Other halogen substituents, such as fluorine and bromine, were well tolerated, delivering the corresponding halogenated salicylamides. Notably, in the case of difluorinated substrates (2,3-, 2,4-, and 2,6-difluorobenzamides, **2f**–**2h**), nucleophilic substitution occurred exclusively at the ortho position relative to the amide group, while the meta- and para-fluorine substituents remained intact. This result highlights the crucial role of the amide-directed ortho effect in governing site selectivity.

Both electron-donating and electron-withdrawing substituents, such as methoxy and trifluoromethyl groups, were also compatible, affording the desired products in good yields (81–83%, **2i**–**2k**). In addition, heteroaromatic substrates could be employed; for example, 2-fluoronicotinamide underwent smooth transformation to give the corresponding hydroxylated product in 68% yield (**2l**). Furthermore, the reaction enabled the synthesis of 2-hydroxyisophthalamide (**2m**) in 88% yield.

We then examined the scope with respect to substitution on the amide nitrogen. A wide range of N-alkyl substituents, including methyl, *n*-butyl, isopropyl, *n*-dodecyl, and α-phenethyl groups, were well tolerated, affording the corresponding salicylamides in good to excellent yields (**2n**–**2r**, 80–95%). Bulky substituents such as cyclohexyl, 1-naphthylmethyl, and adamantyl groups were also compatible. Notably, this protocol enabled the efficient synthesis of the bioactive molecule Riparin C (**2v**) in 87% yield. In addition, the reaction proceeded smoothly with the unsubstituted amide (–CONH_2_), affording product **2x** in 80% yield.

In contrast, *p*-fluorobenzamide **1aa** and *m*-fluorobenzamide **1ab** failed to undergo the reaction. Moreover, N-aryl-substituted *o*-fluorobenzamide **1ac** and *N*,*N*-disubstituted analog **1ad** were also unreactive under the standard conditions. These observations indicate that both the hydrogen-bond donor ability and the conformational flexibility of the amide group are critical for reactivity. In N-aryl amides, conjugation between the nitrogen lone pair and the aromatic substituent reduces the effective hydrogen-bonding capability of the N–H unit and enforces a more planar geometry. As a result, the formation of a favorable six-membered, hydrogen-bond-assisted transition state is likely hindered, leading to suppressed reactivity. These findings further support a mechanism in which efficient nucleophile delivery relies on a properly oriented and sufficiently strong N–H···nucleophile interaction, rather than merely the presence of an amide functionality.

**Scheme 3:**
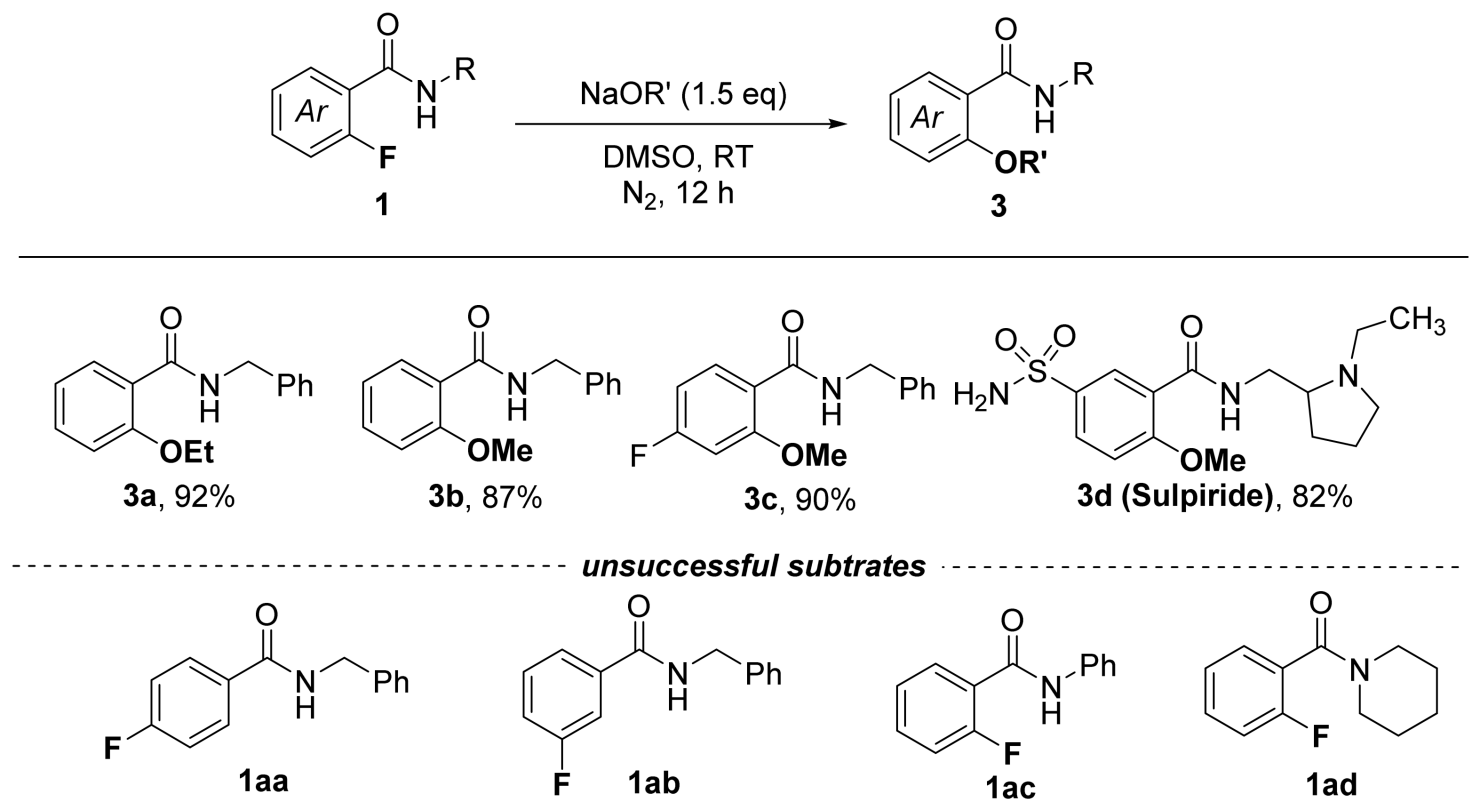
Alkoxylation of *o*-fluorobenzamides with sodium alkoxides.

**Scheme 4:**
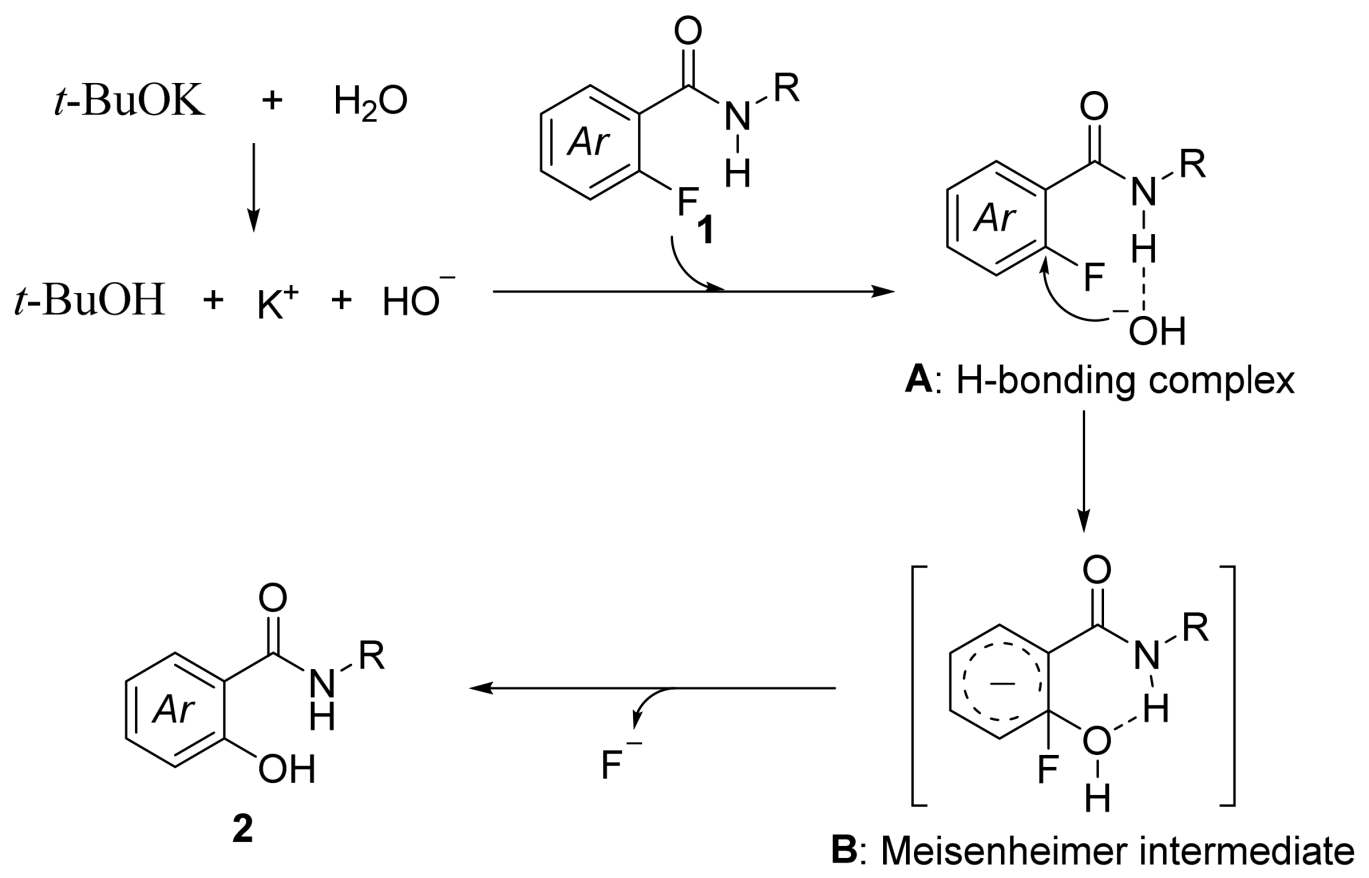
Proposed reaction mechanism.

In addition to hydroxylation, the reaction could be extended to more nucleophilic alkoxide species (Scheme 3). Under mild conditions, *o*-fluorobenzamides reacted readily with sodium alkoxides at room temperature to afford the corresponding *o*-alkoxybenzamides. This strategy enabled the direct synthesis of pharmaceutically relevant molecules such as sulpiride (**3d**). Consistent with the hydroxylation results, *p*- and *m*-fluorobenzamides, as well as *N*-aryl and *N*,*N*-disubstituted *o*-fluorobenzamides, remained unreactive under these conditions, further supporting the proposed hydrogen-bond-assisted S_N_Ar mechanism (Scheme 4).

Based on the experimental observations and previous reports [33,34], a plausible mechanism for the hydroxylation of *o*-fluorobenzamides is proposed (Scheme 4). The reaction is initiated by the generation of hydroxide under basic conditions, which remains highly nucleophilic in the polar aprotic solvent (DMSO). A key feature of this transformation is the formation of a hydrogen-bonded complex **A** between the amide N–H and the hydroxide ion, enabling preorganization of the nucleophile through a six-membered arrangement. This interaction facilitates intramolecular delivery of the nucleophile to the ipso carbon, leading to the formation of a Meisenheimer σ-complex **B**. The ortho-amide group not only activates the aromatic ring electronically but also stabilizes the intermediate through hydrogen bonding and inductive effects. Subsequent elimination of fluoride restores aromaticity and furnishes the corresponding salicylamide **2**. Although hydroxide is likely the active nucleophilic species, KO*t*-Bu provided superior reproducibility and broader substrate compatibility in the DMSO/H_2_O system.

The requirement for an N–H group, together with the pronounced solvent dependence, supports a hydrogen-bond-assisted S_N_Ar mechanism. Substrates lacking an effective hydrogen-bond donor or conformational flexibility [35] fail to undergo the reaction, highlighting the cooperative role of hydrogen bonding and structural preorganization in lowering the activation barrier.

**Scheme 5:**
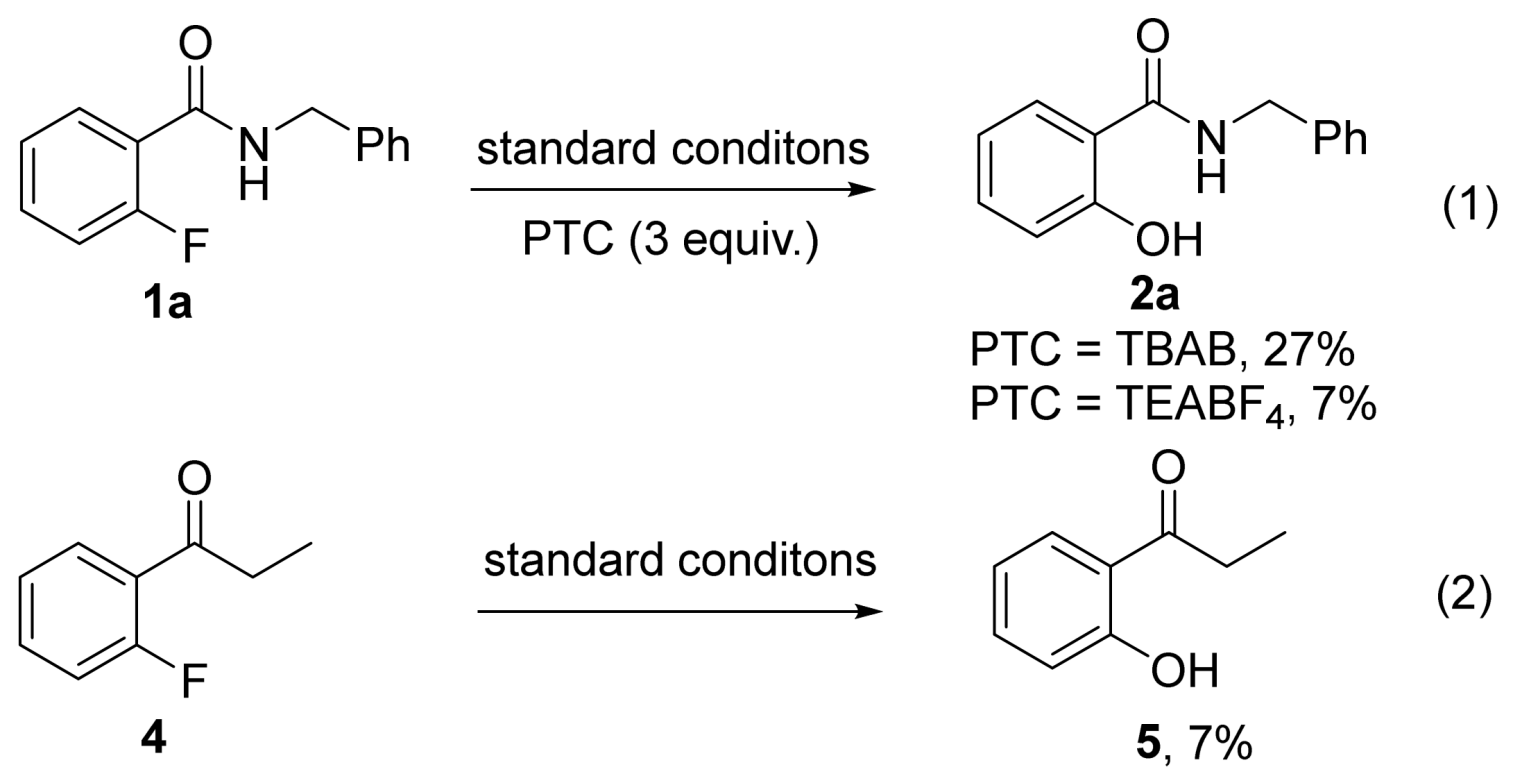
Mechanistic studies.

To further probe the proposed hydrogen-bond-assisted mechanism, additional control experiments were conducted. The addition of phase-transfer catalysts, such as tetrabutylammonium bromide (TBAB) or tetraethylammonium tetrafluoroborate (TEABF_4_), resulted in a significant decrease in yield (Scheme 5, Equation (1)). This effect is attributed to the formation of tight ion pairs between the ammonium cation and hydroxide, which reduces the availability of free, highly nucleophilic hydroxide. Moreover, such ion pairing is expected to interfere with the key N–H···hydroxide interaction required for nucleophile preorganization, thereby suppressing the reaction.

In a separate experiment, 1-(2-fluorophenyl)propan-1-one **4** showed only trace reactivity under the standard conditions (Scheme 5, Equation (2)). The starting material was largely decomposed, and only a 7% yield of the expected hydroxylated product **5** was detected. This result suggests that the amide N–H functionality may play an important role in facilitating the transformation, consistent with the proposed hydrogen-bond-assisted S_N_Ar pathway.

These results provide further evidence that both the availability of a free hydroxide nucleophile and a properly oriented, accessible N–H hydrogen-bond donor are essential for the reaction, supporting a cooperative hydrogen-bond-assisted S_N_Ar mechanism.

## 3. Experimental Sections

### 3.1. General Information

Unless otherwise stated, all commercial materials and solvents were used directly without further purification. ^1^H NMR spectra were recorded on 400 MHz spectrometers, and ^13^C NMR spectra were recorded on a 100 MHz spectrometer. Chemical shifts (in ppm) were referenced to tetramethylsilane (δ = 0 ppm) in CDCl_3_ as an internal standard at room temperature. ^13^C NMR spectra were obtained by using the same NMR spectrometers and were calibrated with CDCl_3_ (δ = 77.00 ppm). High-resolution mass spectra (HRMS) were equipped with an ESI source and a TOF detector. Column chromatography was performed on silica gel (200–300 mesh ASTM) using the reported eluents. Thin-layer chromatography (TLC) was carried out on 4 × 15 cm plates with a layer thickness of 0.2 mm (silica gel 60 F_254_). The 2-fluorobenzamide substrates **1n** and **1x** were obtained from commercial sources and used directly, while the remaining substrates were prepared according to the literature [31], as follows:


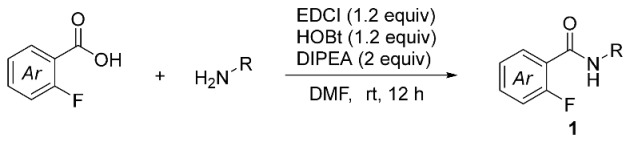


Amine (10 mmol), 2-fluorobenzoic acid (10 mmol), EDCI (1-Ethyl-3-(3-dimethylaminopropyl)carbodiimide, 12 mmol), HOBt (12 mmol), and DIPEA (20 mmol) were dissolved in 15 mL of anhydrous DMF. The mixture was stirred at room temperature for 12 h. Water was then added, and the mixture was extracted with EtOAc. The combined organic layers were washed with H_2_O and brine, dried over anhydrous Na_2_SO_4_, filtered, and concentrated under reduced pressure. The residue was purified by silica gel flash chromatography (EtOAc/PE = 1/10) to afford the desired product. All reagents were obtained from commercial sources and used without further purification.

### 3.2. General Procedure for the Synthesis of Salicylamides ***2***

A 10-mL oven-dried Schlenk tube equipped with a magnetic stir bar was charged with 2-fluorobenzamide **1** (0.3 mmol, 1.0 equiv) and potassium *tert*-butoxide (0.9 mmol, 3.0 equiv). The tube was evacuated and backfilled with nitrogen (three cycles). Anhydrous DMSO (1 mL) was added via syringe, followed by distilled water (27 μL, 1.5 mmol, 5 equiv). The mixture was heated in a preheated oil bath at 100 °C for 6 h under a positive nitrogen atmosphere. After cooling to room temperature, the reaction was quenched by the addition of water (10 mL). The aqueous layer was extracted with ethyl acetate (3 × 10 mL). The combined organic extracts were washed with brine (10 mL), dried over anhydrous Na_2_SO_4_, filtered, and concentrated under reduced pressure. The residue was purified by flash column chromatography on silica gel (200–30 mesh) using a gradient elution of ethyl acetate in petroleum ether (from 1:10 to 1:1, *v*/*v*) to afford the desired salicylamide **2**.


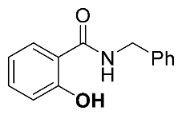


N-benzyl-2-hydroxybenzamide **2a**. 59.3 mg, 87% yield. White solid. R_f_ = 0.60 (EtOAc/PE = 1:4). ^1^H NMR (400 MHz, CDCl_3_) δ12.32 (s, 1H), 7.44–7.27 (m, 7H), 6.99 (d, *J* = 8.3 Hz, 1H), 6.82 (t, *J* = 7.6 Hz, 1H), 6.69 (s, 1H), 4.63 (d, *J* = 5.5 Hz, 2H); ^13^C NMR (100 MHz, CDCl_3_) δ 169.8, 161.6, 161.5, 137.4, 134.3, 128.9, 127.9, 125.4, 125.3, 118.7, 118.7, 118.6, 114.1, 43.7. The characterization data is in accordance with that reported in the literature [36].


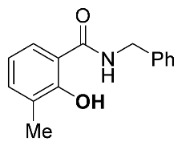


N-benzyl-2-hydroxy-3-methylbenzamide **2b**. 60.0 mg, 83% yield. White solid. R_f_ = 0.60 (EtOAc/PE = 1:4). ^1^H NMR (400 MHz, CDCl_3_) δ 12.54 (s, 1H), 7.45–7.23 (m, 7H), 7.19 (d, *J* = 8.0 Hz, 1H), 6.73 (t, *J* = 7.6 Hz, 1H), 6.57 (s, 1H), 4.63 (d, *J* = 5.6 Hz, 2H), 2.27 (s, 3H); ^13^C NMR (100 MHz, CDCl_3_) δ 170.2, 160.1, 137.5, 135.0, 128.9, 127.9, 127.8, 127.8, 122.7, 117.9, 113.2, 43.7, 15.8. The characterization data is in accordance with that reported in the literature [37].


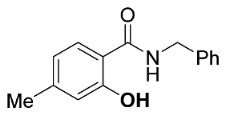


N-benzyl-2-hydroxy-4-methylbenzamide **2c**. White solid. 64.3 mg, 89% yield. R_f_ = 0.60 (EtOAc/PE = 1:4). ^1^H NMR (400 MHz, CDCl_3_) δ 12.29 (s, 1H), 7.43–7.29 (m, 5H), 7.22 (d, *J* = 8.1 Hz, 1H), 6.81 (s, 1H), 6.64 (d, *J* = 8.1 Hz, 1H), 6.52 (s, 1H), 4.63 (d, *J* = 5.5 Hz, 2H), 2.32 (s, 3H); ^13^C NMR (100 MHz, CDCl_3_) δ 169.8, 161.6, 145.4, 137.5, 128.9, 127.9, 127.8, 125.1, 119.8, 118.8, 111.4, 43.6, 21.6. The characterization data is in accordance with that reported in the literature [38].


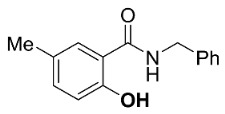


N-benzyl-2-hydroxy-5-methylbenzamide **2d**. White solid. 65.1 mg, 90% yield. R_f_ = 0.60 (EtOAc/PE = 1:4). ^1^H NMR (400 MHz, CDCl_3_) δ 12.10 (s, 1H), 7.48–7.31 (m, 5H), 7.23 (d, *J* = 8.4 Hz, 1H), 7.15 (s, 1H), 6.93 (d, *J* = 8.4 Hz, 1H), 6.59 (s, 1H), 4.66 (d, *J* = 5.4 Hz, 2H), 2.28 (s, 3H); ^13^C NMR (100 MHz, CDCl_3_) δ 169.8, 159.4, 137.5, 135.2, 128.9, 127.9, 127.9, 127.8, 125.2, 118.4, 113.7, 43.7, 20.5. The characterization data is in accordance with that reported in the literature [39].


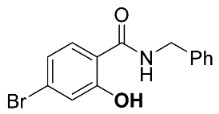


N-benzyl-4-bromo-2-hydroxybenzamide **2e**. White solid. 74.9 mg, 82% yield. R_f_ = 0.55 (EtOAc/PE = 1:4). ^1^H NMR (400 MHz, CDCl_3_) δ 12.47 (s, 1H), 7.44–7.30 (m, 6H), 7.19 (d, *J* = 8.3 Hz, 2H), 6.95 (d, *J* = 8.5 Hz, 1H), 6.54 (s, 1H), 4.62 (d, *J* = 5.5 Hz, 2H); ^13^C NMR (100 MHz, CDCl_3_) δ 169.2, 162.3, 137.1, 129.0, 128.2, 128.0, 128.0, 126.3, 122.0, 121.8, 113.0, 43.8. HRMS (ESI) *m*/*z* calcd for C_14_H_13_BrNO_2_ [M + H]^+^ 306.0124, found 306.0119. IR (KBr, νmax, cm^−1^): 3362, 3032, 1637, 1586, 1485, 1339, 1220.


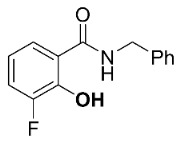


N-benzyl-3-fluoro-2-hydroxybenzamide **2f**. White solid. 41.9 mg, 57% yield. R_f_ = 0.55 (EtOAc/PE = 1:4). ^1^H NMR (400 MHz, CDCl_3_) δ 12.39 (s, 1H), 7.36 (dd, *J* = 14.9, 6.8 Hz, 4H), 7.26–7.12 (m, 2H), 6.76 (dd, *J* = 12.7, 8.0 Hz, 1H), 6.67 (s, 1H), 4.63 (d, *J* = 5.5 Hz, 2H); ^13^C NMR (100 MHz, CDCl_3_) δ 169.2, 152.1 (d, *J* = 246.6 Hz), 150.4 (d, *J* = 13.2 Hz), 137.1 (s), 128.9 (s), 128.0, 127.9, 120.4 (d, *J* = 3.7 Hz), 120.0 (d, *J* = 17.7 Hz), 117.8 (d, *J* = 6.9 Hz), 116.2 (d, *J* = 3.5 Hz), 43.8. HRMS (ESI) *m*/*z* calcd for C_14_H_13_FNO_2_ [M + H]^+^ 246.0925, found 246.0921. IR (KBr, νmax, cm^−1^): 3380, 2945, 1650, 1600, 1462, 1250.


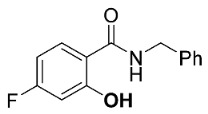


N-benzyl-4-fluoro-2-hydroxybenzamide **2g**. White solid. 50.7 mg, 69% yield. R_f_ = 0.55 (EtOAc/PE = 1:4). ^1^H NMR (400 MHz, CDCl_3_) δ 12.66 (s, 1H), 7.46–7.28 (m, 6H), 6.67 (dd, *J* = 10.3, 2.3 Hz, 1H), 6.59–6.35 (m, 2H), 4.62 (d, *J* = 5.5 Hz, 2H); ^13^C NMR (100 MHz, CDCl_3_) δ 169.2, 166.2 (d, *J* = 253.1 Hz), 163.9 (d, *J* = 13.7 Hz), 137.2, 128.9, 127.9, 127.9, 127.2 (d, *J* = 11.3 Hz), 110.8 (d, *J* = 2.7 Hz), 106.6 (d, *J* = 22.9 Hz), 105.3 (d, *J* = 23.6 Hz), 43.7. The characterization data is in accordance with that reported in the literature [23].


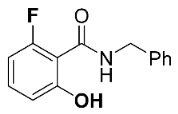


N-benzyl-2-fluoro-6-hydroxybenzamide **2h**. White solid. 70.6 mg, 96% yield. R_f_ = 0.55 (EtOAc/PE = 1:4). ^1^H NMR (400 MHz, CDCl_3_) δ 13.27 (s, 1H), 7.43–7.31 (m, 7H), 6.83 (d, *J* = 8.4 Hz, 1H), 6.60 (dd, *J* = 12.9, 8.2 Hz, 1H), 4.69 (d, *J* = 4.6 Hz, 2H); ^13^C NMR (100 MHz, CDCl_3_) δ 167.9, 163.7 (d, *J* = 4.6 Hz), 161.4 (d, *J* = 246.2 Hz), 137.2, 133.7 (d, *J* = 13.6 Hz), 128.8, 127.8, 127.7, 114.7 (d, *J* = 2.8 Hz), 105.5 (d, *J* = 25.8 Hz), 103.4 (d, *J* = 11.8 Hz), 43.6. HRMS (ESI) *m*/*z* calcd for C_14_H_13_FNO_2_ [M + H]^+^ 246.0925, found 246.0923. IR (KBr, νmax, cm^−1^): 3398, 3032, 1650, 1600, 1252, 1037.


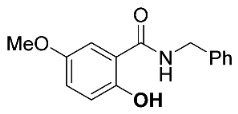


N-benzyl-2-hydroxy-5-methoxybenzamide **2i**. White solid. 62.5 mg, 81% yield. R_f_ = 0.40 (EtOAc/PE = 1:4). ^1^H NMR (400 MHz, CDCl_3_) δ 12.66 (s, 1H), 7.43–7.28 (m, 5H), 7.24 (d, *J* = 9.2 Hz, 1H), 6.47 (s, 1H), 6.38 (d, *J* = 8.8 Hz, 2H), 4.61 (d, *J* = 5.4 Hz, 2H), 3.80 (s, 3H). ^13^C NMR (100 MHz, CDCl_3_) δ 169.7, 164.4, 163.9, 137.6, 128.9, 127.9, 127.8, 126.5, 107.0, 107.0, 101.6, 55.4, 43.6. The characterization data is in accordance with that reported in the literature [23].


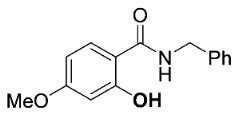


N-benzyl-2-hydroxy-4-methoxybenzamide **2j**. White solid. 63.3 mg, 82% yield. R_f_ = 0.40 (EtOAc/PE = 1:4). ^1^H NMR (400 MHz, CDCl_3_) δ 12.67 (s, 1H), 7.43–7.28 (m, 5H), 7.26 (d, *J* = 2.8 Hz, 1H), 6.47 (d, *J* = 2.4 Hz, 1H), 6.38 (dd, *J* = 8.8, 2.5 Hz, 2H), 4.62 (d, *J* = 5.5 Hz, 2H), 3.81 (s, 3H). ^13^C NMR (100 MHz, CDCl_3_) δ 169.7, 164.4, 163.9, 137.6, 128.9, 127.9, 127.8, 126.5, 126.5, 107.0, 101.6, 55.4, 43.6. The characterization data is in accordance with that reported in the literature [39].


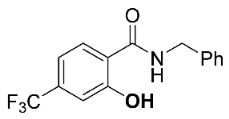


N-benzyl-2-hydroxy-4-(trifluoromethyl)benzamide **2k**. White solid. 72.6 mg, 82% yield. R_f_ = 0.60 (EtOAc/PE = 1:4). ^1^H NMR (400 MHz, CDCl_3_) δ 12.46 (s, 1H), 7.45 (d, *J* = 8.3 Hz, 1H), 7.43–7.28 (m, 5H), 7.25 (s, 1H), 7.05 (d, *J* = 8.2 Hz, 1H), 6.66 (s, 1H), 4.64 (d, *J* = 5.5 Hz, 2H); ^13^C NMR (101 MHz, CDCl_3_) δ 168.8, 161.7, 136.9, 135.8 (dd, *J* = 65.9, 33.1 Hz), 129.0, 128.1, 127.9, 126.1, 123.2 (d, *J* = 272.9 Hz), 116.8, 116.0 (d, *J* = 3.8 Hz), 115.0 (d, *J* = 3.7 Hz), 43.9. The characterization data is in accordance with that reported in the literature [40].


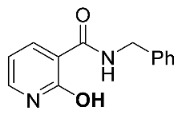


N-benzyl-2-hydroxynicotinamide **2l**. White solid. 46.7 mg, 68% yield. R_f_ = 0.50 (EtOAc/PE = 1:1). ^1^H NMR (400 MHz, CDCl_3_) δ 12.66 (s, 1H), 9.94 (s, 1H), 8.68 (s, 1H), 7.46 (s, 1H), 7.35 (d, *J* = 7.5 Hz, 4H), 7.26 (s, 1H), 6.53 (s, 1H), 4.69 (s, 2H); ^13^C NMR (100 MHz, CDCl_3_) δ 163.7, 145.6, 138.6, 137.5, 128.6, 128.6, 127.5, 127.2, 121.7, 108.0, 43.4. The characterization data is in accordance with that reported in the literature [23].


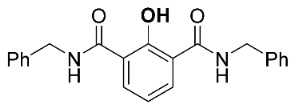


N^1^,N^3^-dibenzyl-2-hydroxyisophthalamide **2m**. White solid. 95.1 mg, 88% yield. R_f_ = 0.55 (EtOAc/PE = 1:2). ^1^H NMR (400 MHz, CDCl_3_) δ 14.46 (s, 1H), 7.97 (d, *J* = 7.2 Hz, 4H), 7.43–7.24 (m, 10H), 6.88 (t, *J* = 7.4 Hz, 1H), 4.64 (d, *J* = 5.2 Hz, 4H); ^13^C NMR (100 MHz, CDCl_3_) δ 167.5, 160.5, 137.7, 133.1, 128.7, 127.7, 127.6, 118.6, 117.9, 43.8. The characterization data is in accordance with that reported in the literature [23].


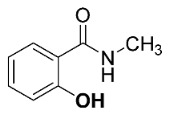


2-Hydroxy-N-methylbenzamide **2n**. Brown solid. 36.4 mg, 80% yield. R_f_ = 0.60 (EtOAc/PE = 1:2). ^1^H NMR (400 MHz, CDCl_3_) δ 12.43 (s, 1H), 7.39 (t, *J* = 7.8 Hz, 2H), 6.98 (d, *J* = 8.4 Hz, 1H), 6.84 (t, *J* = 7.6 Hz, 1H), 6.67 (s, 1H), 3.00 (d, *J* = 4.8 Hz, 3H); ^13^C NMR (100 MHz, CDCl_3_) δ 170.6, 161.2, 134.1, 125.4, 118.7, 118.4, 114.3, 26.4. The characterization data is in accordance with that reported in the literature [36].


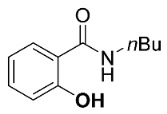


N-butyl-2-hydroxybenzamide **2o**. R_f_ = 0.50 (EtOAc/PE = 1:4). White solid. 47.3 mg, 82% yield. ^1^H NMR (400 MHz, CDCl_3_) δ 12.43 (s, 1H), 7.38 (t, *J* = 7.2 Hz, 2H), 6.97 (d, *J* = 8.4 Hz, 1H), 6.82 (t, *J* = 7.5 Hz, 1H), 6.46 (s, 1H), 3.44 (dd, *J* = 13.3, 6.6 Hz, 2H), 1.61 (dt, *J* = 14.7, 7.2 Hz, 2H), 1.41 (dd, *J* = 14.9, 7.4 Hz, 2H), 0.96 (t, *J* = 7.3 Hz, 3H); ^13^C NMR (100 MHz, CDCl_3_) δ 169.9, 161.5, 134.0, 125.3, 118.6, 118.5, 114.4, 39.4, 31.5, 20.1, 13.7. The characterization data is in accordance with that reported in the literature [41].


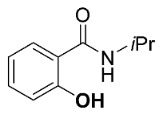


2-Hydroxy-N-isopropylbenzamide **2p**. White solid. 50.8 mg, 95% yield. R_f_ = 0.55 (EtOAc/PE = 1:4). ^1^H NMR (400 MHz, CDCl_3_) δ 12.44 (s, 1H), 7.45–7.28 (m, 2H), 6.98 (d, *J* = 8.3 Hz, 1H), 6.83 (t, *J* = 7.5 Hz, 1H), 6.12 (s, 1H), 4.28 (dq, *J* = 13.4, 6.6 Hz, 1H), 1.28 (d, *J* = 6.5 Hz, 6H); ^13^C NMR (100 MHz, CDCl_3_) δ 169.2, 161.6, 134.0, 125.1, 118.6, 118.5, 114.4, 41.8, 22.7. The characterization data is in accordance with that reported in the literature [36].


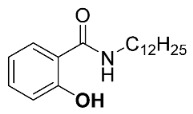


N-dodecyl-2-hydroxybenzamide **2q**. White solid. 75.0 mg, 82% yield. R_f_ = 0.70 (EtOAc/PE = 1:4). ^1^H NMR (400 MHz, CDCl_3_) δ 12.40 (s, 1H), 7.43–7.31 (m, 2H), 6.98 (d, *J* = 8.3 Hz, 1H), 6.83 (t, *J* = 7.3 Hz, 1H), 6.33 (s, 1H), 3.44 (dd, *J* = 13.0, 6.5 Hz, 2H), 1.61 (d, *J* = 7.0 Hz, 3H), 1.30 (d, *J* = 31.8 Hz, 18H), 0.88 (t, *J* = 6.1 Hz, 3H); ^13^C NMR (100 MHz, CDCl_3_) δ 169.9, 161.6, 134.1, 125.1, 118.6, 118.5, 114.4, 39.7, 31.9, 29.6, 29.6, 29.6, 29.5, 29.5, 29.3, 29.3, 26.9, 22.7, 14.1. The characterization data is in accordance with that reported in the literature [42].


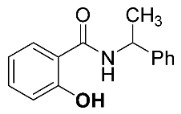


2-Hydroxy-N-(1-phenylethyl)benzamide **2r**. White solid. 61.4 mg, 85% yield. R_f_ = 0.60 (EtOAc/PE = 1:4). ^1^H NMR (400 MHz, CDCl_3_) δ 12.30 (s, 1H), 7.37 (d, *J* = 4.2 Hz, 6H), 7.29 (d, *J* = 3.7 Hz, 1H), 6.97 (d, *J* = 8.4 Hz, 1H), 6.81 (t, *J* = 7.4 Hz, 1H), 6.54 (s, 1H), 5.30 (p, *J* = 6.9 Hz, 1H), 1.61 (d, *J* = 6.8 Hz, 3H); ^13^C NMR (100 MHz, CDCl_3_) δ 169.1, 161.6, 142.5, 134.2, 128.8, 127.7, 126.1, 125.3, 118.6, 118.6, 114.2, 49.0, 21.6. The characterization data is in accordance with that reported in the literature [43].


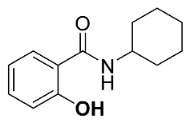


N-cyclohexyl-2-hydroxybenzamide **2s**. White solid. 62.9 mg, 96% yield. R_f_ = 0.55 (EtOAc/PE = 1:4). ^1^H NMR (400 MHz, CDCl_3_) δ 12.44 (s, 1H), 7.42–7.29 (m, 2H), 6.98 (d, *J* = 8.3 Hz, 1H), 6.83 (t, *J* = 7.5 Hz, 1H), 6.15 (s, 1H), 3.96 (d, *J* = 7.8 Hz, 1H), 2.03 (d, *J* = 12.5 Hz, 2H), 1.78 (d, *J* = 13.1 Hz, 2H), 1.67 (d, *J* = 12.8 Hz, 1H), 1.43 (dd, *J* = 24.2, 12.7 Hz, 2H), 1.30–1.19 (m, 3H); ^13^C NMR (100 MHz, CDCl_3_) δ 169.1, 161.6, 134.0, 125.1, 118.6, 118.5, 114.4, 48.5, 33.0, 25.5, 24.8. The characterization data is in accordance with that reported in the literature [44].


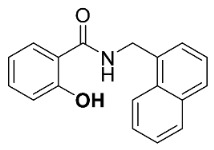


2-Hydroxy-N-(naphthalen-1-ylmethyl)benzamide **2t**. White solid. 52.4 mg, 63% yield. R_f_ = 0.65 (EtOAc/PE = 1:4). ^1^H NMR (400 MHz, CDCl_3_) δ 12.34 (s, 1H), 8.04 (d, *J* = 8.1 Hz, 1H), 7.88 (dd, *J* = 17.9, 7.9 Hz, 2H), 7.51 (qd, *J* = 14.6, 7.0 Hz, 4H), 7.37 (t, *J* = 7.7 Hz, 1H), 7.25 (s, 1H), 7.00 (d, *J* = 8.3 Hz, 1H), 6.75 (t, *J* = 7.5 Hz, 1H), 6.50 (s, 1H), 5.08 (d, *J* = 4.9 Hz, 2H); ^13^C NMR (100 MHz, CDCl_3_) δ 169.6, 161.7, 134.3, 134.0, 132.5, 131.4, 129.1, 128.9, 127.1, 126.9, 126.2, 125.4, 125.4, 123.2, 118.7, 118.6, 114.0, 41.9. HRMS (ESI) *m*/*z* calcd for C_18_H_16_NO_2_ [M + H]^+^ 278.1176, found 278.1170. IR (KBr, νmax, cm^−1^): 3307, 3069, 1636, 1595, 1545, 1344.


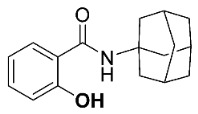


N-((3s,5s,7s)-adamantan-1-yl)-2-hydroxybenzamide **2u**. White solid. 47.3 mg, 58% yield. R_f_ = 0.70 (EtOAc/PE = 1:4). ^1^H NMR (400 MHz, CDCl_3_) δ 12.49 (s, 1H), 7.39–7.32 (m, 1H), 7.28 (d, *J* = 8.0 Hz, 1H), 6.96 (d, *J* = 8.3 Hz, 1H), 6.81 (t, *J* = 7.6 Hz, 1H), 5.96 (s, 1H), 2.13 (s, 9H), 1.73 (s, 6H); ^13^C NMR (100 MHz, CDCl_3_) δ 169.5, 161.7, 133.8, 125.2, 118.7, 118.4, 115.2, 52.8, 41.6, 36.3, 29.4. The characterization data is in accordance with that reported in the literature [45].


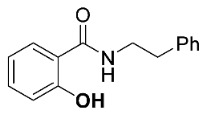


2-Hydroxy-N-phenethylbenzamide **2v**. White solid. 62.9 mg, 87% yield. R_f_ = 0.60 (EtOAc/PE = 1:4). ^1^H NMR (400 MHz, CDCl_3_) δ 12.35 (s, 1H), 7.43–7.30 (m, 3H), 7.28–7.16 (m, 4H), 6.97 (d, *J* = 8.3 Hz, 1H), 6.79 (t, *J* = 7.5 Hz, 1H), 6.36 (s, 1H), 3.70 (q, *J* = 6.3 Hz, 2H), 2.93 (t, *J* = 6.7 Hz, 2H); ^13^C NMR (100 MHz, CDCl_3_) δ 169.9, 161.5, 138.4, 134.2, 128.8, 128.7, 126.8, 125.1, 118.6, 118.6, 114.2, 40.7, 35.5. The characterization data is in accordance with that reported in the literature [46].


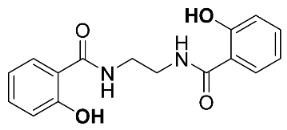


N,N’-(ethane-1,2-diyl)bis(2-hydroxybenzamide) **2w**. White solid. 50.4 mg, 56% yield. R_f_ = 0.70 (EtOAc/PE = 1:1). ^1^H NMR (400 MHz, CDCl_3_) δ 12.15 (s, 2H), 7.40 (t, *J* = 9.1 Hz, 6H), 6.97 (d, *J* = 8.2 Hz, 2H), 6.87 (t, *J* = 7.3 Hz, 2H), 3.72 (s, 4H), 1.66 (s, 2H); ^13^C NMR (100 MHz, CDCl_3_) δ 171.4 161.5, 134.6, 125.7, 119.0, 118.6, 113.7, 40.6. The characterization data is in accordance with that reported in the literature [23].


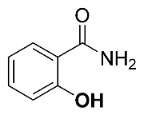


2-Hydroxybenzamide **2x**. 32.9 mg, 80% yield. Brown solid. R_f_ = 0.50 (EtOAc/PE = 1:2). ^1^H NMR (400 MHz, CDCl_3_) δ 12.41 (s, 1H), 7.37 (t, *J* = 7.8 Hz, 2H), 6.97 (d, *J* = 8.4 Hz, 1H), 6.82 (t, *J* = 7.6 Hz, 1H), 6.65 (s, 1H), 2.99 (d, *J* = 4.8 Hz, 3H); ^13^C NMR (100 MHz, CDCl_3_) δ 170.6, 161.2, 134.1, 125.4, 118.7, 118.4, 114.3, 26.4. The characterization data is in accordance with that reported in the literature [47].

### 3.3. General Procedure for the Synthesis of o-Alkoxybenzamides ***3***

A 10-mL round-bottom flask equipped with a magnetic stir bar was charged with 2-fluorobenzamide **1** (0.3 mmol, 1.0 equiv) and the corresponding sodium alkoxide (0.45 mmol, 1.5 equiv). Anhydrous DMSO (1 mL) was added, and the mixture was stirred at room temperature (25 °C) under air for 12 h. After completion, the reaction was quenched by the addition of water (10 mL). The aqueous phase was extracted with ethyl acetate (3 × 10 mL). The combined organic layers were washed with brine (10 mL), dried over anhydrous Na_2_SO_4_, filtered, and concentrated under reduced pressure. The residue was purified by flash column chromatography on silica gel (200–300 mesh) using a gradient elution of ethyl acetate in petroleum ether (typically from 1:10 to 1:2, *v*/*v*) to afford the desired *o*-alkoxybenzamide **3**.


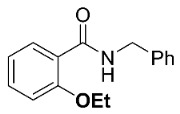


N-benzyl-2-ethoxybenzamide **3a**. Colorless oil. 70.5 mg, 87% yield. R_f_ = 0.50 (EtOAc/PE = 1:4). ^1^H NMR (400 MHz, CDCl_3_) δ 8.34 (s, 1H), 8.25 (dd, *J* = 7.8, 1.5 Hz, 1H), 7.43–7.32 (m, 5H), 7.28 (d, *J* = 6.8 Hz, 1H), 7.06 (t, *J* = 7.6 Hz, 1H), 6.92 (d, *J* = 8.3 Hz, 1H), 4.66 (d, *J* = 5.4 Hz, 2H), 4.11 (q, *J* = 7.0 Hz, 2H), 1.31 (t, *J* = 7.0 Hz, 3H); ^13^C NMR (100 MHz, CDCl3) δ 165.2, 156.9, 138.5, 132.7, 132.2, 128.6, 127.7, 127.3, 121.3, 121.2, 112.2, 64.6, 44.0, 14.6. The characterization data is in accordance with that reported in the literature [48].


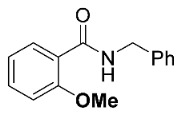


N-benzyl-2-methoxybenzamide **3b**. Colorless oil. 62.9 mg, 87% yield. R_f_ = 0.40 (EtOAc/PE = 1:4). ^1^H NMR (400 MHz, CDCl_3_) δ 8.25 (d, *J* = 7.7 Hz, 1H), 8.21 (s, 1H), 7.45 (t, *J* = 7.8 Hz, 1H), 7.40–7.30 (m, 4H), 7.29–7.24 (m, 1H), 7.09 (t, *J* = 7.5 Hz, 1H), 6.96 (d, *J* = 8.4 Hz, 1H), 4.69 (d, *J* = 5.7 Hz, 2H), 3.90 (s, 3H); ^13^C NMR (100 MHz, CDCl_3_) δ 165.3, 157.5, 138.8, 132.8, 132.4, 128.6, 127.5, 127.2, 121.4, 121.3, 111.3, 55.9, 43.7. The characterization data is in accordance with that reported in the literature [49].


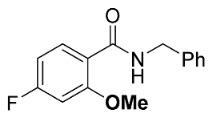


N-benzyl-4-fluoro-2-methoxybenzamide **3c**. Colorless oil. 70.0 mg, 90% yield. R_f_ = 0.40 (EtOAc/PE = 1:4). ^1^H NMR (400 MHz, CDCl_3_) δ 8.26 (dd, *J* = 8.8, 7.1 Hz, 1H), 8.05 (s, 1H), 7.34 (dd, *J* = 9.7, 3.7 Hz, 4H), 7.31–7.24 (m, 1H), 6.78 (ddd, *J* = 8.8, 7.8, 2.3 Hz, 1H), 6.68 (dd, *J* = 10.6, 2.3 Hz, 1H), 4.67 (d, *J* = 5.7 Hz, 2H), 3.90 (s, 3H); ^13^C NMR (100 MHz, CDCl_3_) δ 165.4 (d, *J* = 252.2 Hz), 164.4, 158.8 (d, *J* = 10.3 Hz), 138.6, 134.4 (d, *J* = 10.6 Hz), 128.6, 127.4, 127.2, 117.7 (d, *J* = 3.1 Hz), 108.1 (d, *J* = 21.1 Hz), 99.4 (d, *J* = 26.3 Hz), 56.2, 43.7. HRMS (ESI) *m*/*z* calcd for C_15_H_15_FNO_2_ [M + H]^+^ 260.1081, found 260.1079. IR (KBr, νmax, cm^−1^): 3407, 3028, 1659, 1600, 1500, 1270.


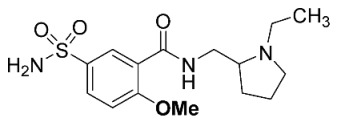


N-((1-ethylpyrrolidin-2-yl)methyl)-2-methoxy-5-sulfamoylbenzamide **3d**. White solid. 84.0 mg, 82% yield. R_f_ = 0.40 (MeOH/EtOAc = 1:10). ^1^H NMR (400 MHz, DMSO) δ 8.40–8.33 (m, 1H), 8.29 (s, 1H), 7.89 (dd, *J* = 8.7, 2.3 Hz, 1H), 7.40–7.27 (m, 3H), 3.97 (s, 3H), 3.51 (ddd, *J* = 13.2, 6.7, 3.0 Hz, 1H), 3.20–3.08 (m, 2H), 2.82 (td, *J* = 14.6, 7.3 Hz, 1H), 2.62–2.54 (m, 1H), 2.28–2.06 (m, 2H), 1.82 (ddd, *J* = 16.7, 11.7, 8.2 Hz, 1H), 1.71–1.58 (m, 2H), 1.57–1.48 (m, 1H), 1.06 (t, *J* = 7.1 Hz, 3H); ^13^C NMR (100 MHz, DMSO) δ 164.0, 159.7, 136.9, 130.3, 129.2, 123.2, 113.1, 62.4, 57.1, 53.7, 48.1, 42.2, 28.7, 23.0, 14.5. The characterization data is in accordance with that reported in the literature [50].

## 4. Conclusions

In summary, we have developed a practical transition-metal-free hydroxylation of o-fluorobenzamides using KO*t*-Bu and H_2_O under mild basic conditions, providing salicylamides with excellent ortho selectivity and broad functional group compatibility. Mechanistic studies suggest that the transformation critically depends on the amide N–H functionality and is consistent with a hydrogen-bond-assisted and conformationally controlled S_N_Ar process. The protocol also exhibits notable substrate-dependent reactivity, as *meta*-/*para*-fluorobenzamides and N-aryl/N,N-disubstituted amides remain unreactive under the standard conditions. In addition, the method could be extended to alkoxylation reactions, enabling efficient access to *o*-alkoxybenzamides, including biologically relevant molecules such as sulpiride. Owing to its operational simplicity, inexpensive reagents, and transition-metal-free nature, this method provides a useful approach to salicylamides and related derivatives.

## Figures and Tables

**Table 1:** Optimization of reaction conditions ^a^.

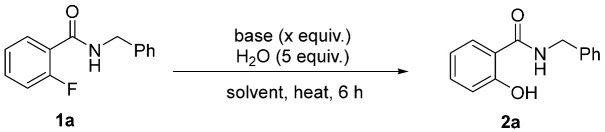					
Entry	Base	Equiv	Solvent	T (°C)	Yield(%) ^b^
1	KOH	3.0	DMSO	120	84
2	NaOH	3.0	DMSO	120	80
3	K_2_CO_3_	3.0	DMSO	120	15
4	Cs_2_CO_3_	3.0	DMSO	120	25
5	KOt-Bu	3.0	DMSO	120	85
6	NaH	3.0	DMSO	120	53
7	DBU	3.0	DMSO	120	0
8	Et_3_N	3.0	DMSO	120	0
9	KO*t*-Bu	1.0	DMSO	120	68
10	KO*t*-Bu	2.0	DMSO	120	77
11	KO*t*-Bu	4.0	DMSO	120	85
12	KO*t*-Bu	3.0	DMF	120	61
13	KO*t*-Bu	3.0	NMP	120	72
14	KO*t*-Bu	3.0	1,4-Dioxane	120	0
15	KO*t*-Bu	3.0	Toluene	120	0
16	KO*t*-Bu	3.0	*t*-BuOH	120	0
17	KO*t*-Bu	3.0	DMSO	110	86
18	KO*t*-Bu	3.0	DMSO	100	87
19 ^c^	KO*t*-Bu	3.0	DMSO	90	81
20 ^c^	KO*t*-Bu	3.0	DMSO	80	23
21 ^d^	KO*t*-Bu	3.0	DMSO	100	68
22 ^e^	KO*t*-Bu	3.0	DMSO	100	trace

^a^ Reaction conditions: **1a** (0.2 mmol), base (x equiv), H_2_O (1.0 mmol) in 1 mL solvent, heat for 6 h under a nitrogen atmosphere. ^b^ Isolated yields. ^c^ For 12 h. ^d^ Under air. ^e^ Without H_2_O. DBU = 1,8-diazabicyclo[5.4.0]undecane-7-ene. DMF = *N*,*N*-dimethylformamide. DMSO = dimethylsulfoxide. NMP = *N*-methyl-2-pyrrolidone.

## Data Availability

The original contributions presented in this study are included in the article/Supplementary Materials. Further inquiries can be directed to the corresponding author.

## Supplementary Materials

The following supporting information can be downloaded at: https://www.mdpi.com/article/10.3390/molecules31111887/s1. The copies of the NMR (^1^H, ^13^C) spectra of all products and HRMS spectra of the new compounds are available in the online Supplementary Materials.
