# Supplementary material for: Hydrogen-Bond-Assisted Hydroxylation of o-Fluorobenzamides: A Transition-Metal-Free Approach to Salicylamides
Source: Molecules. 2026 Jun 1;31(11):1887. doi: 10.3390/molecules31111887 (PMC13257559; doi:10.3390/molecules31111887)

Hydrogen-Bond-Assisted Hydroxylation of *o*-Fluorobenzamides: A Transition-Metal-Free Approach to Salicylamides

Ting Chen [1], Huiwen Lei [1], Ting Huang [1], Hanjun Chen [1], Shuo Li [1], Fuqiang Liu [2], Hai-Chao Xu [3] and Jinhai Shen [1,4,*]

[1] College of Environment and Public Health, Xiamen Huaxia University, Xiamen 361024, China; chent@hxxy.edu.cn (T.C.); leihwen@163.com (H.L.); 15207041950@163.com (T.H.); chenhanj5@126.com (H.C.); 15943889975@163.com (S.L.)
[2] College of Chemistry, Fuzhou University, Fuzhou 350108, China; lfqsmiling@126.com
[3] College of Chemistry and Chemical Engineering, Xiamen University, Xiamen 361005, China
[4] Fujian Key Laboratory of Molecular Medicine (Huaqiao University), Xiamen 361021, China
* Correspondence: shenjh@hxxy.edu.cn; Tel.: +86-592-6276212

Context

1. ^{1}H NMR and ^{13}C{^{1}H} NMR spectra of products --S2

2. HRMS spectra of the new compounds ---S58

1. ^{1}H NMR and ^{13}C{^{1}H} NMR spectra of products:

^{1}H NMR (400 MHz, $CDCl_3$) spectrum of **2a**

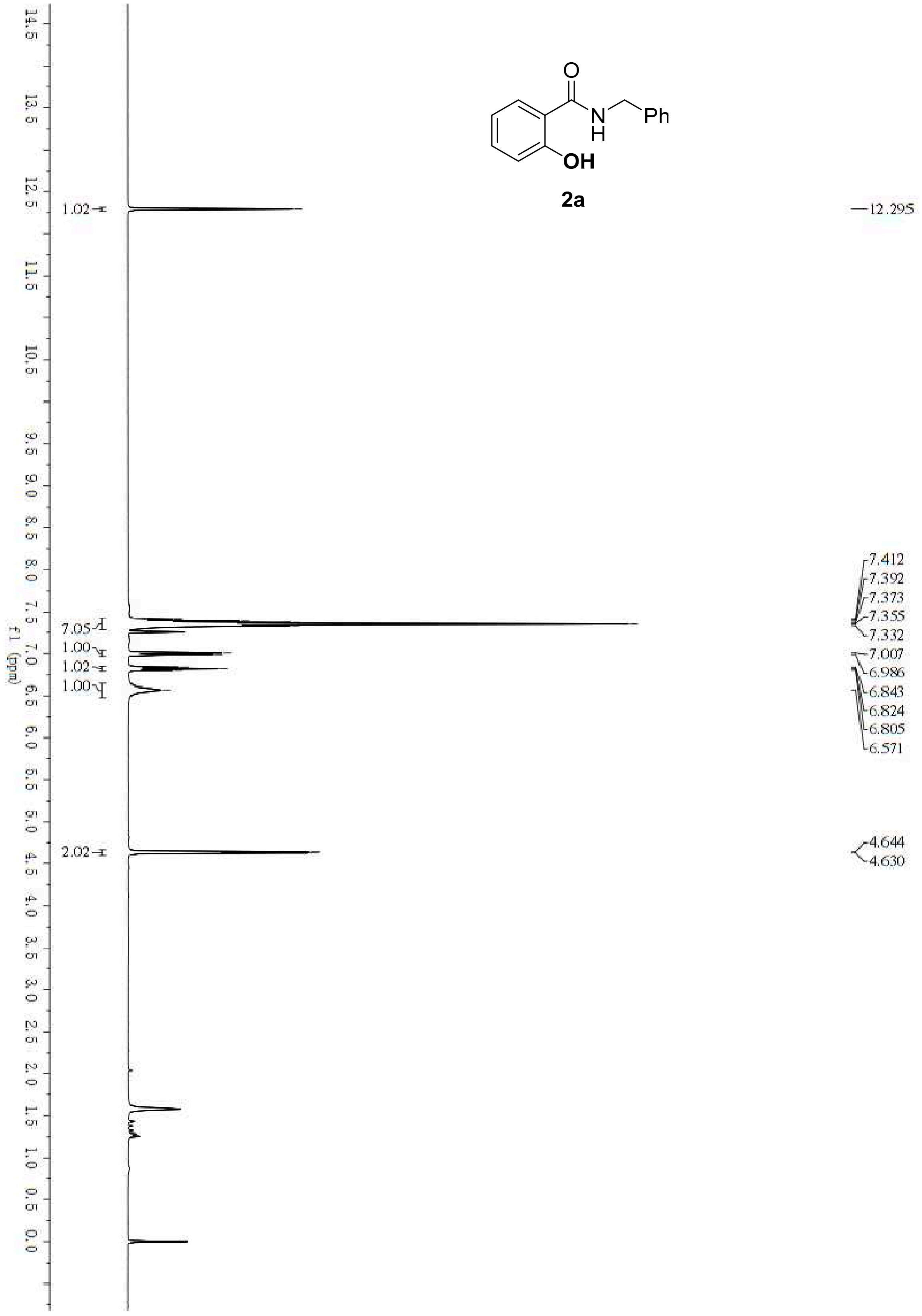

^{13}C {^{1}H} NMR (100 MHz, $CDCl_3$) spectrum of **2a**

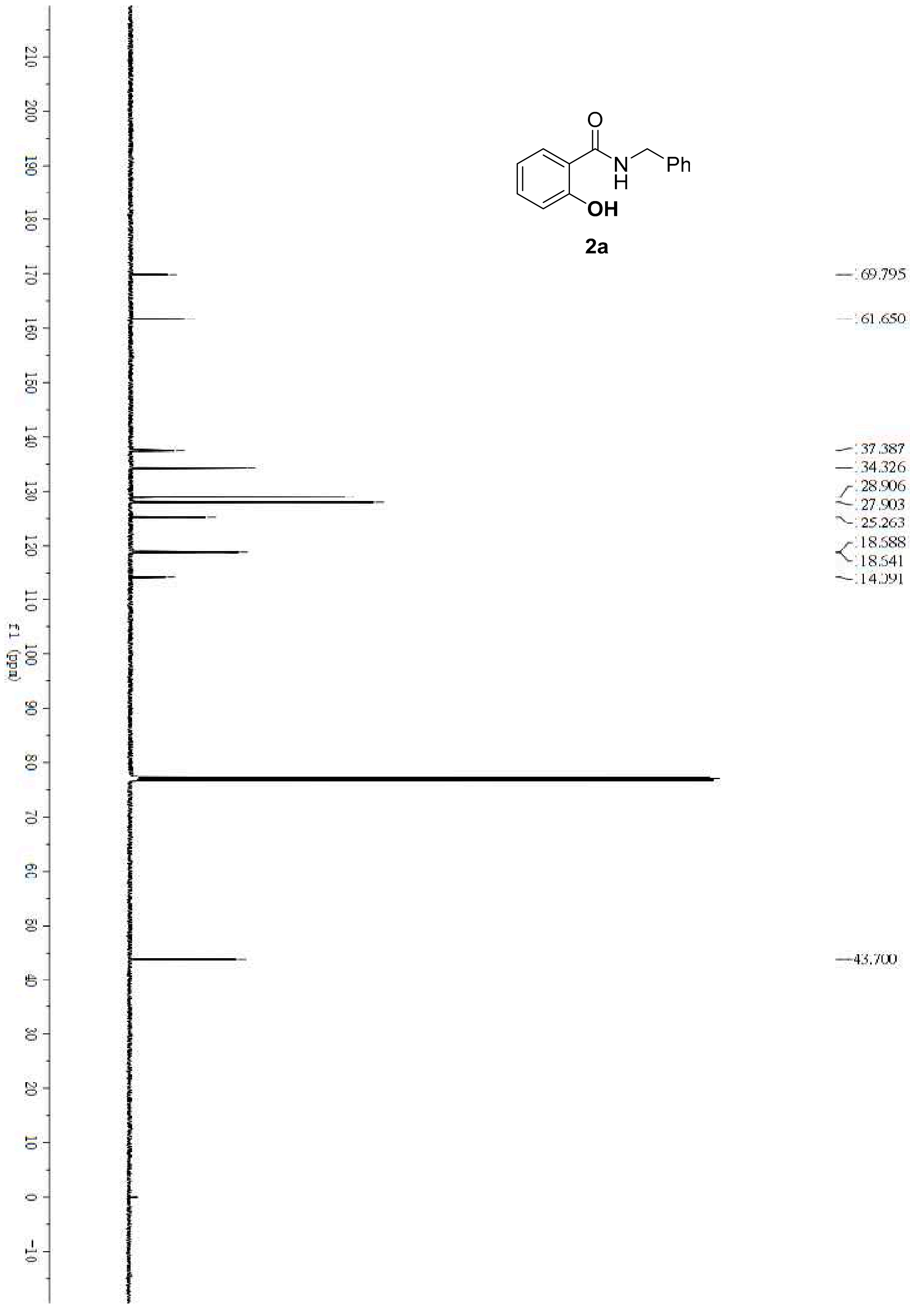

^{1}H NMR (400 MHz, $CDCl_3$) spectrum of **2b**

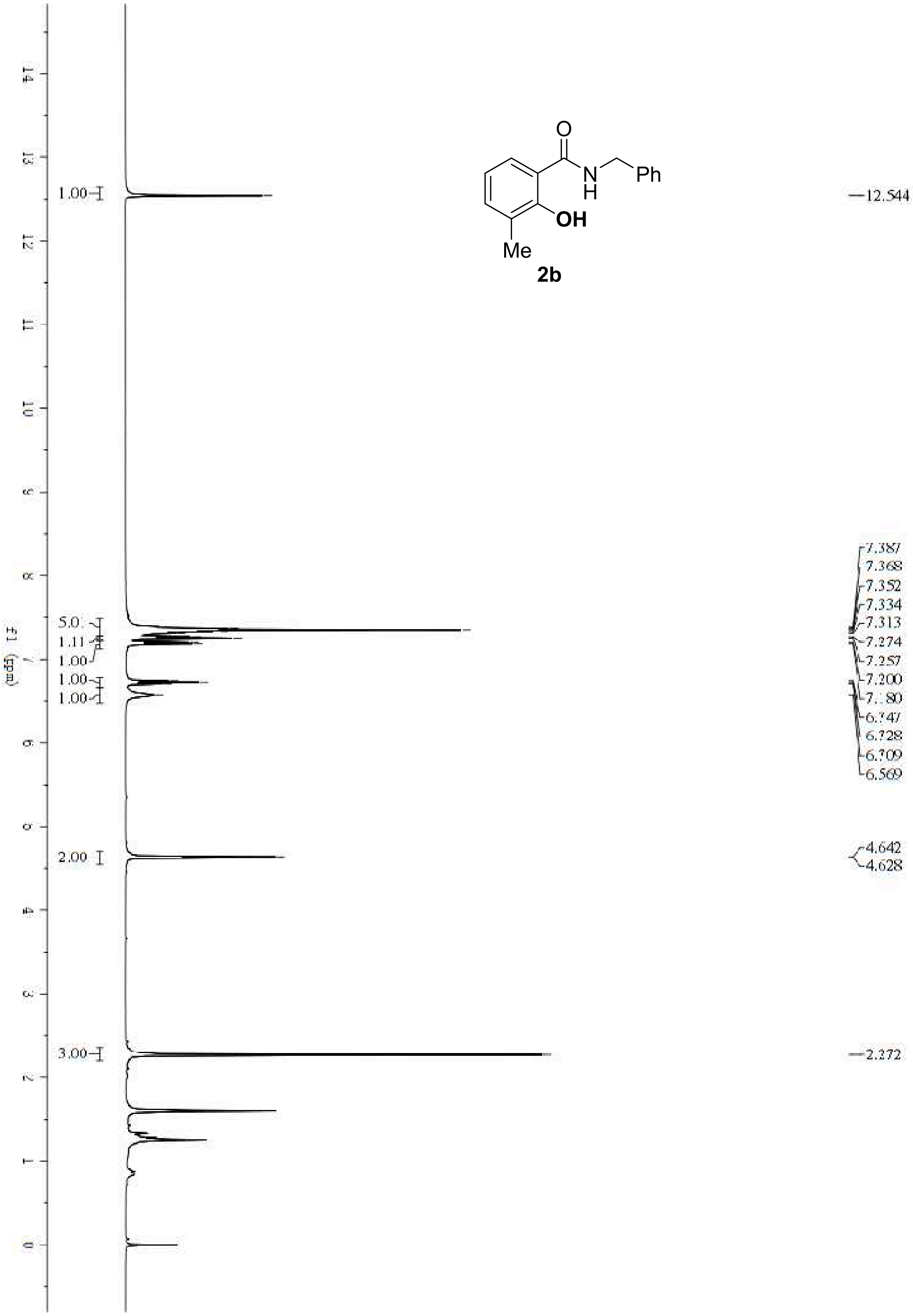

^{13}C {^{1}H} NMR (100 MHz, $CDCl_3$) spectrum of **2b**

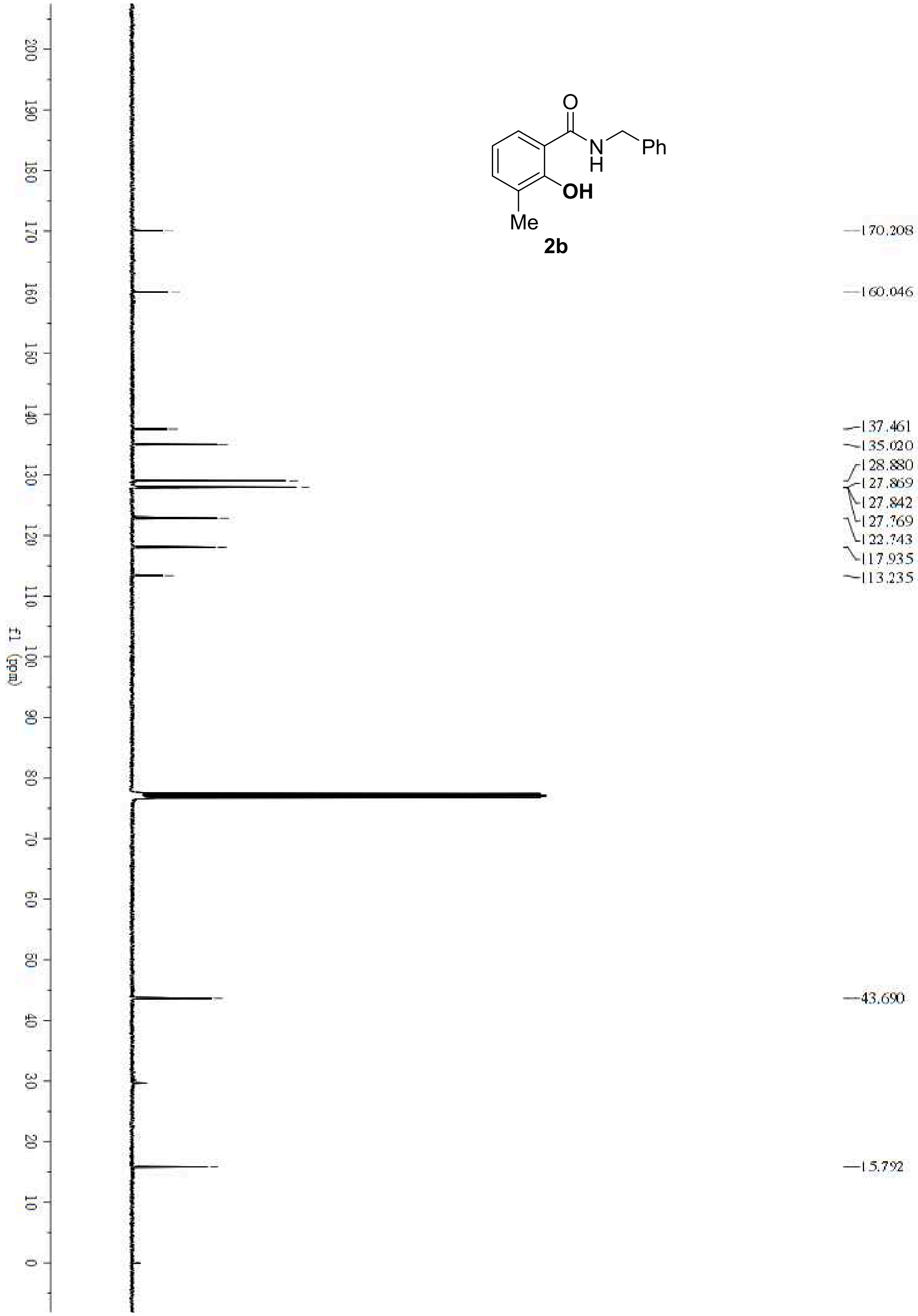

^{1}H NMR (400 MHz, $CDCl_3$) spectrum of **2c**

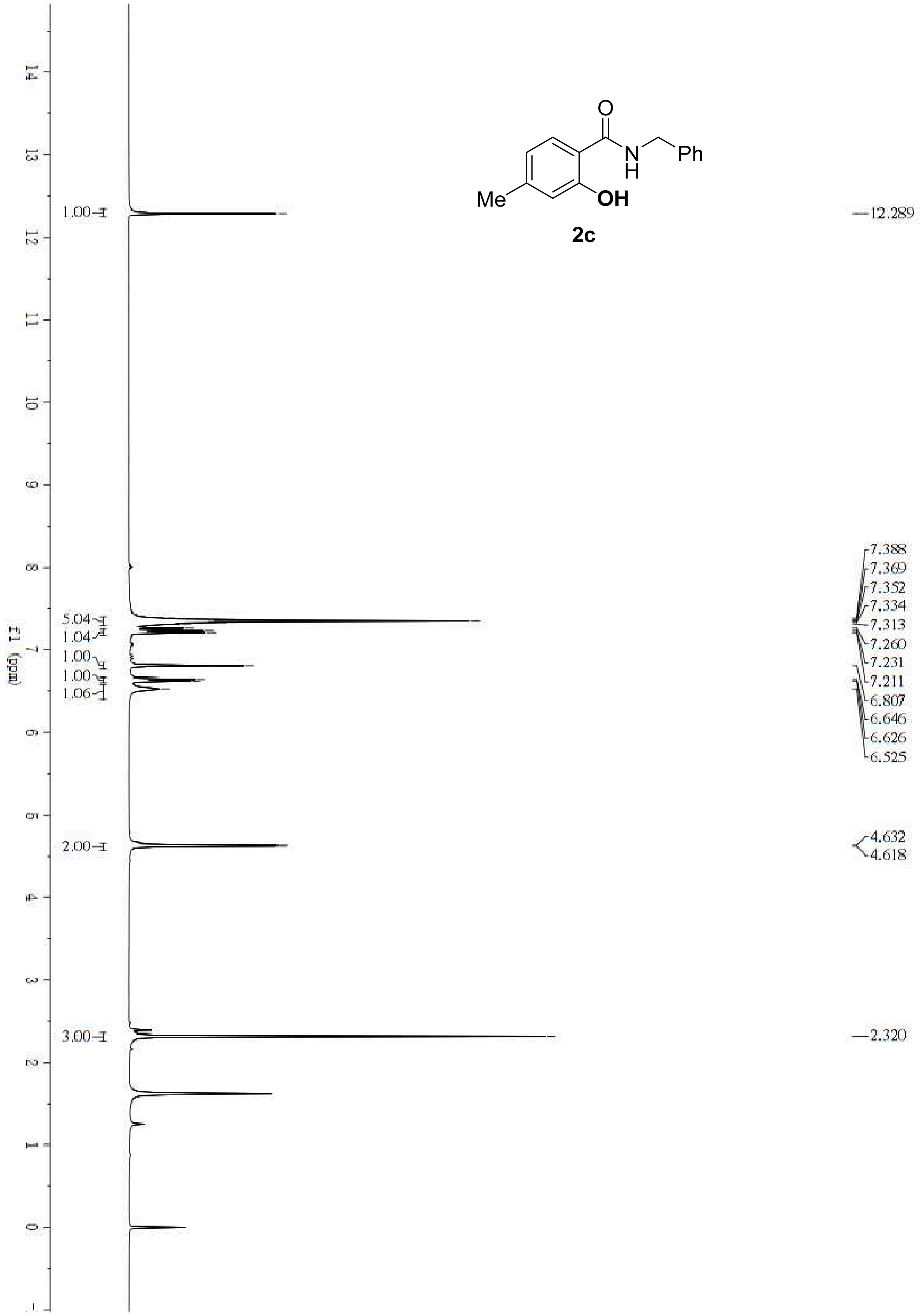

^{13}C {^{1}H} NMR (100 MHz, $CDCl_3$) spectrum of **2c**

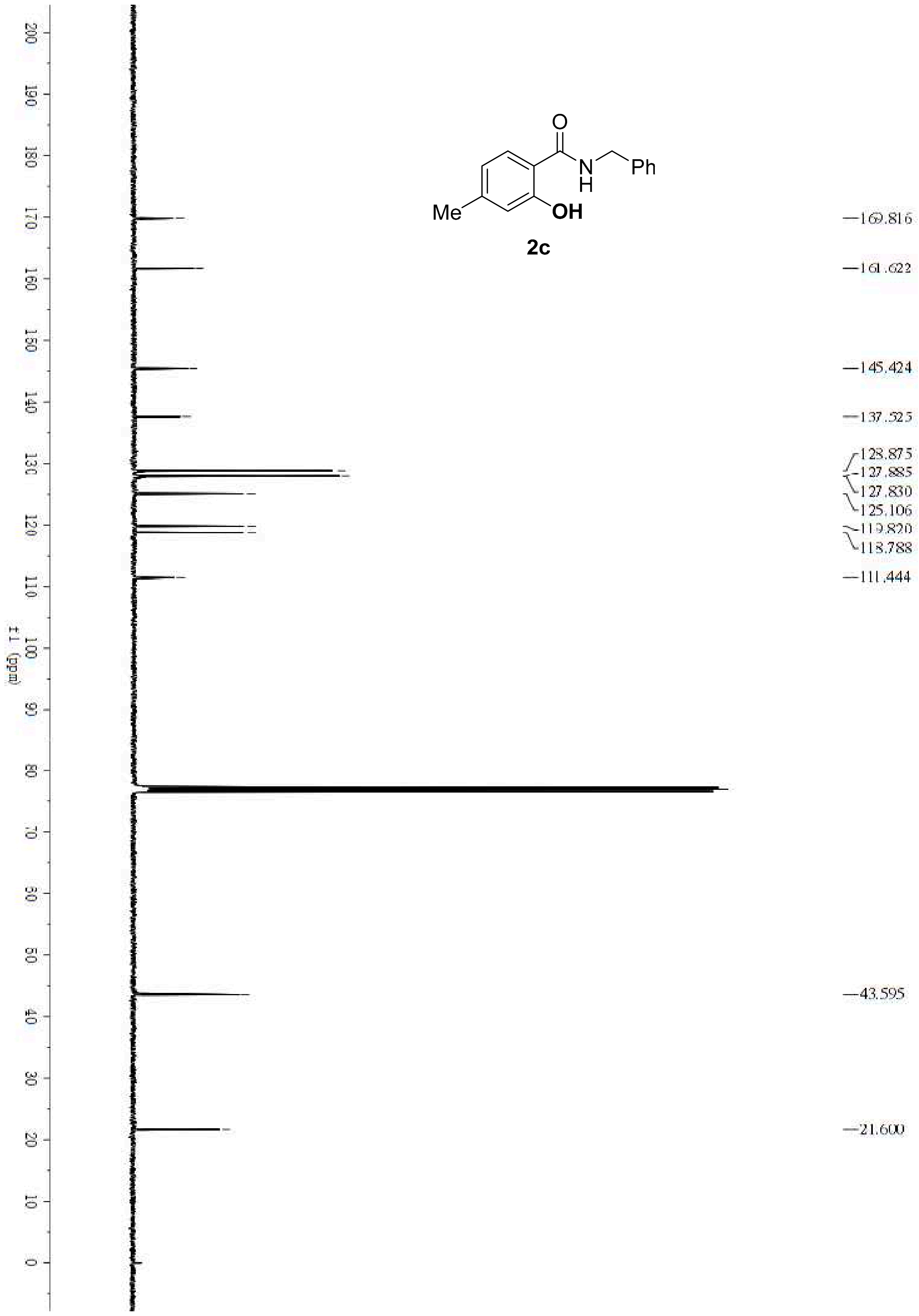

^{1}H NMR (400 MHz, $CDCl_3$) spectrum of **2d**

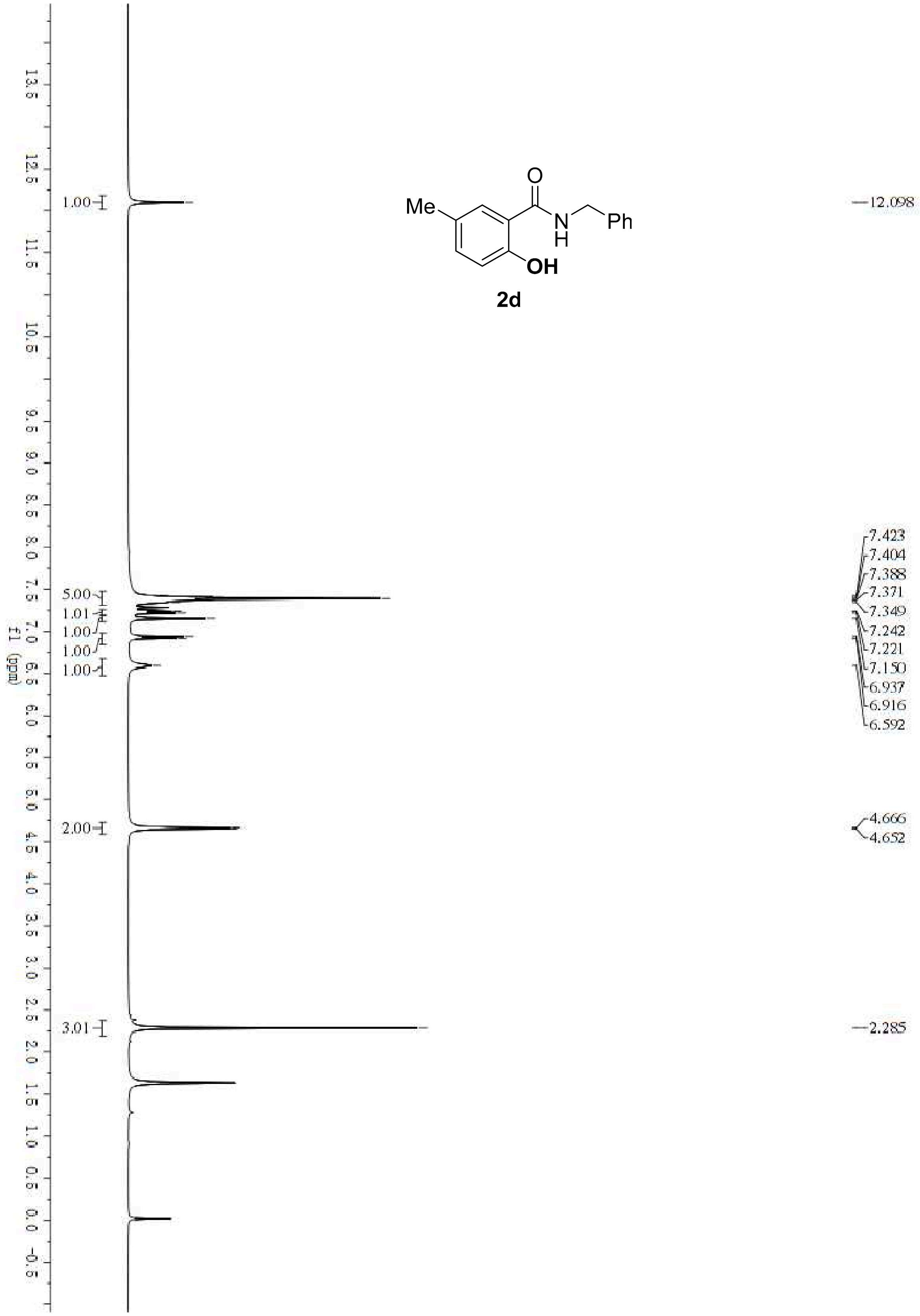

^{13}C {^{1}H} NMR (100 MHz, $CDCl_3$) spectrum of **2d**

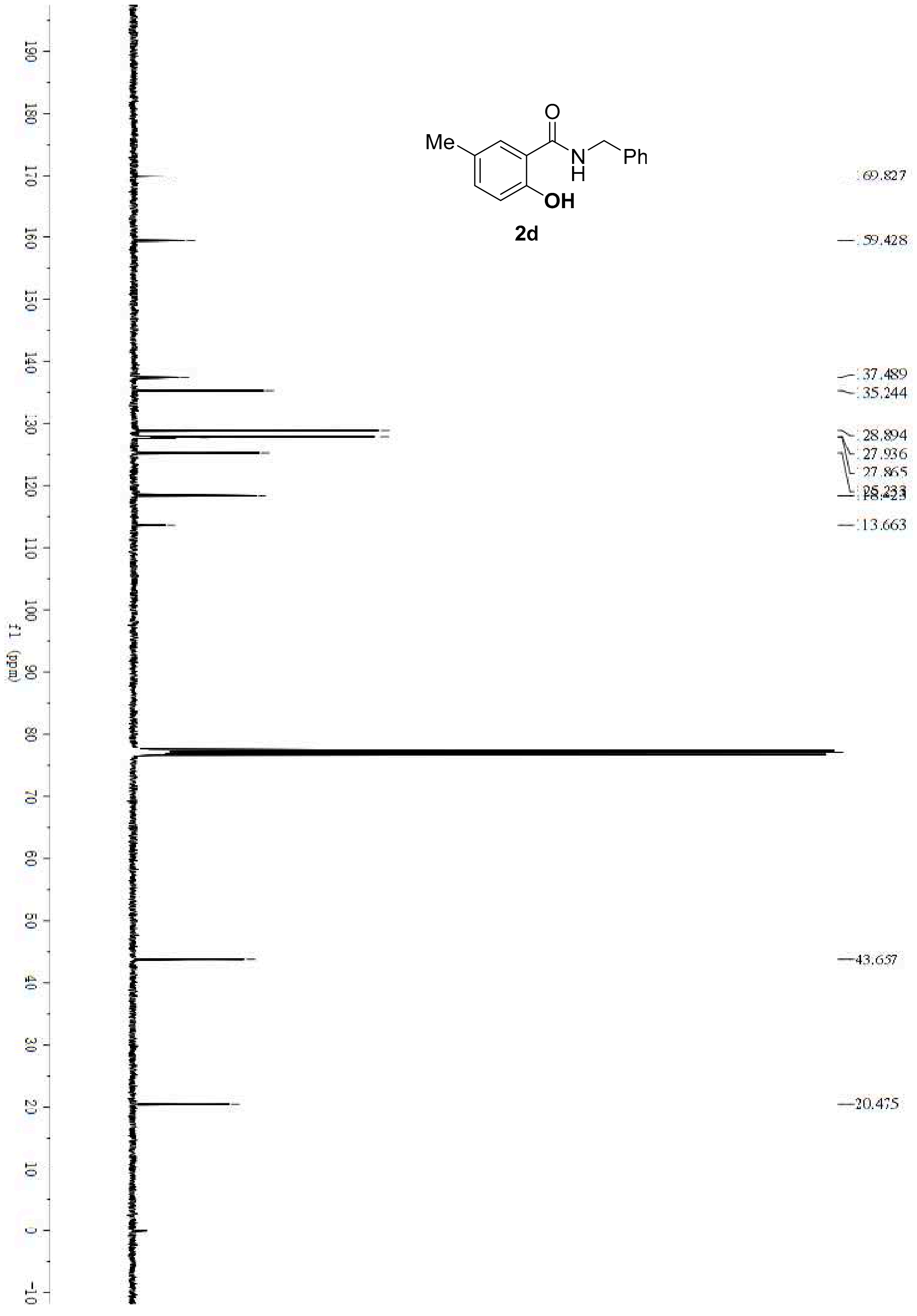

^{1}H NMR (400 MHz, $CDCl_3$) spectrum of **2e**

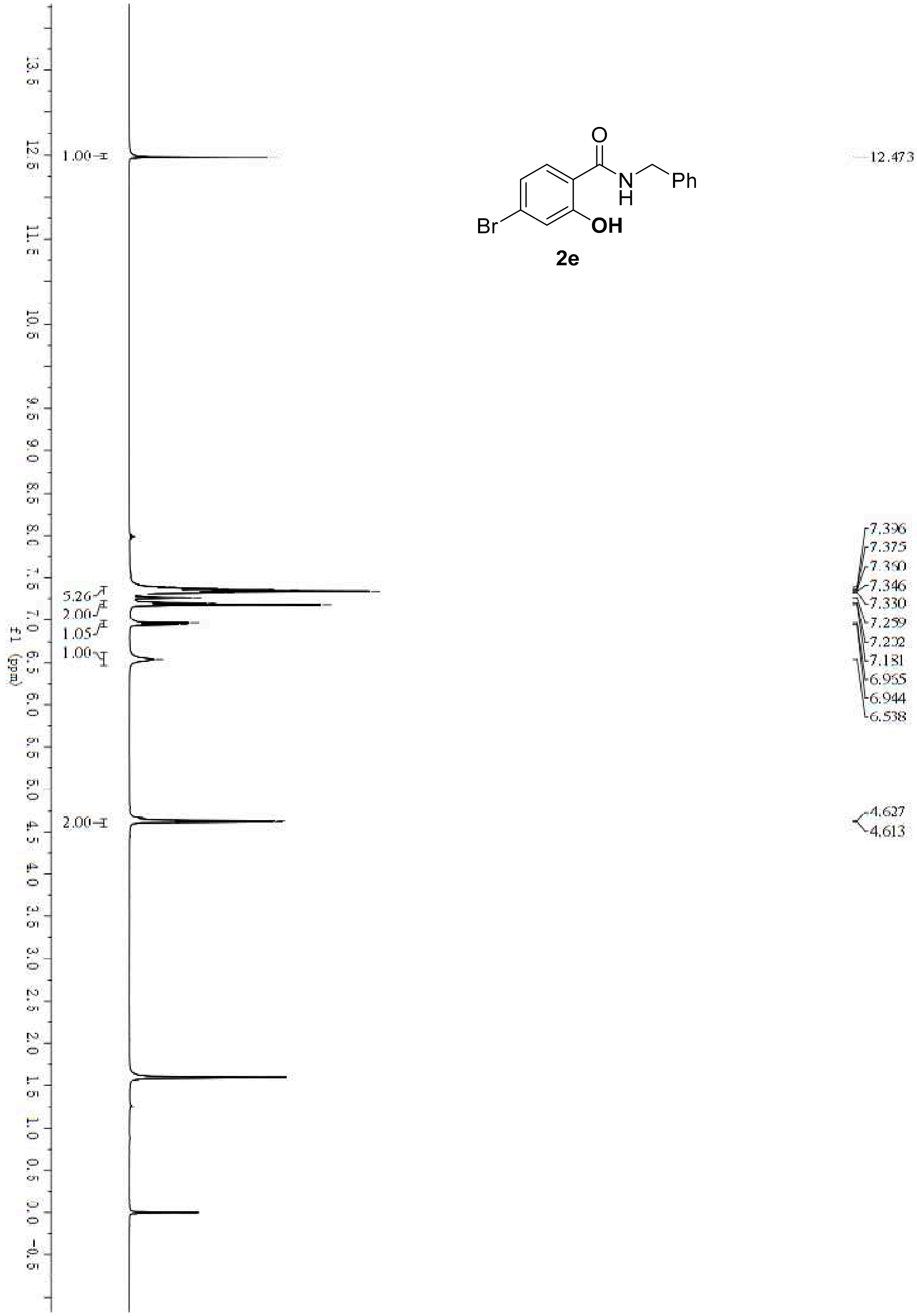

^{13}C {^{1}H} NMR (100 MHz, $CDCl_3$) spectrum of **2e**

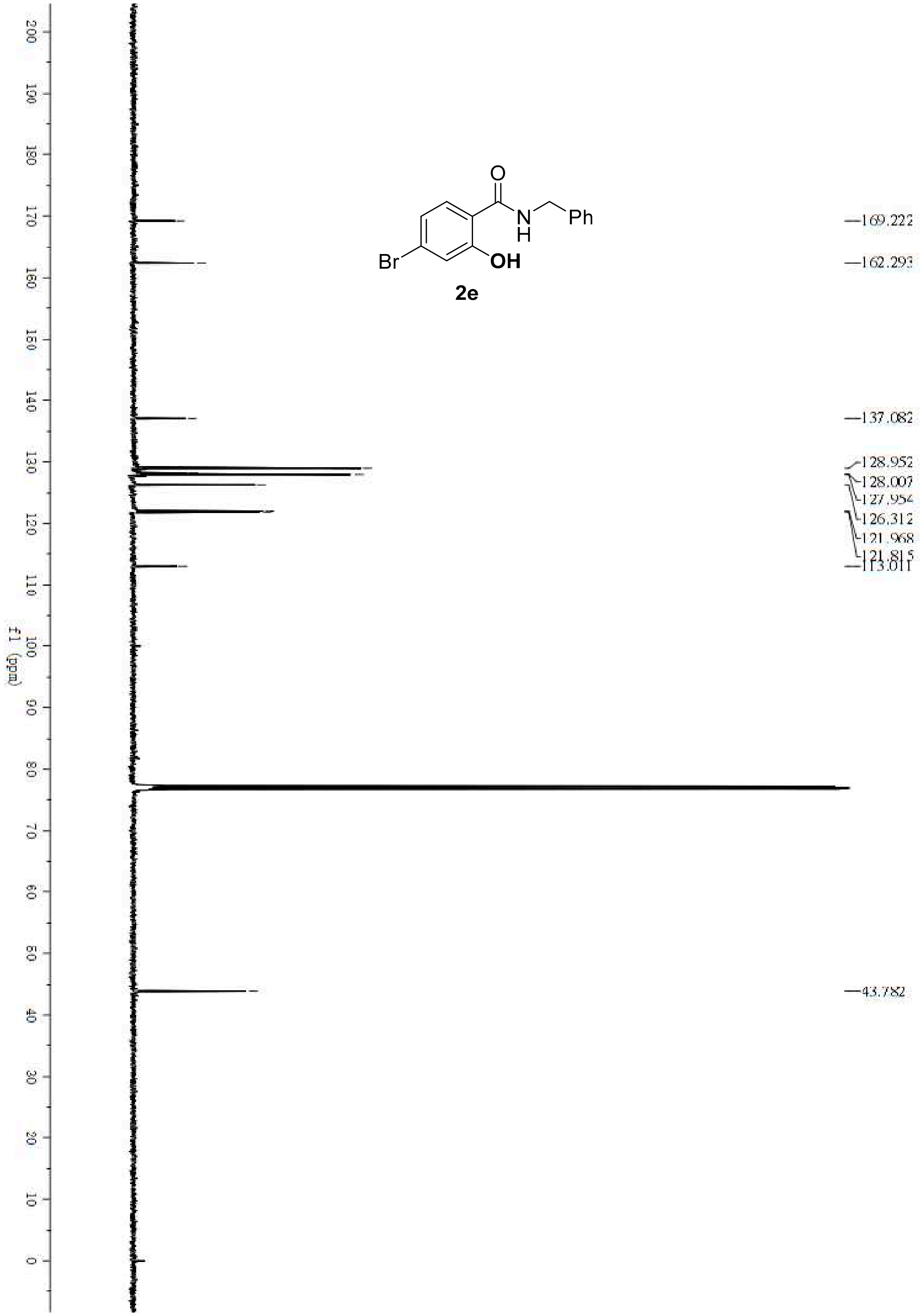

^{1}H NMR (400 MHz, $CDCl_3$) spectrum of **2f**

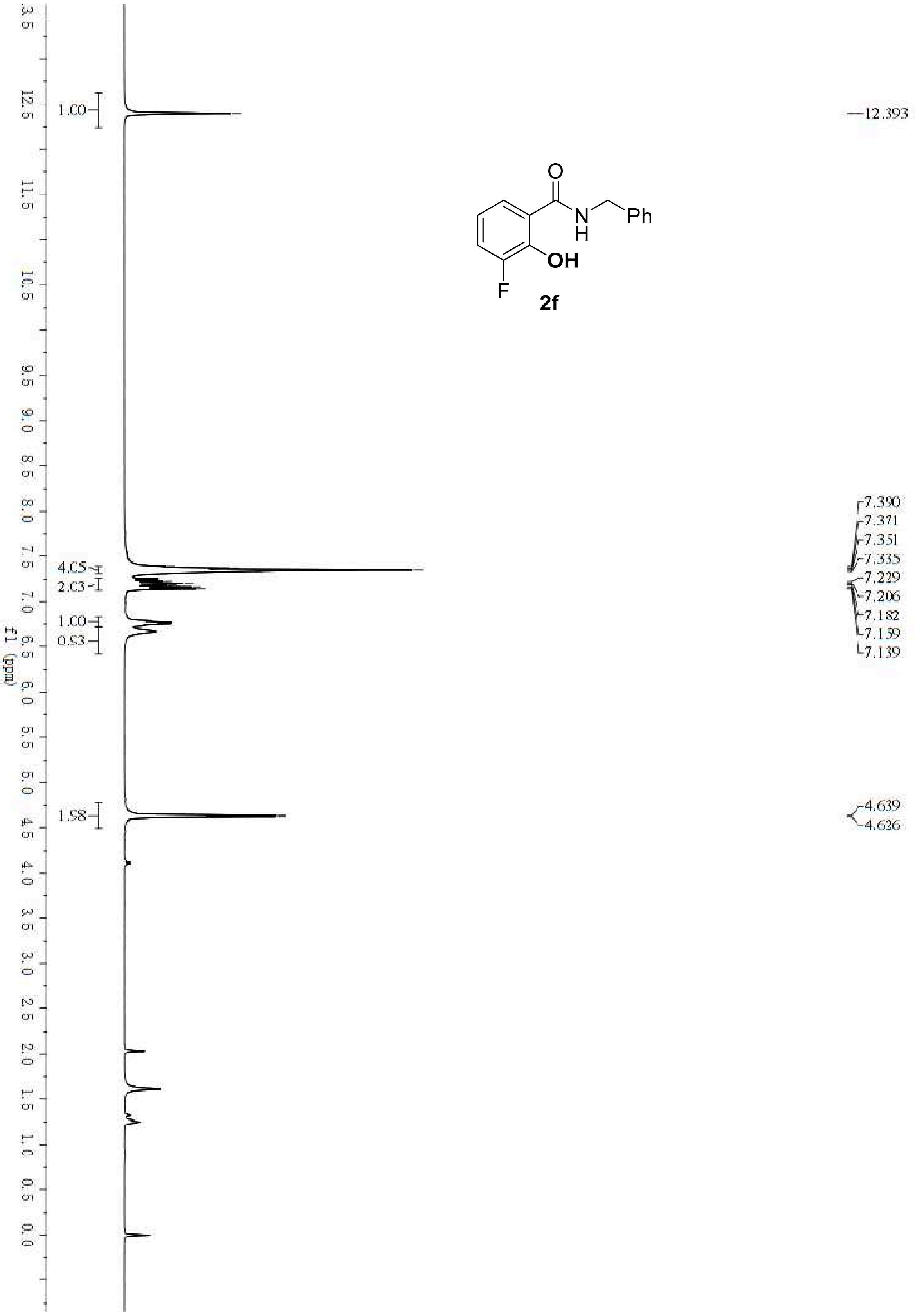

$^{13}C\ \{^{1}H\}$ NMR (100 MHz, $CDCl_3$) spectrum of **2f**

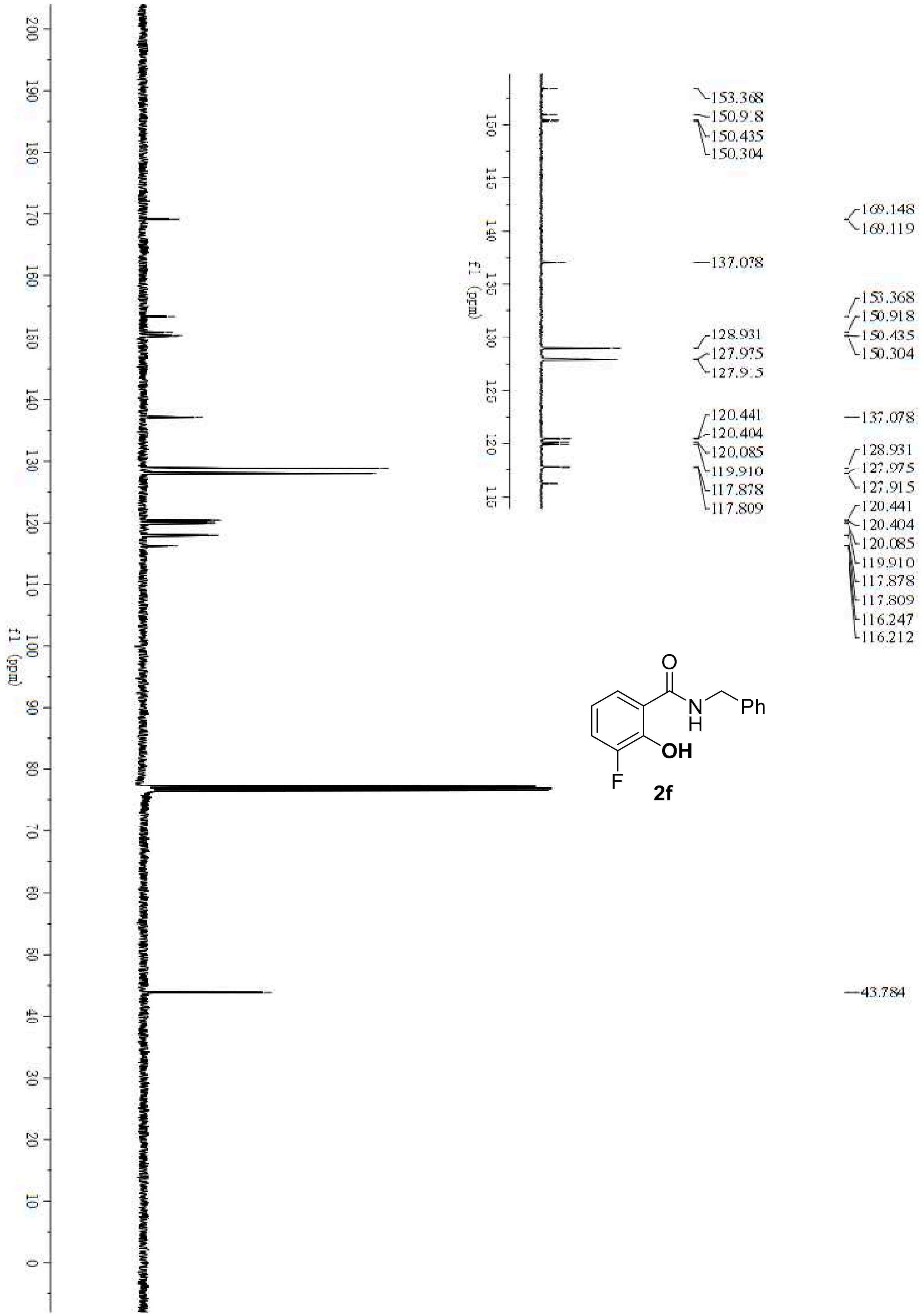

^{1}H NMR (400 MHz, $CDCl_3$) spectrum of **2g**

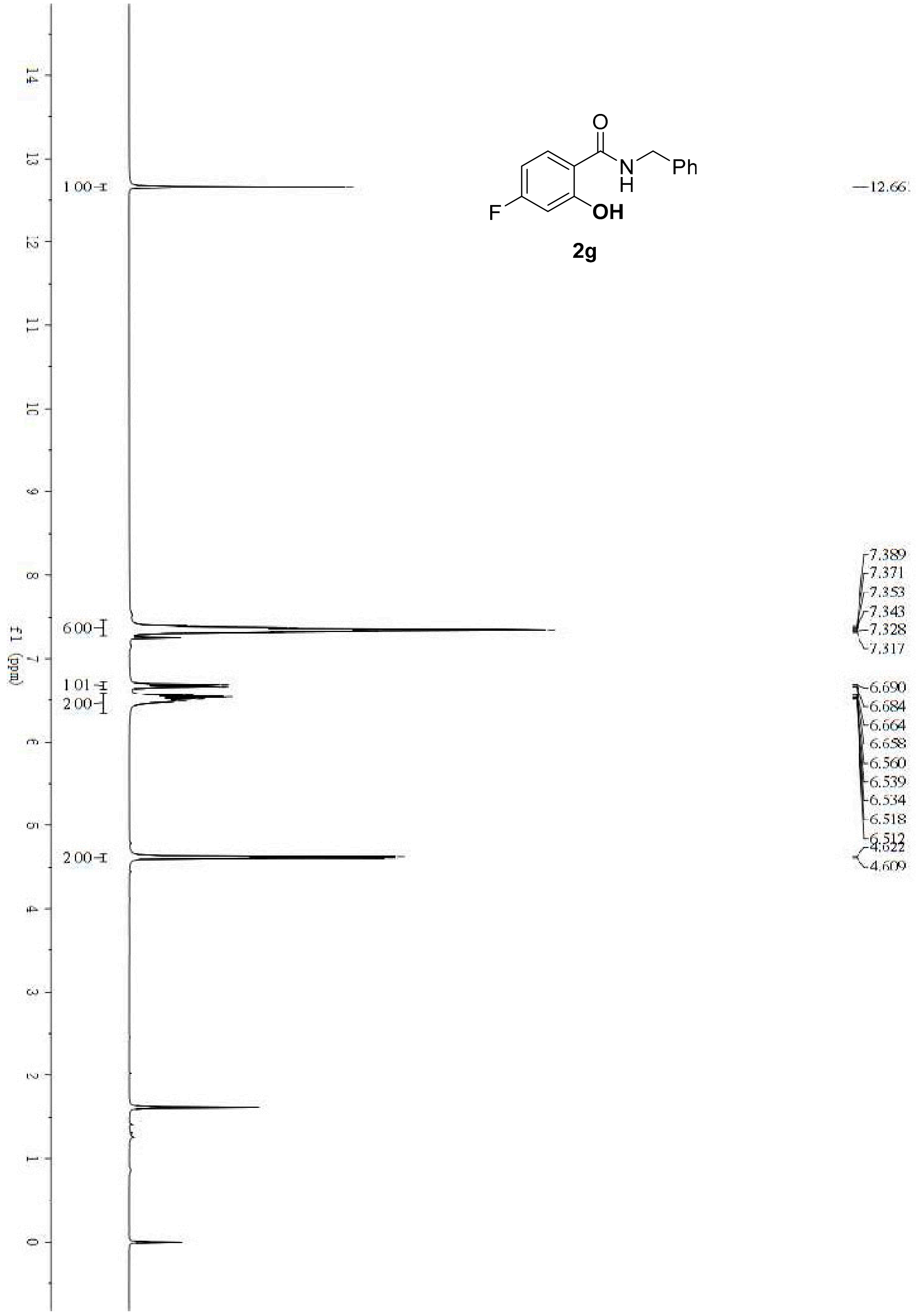

$^{13}C\ \{^{1}H\}$ NMR (100 MHz, $CDCl_3$) spectrum of **2g**

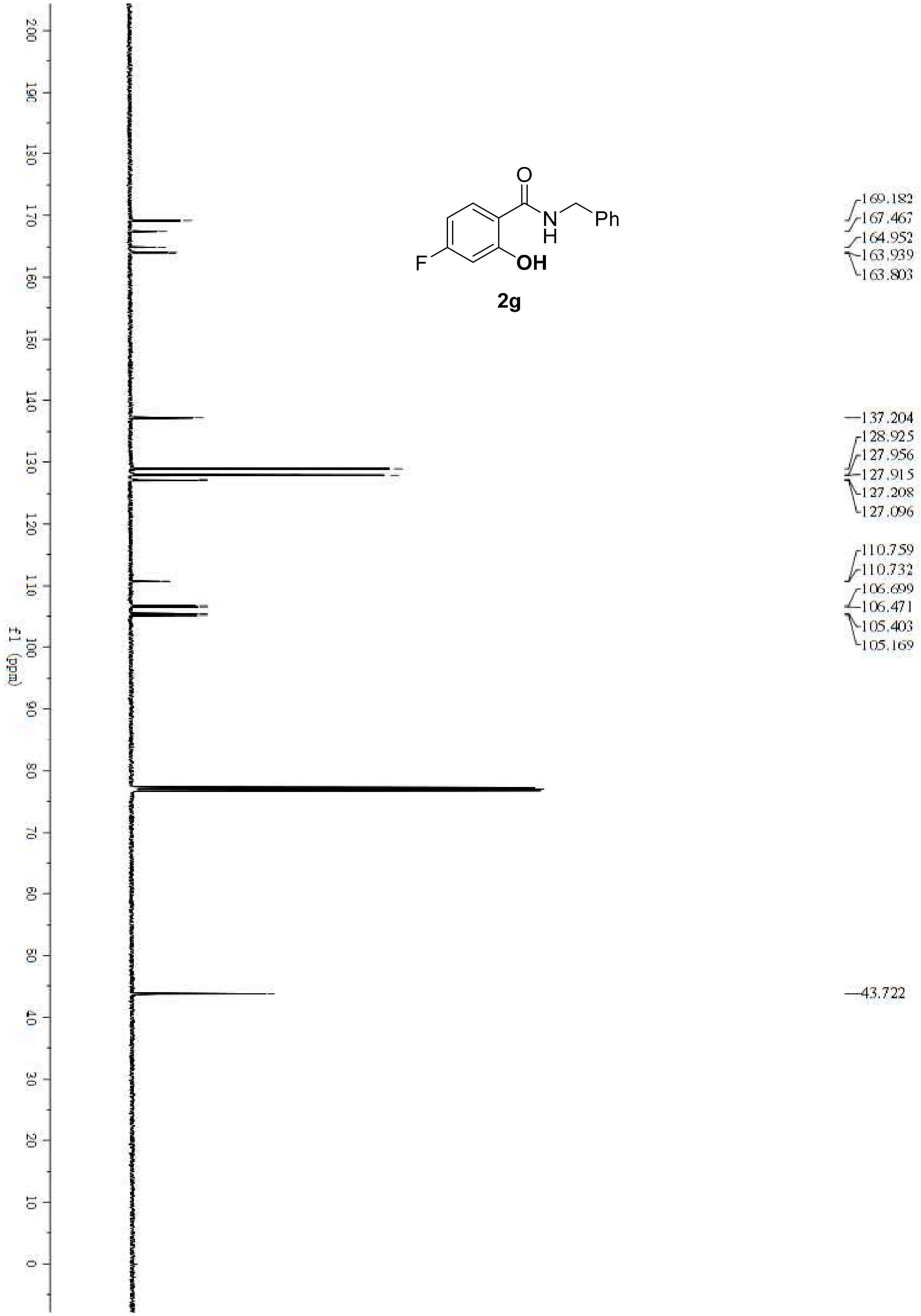

^{1}H NMR (400 MHz, $CDCl_3$) spectrum of **2h**

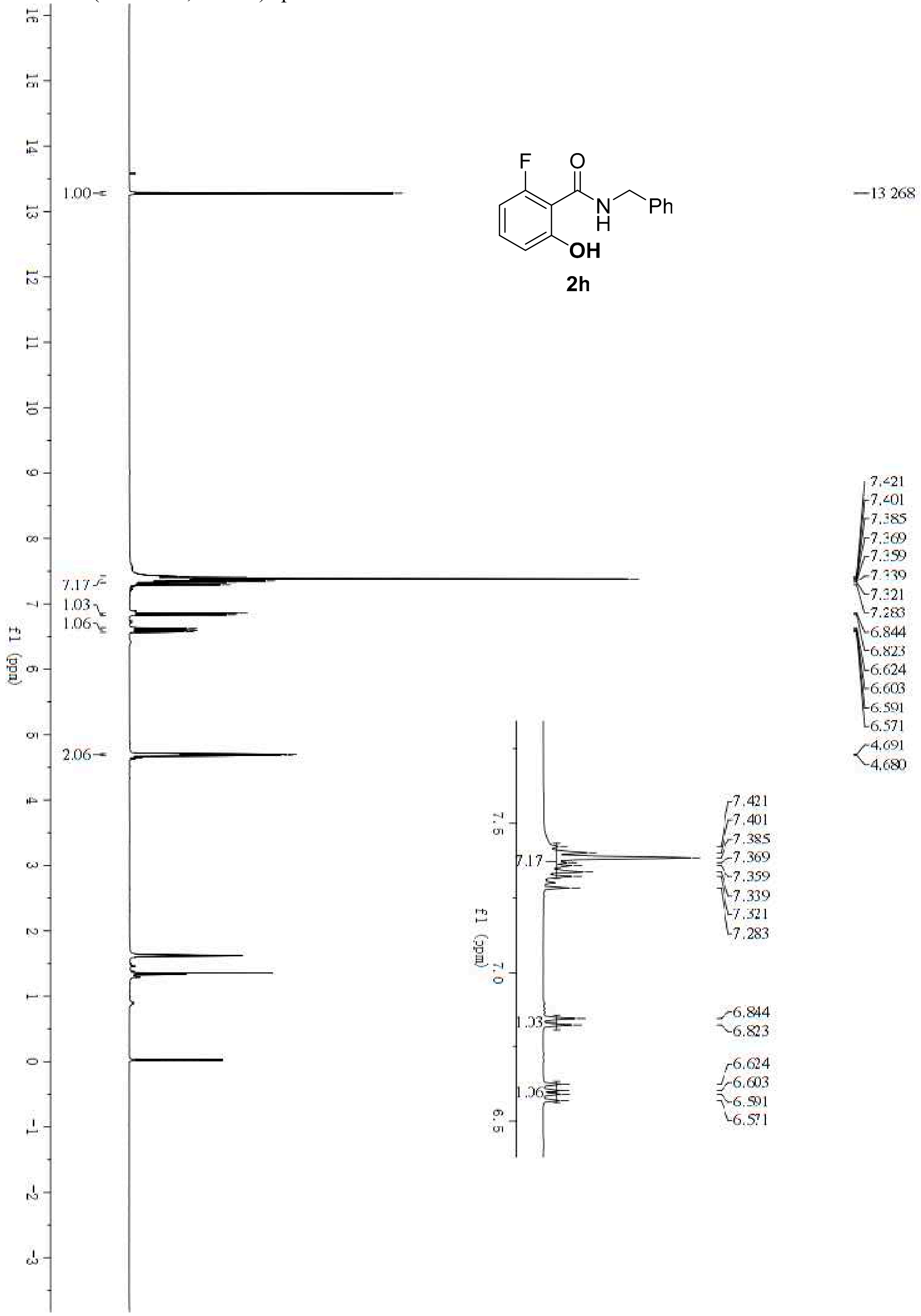

$^{13}C\ \{^{1}H\}$ NMR (100 MHz, $CDCl_3$) spectrum of **2h**

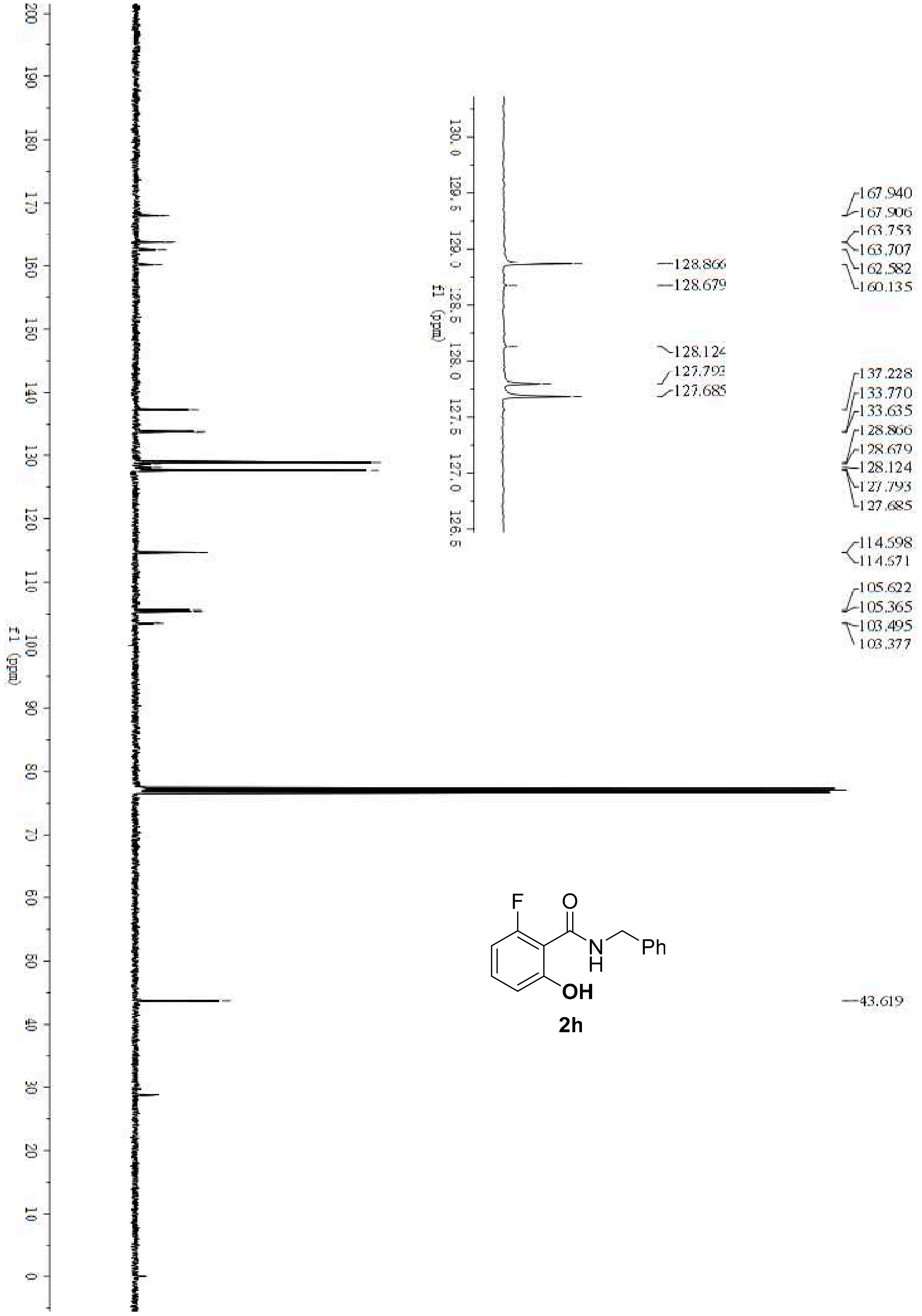

^{1}H NMR (400 MHz, $CDCl_3$) spectrum of **2i**

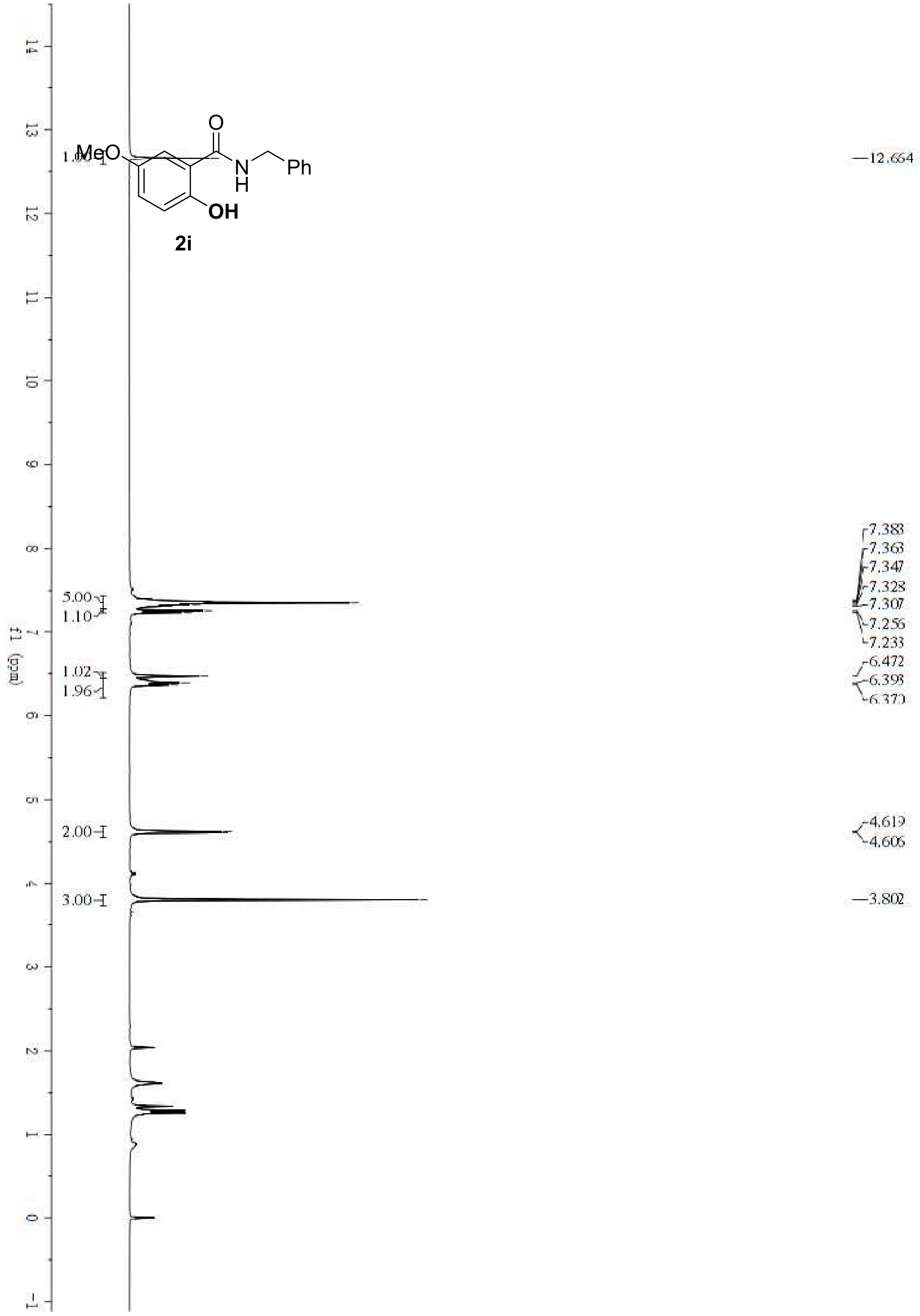

$^{13}C\ \{^1H\}$ NMR (100 MHz, $CDCl_3$) spectrum of **2i**

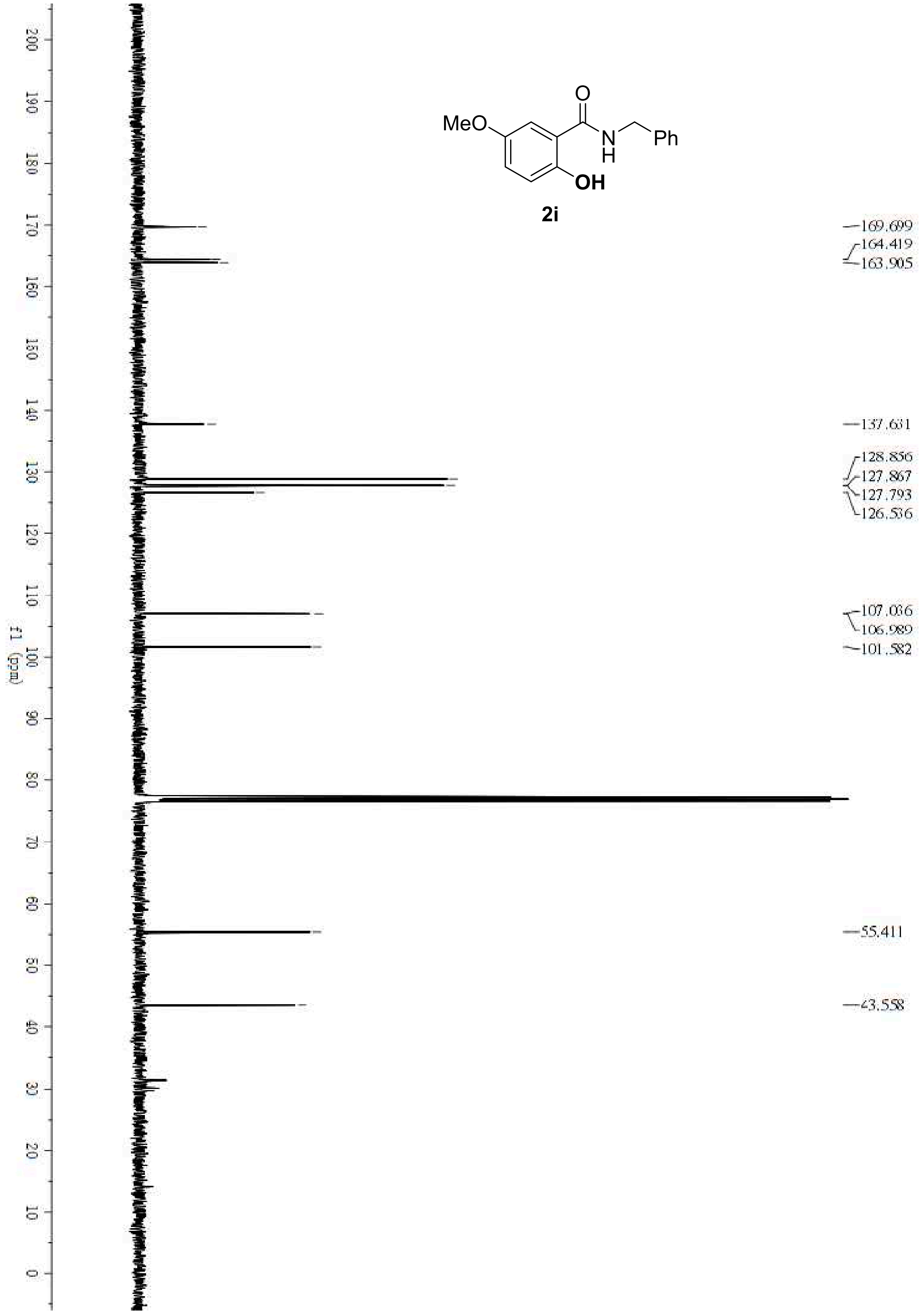

^{1}H NMR (400 MHz, $CDCl_3$) spectrum of **2j**

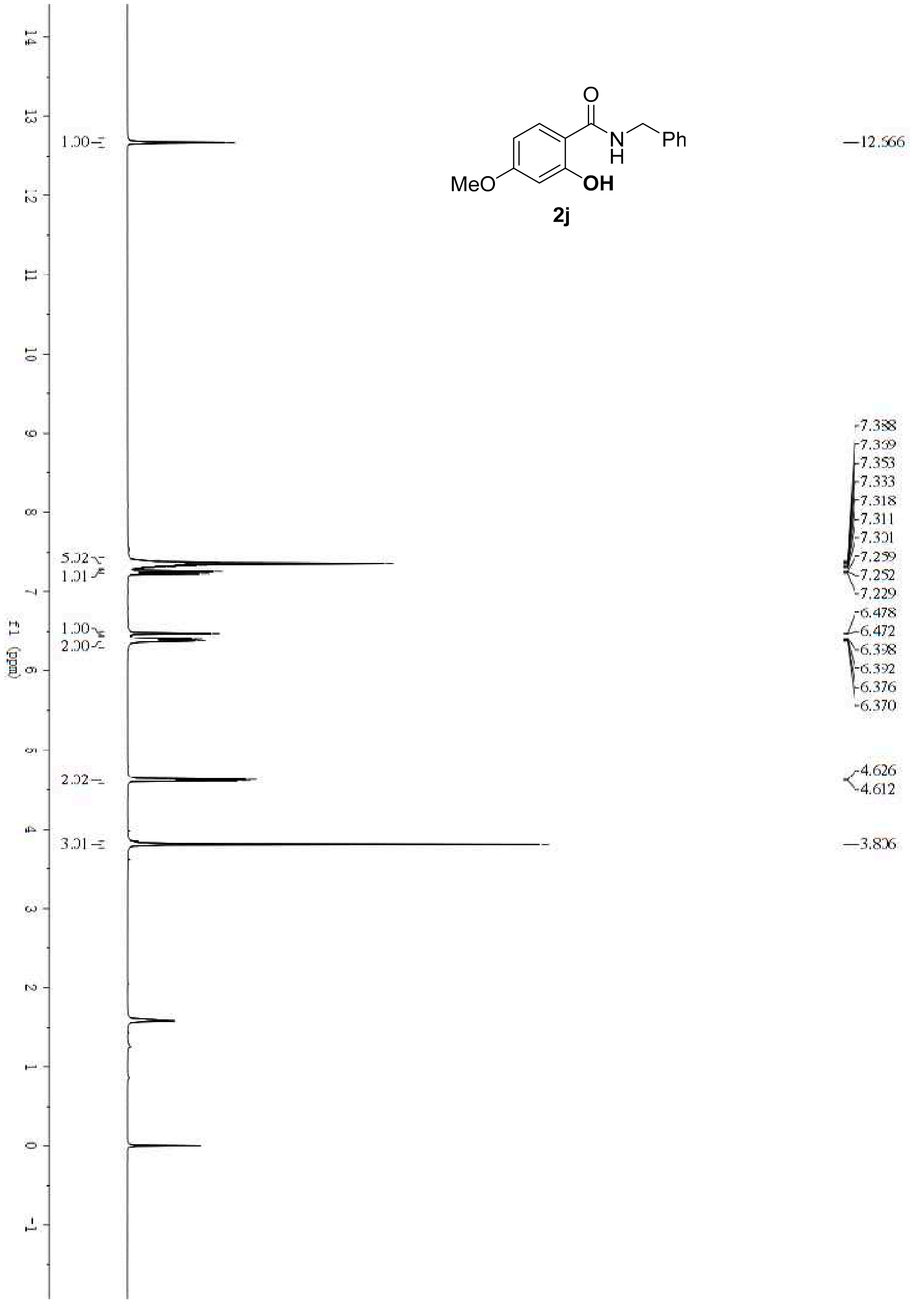

^{13}C {^{1}H} NMR (100 MHz, $CDCl_3$) spectrum of **2j**

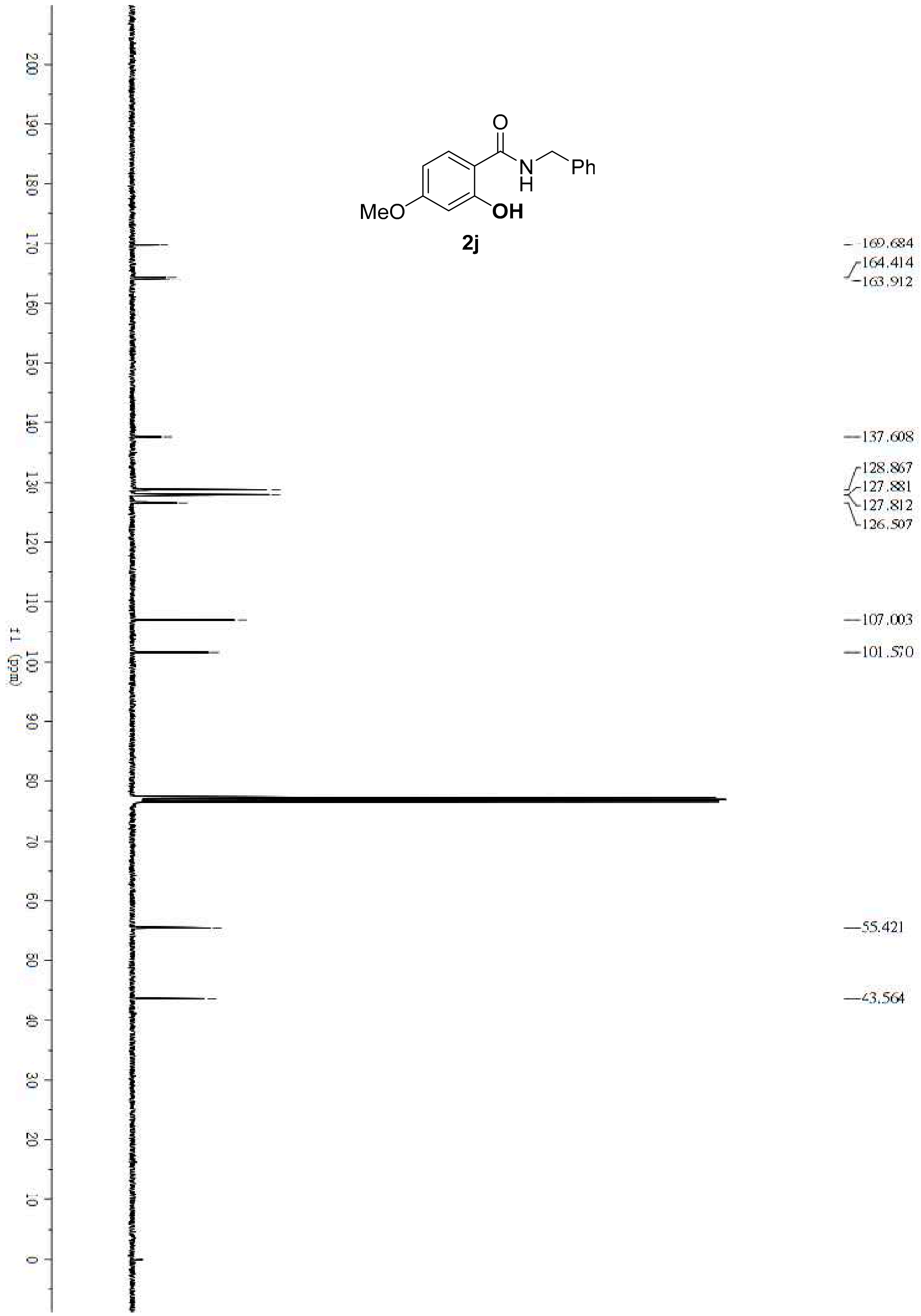

^{1}H NMR (400 MHz, $CDCl_3$) spectrum of **2k**

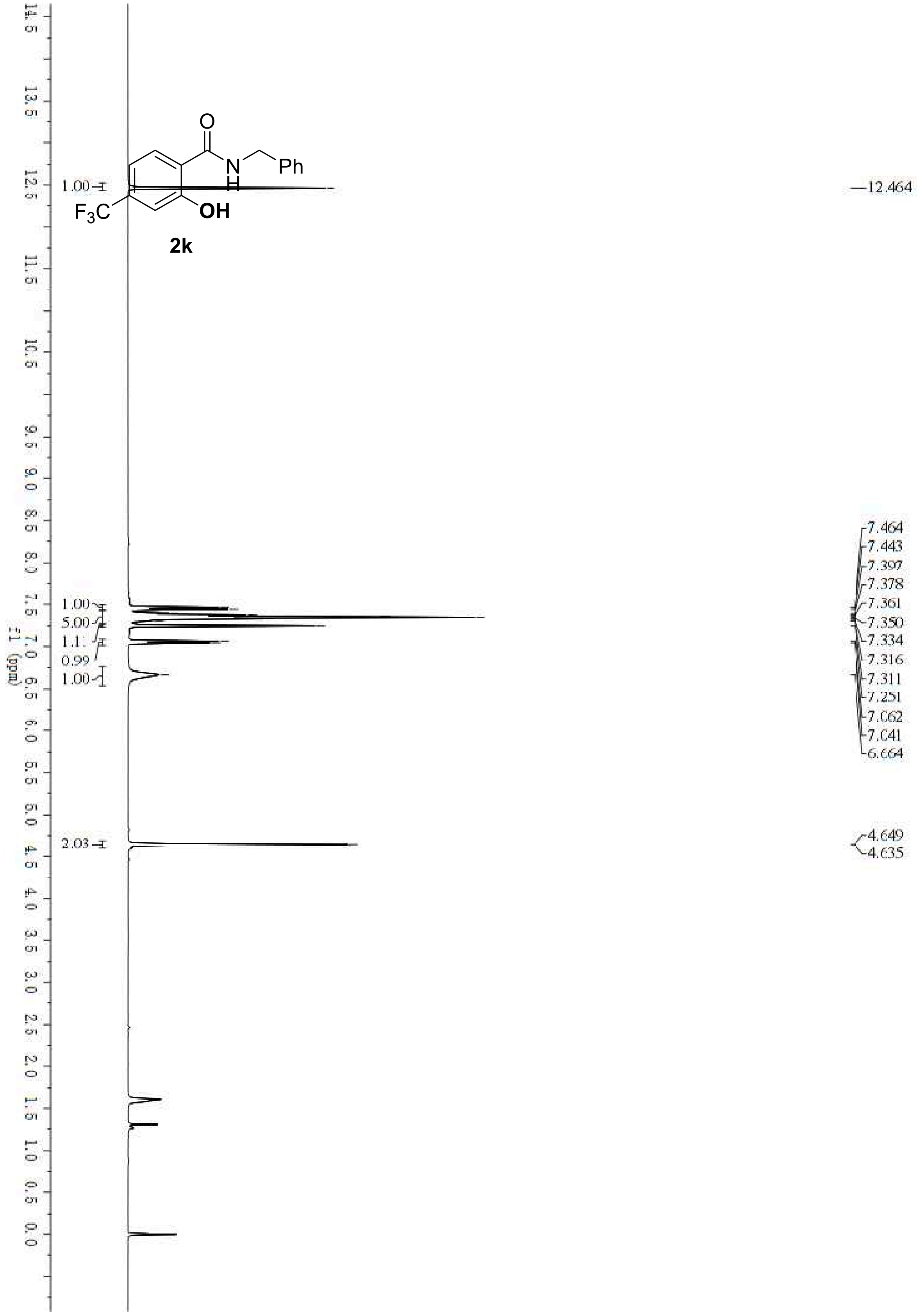

^{13}C {^{1}H} NMR (100 MHz, $CDCl_3$) spectrum of **2k**

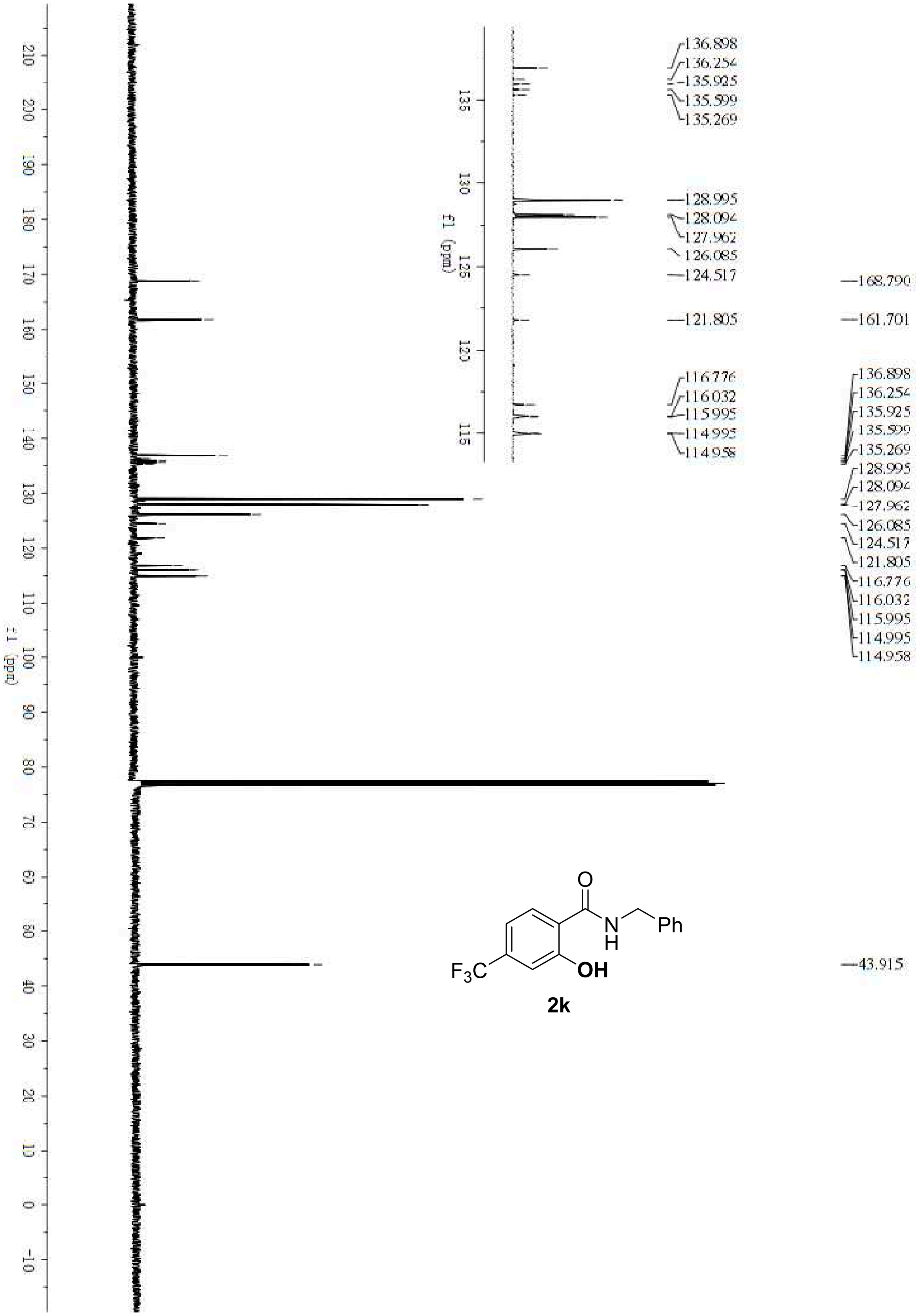

^{1}H NMR (400 MHz, $CDCl_3$) spectrum of **2l**

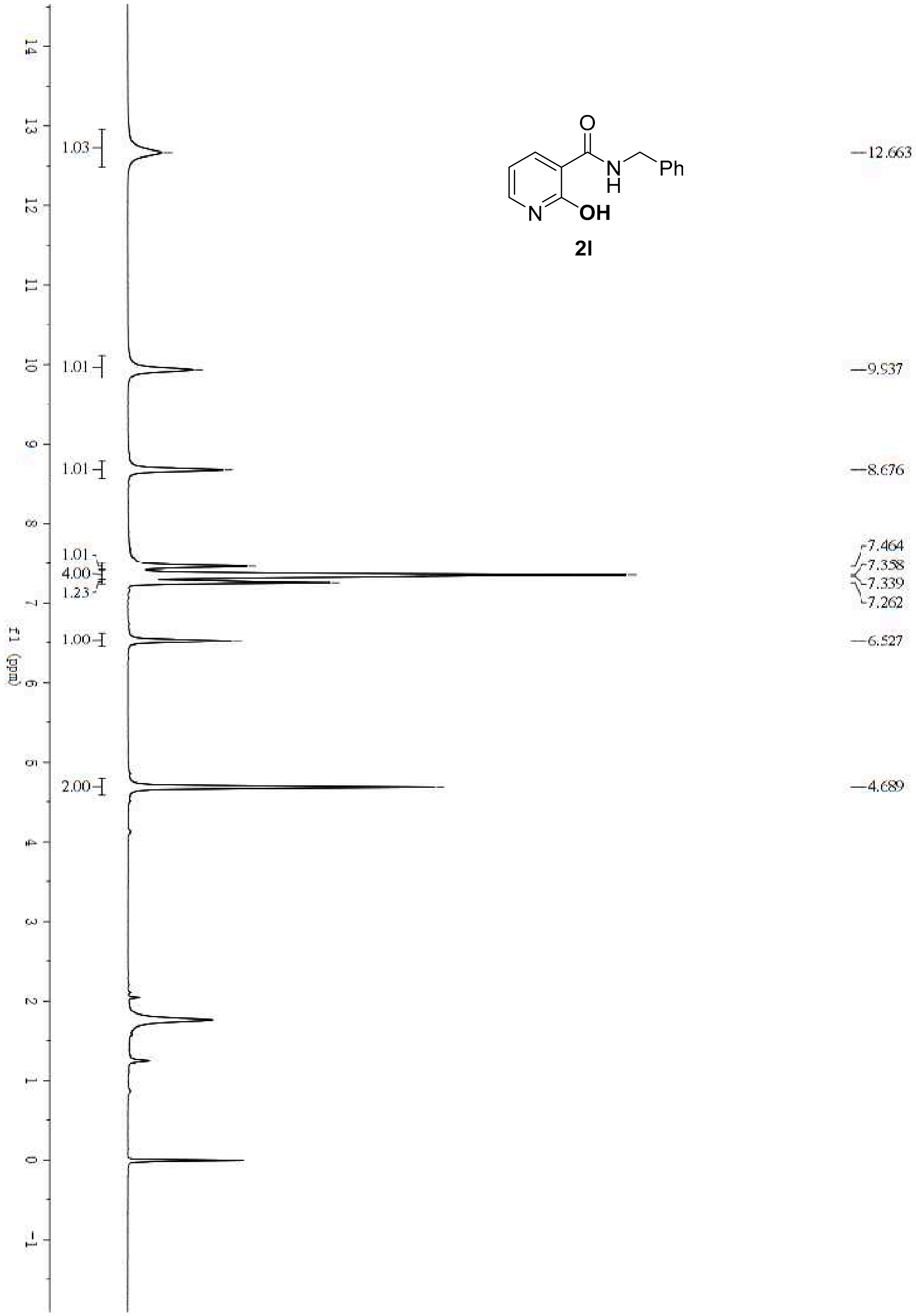

$^{13}C\ \{^{1}H\}$ NMR (100 MHz, $CDCl_3$) spectrum of **2l**

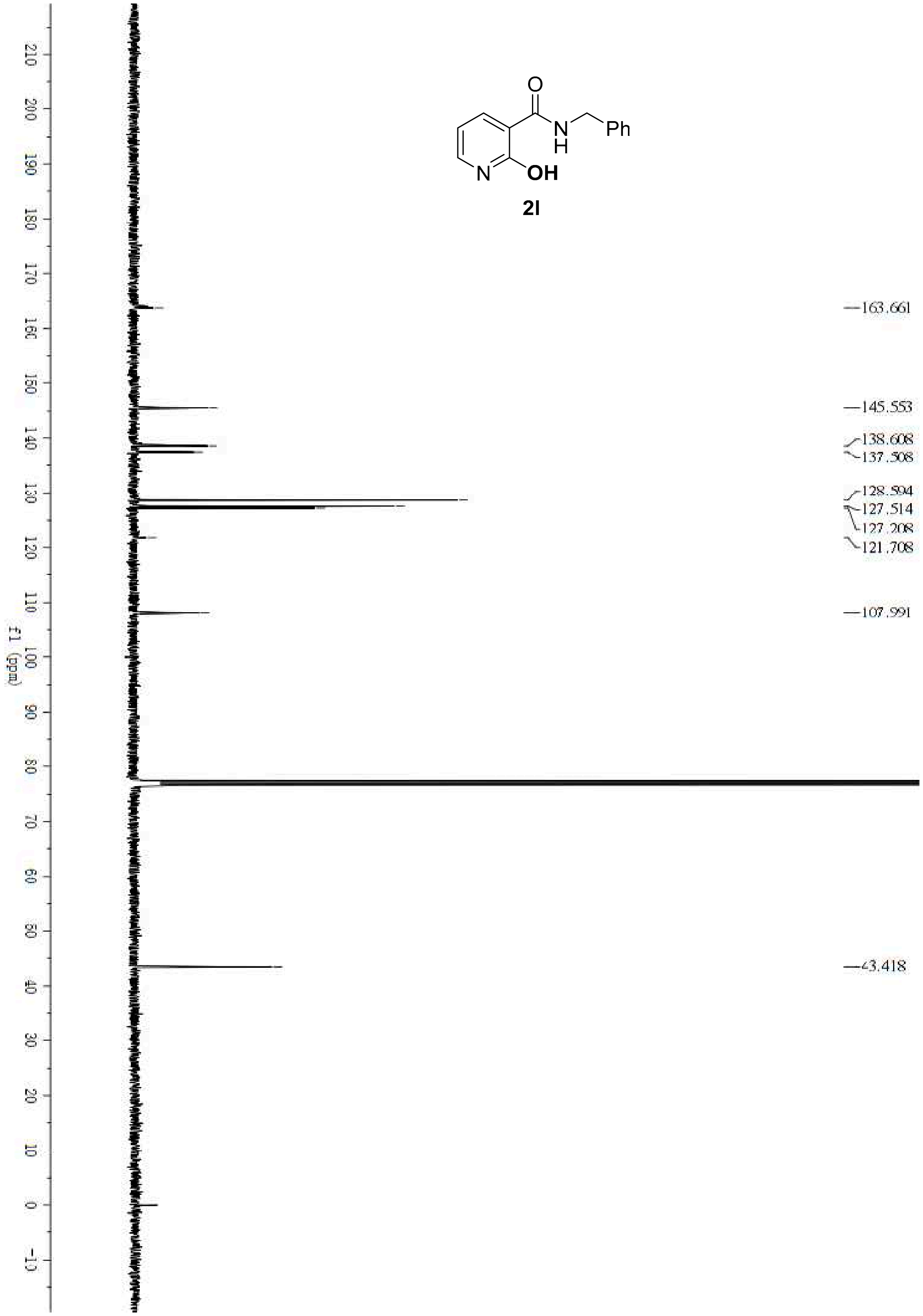

^{1}H NMR (400 MHz, $CDCl_3$) spectrum of **2m**

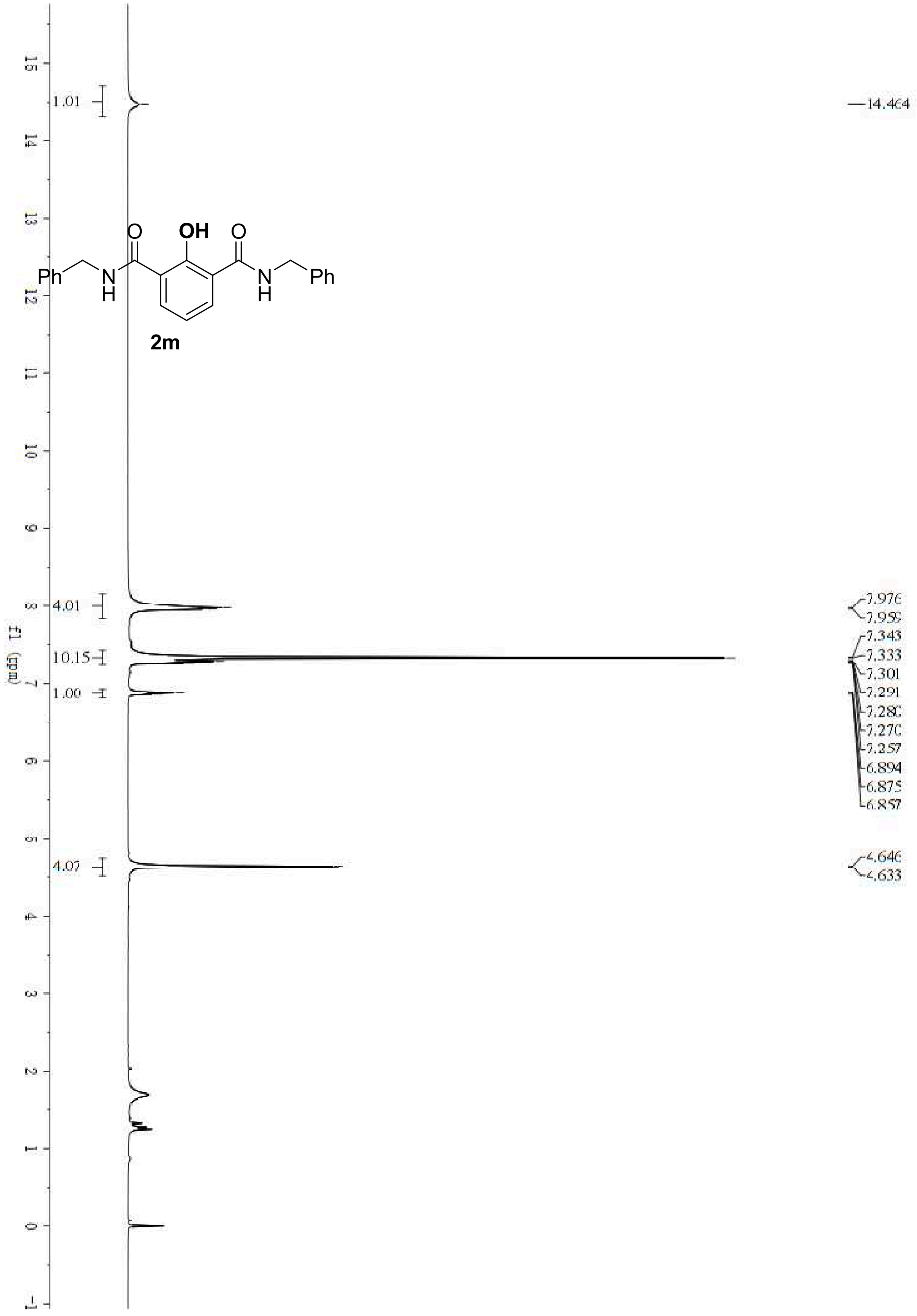

$^{13}C\ \{^1H\}$ NMR (100 MHz, $CDCl_3$) spectrum of **2m**

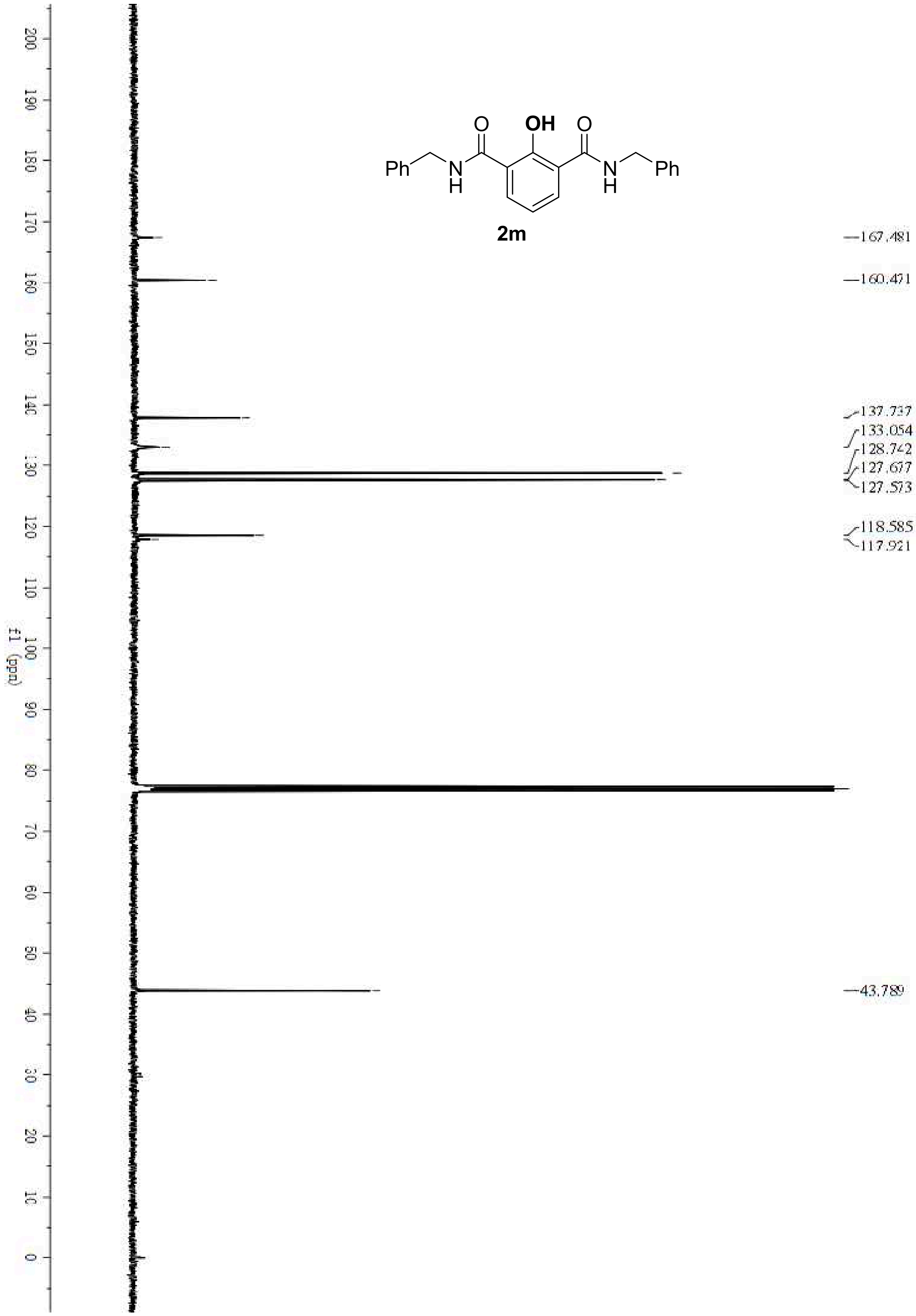

^{1}H NMR (400 MHz, $CDCl_3$) spectrum of **2n**

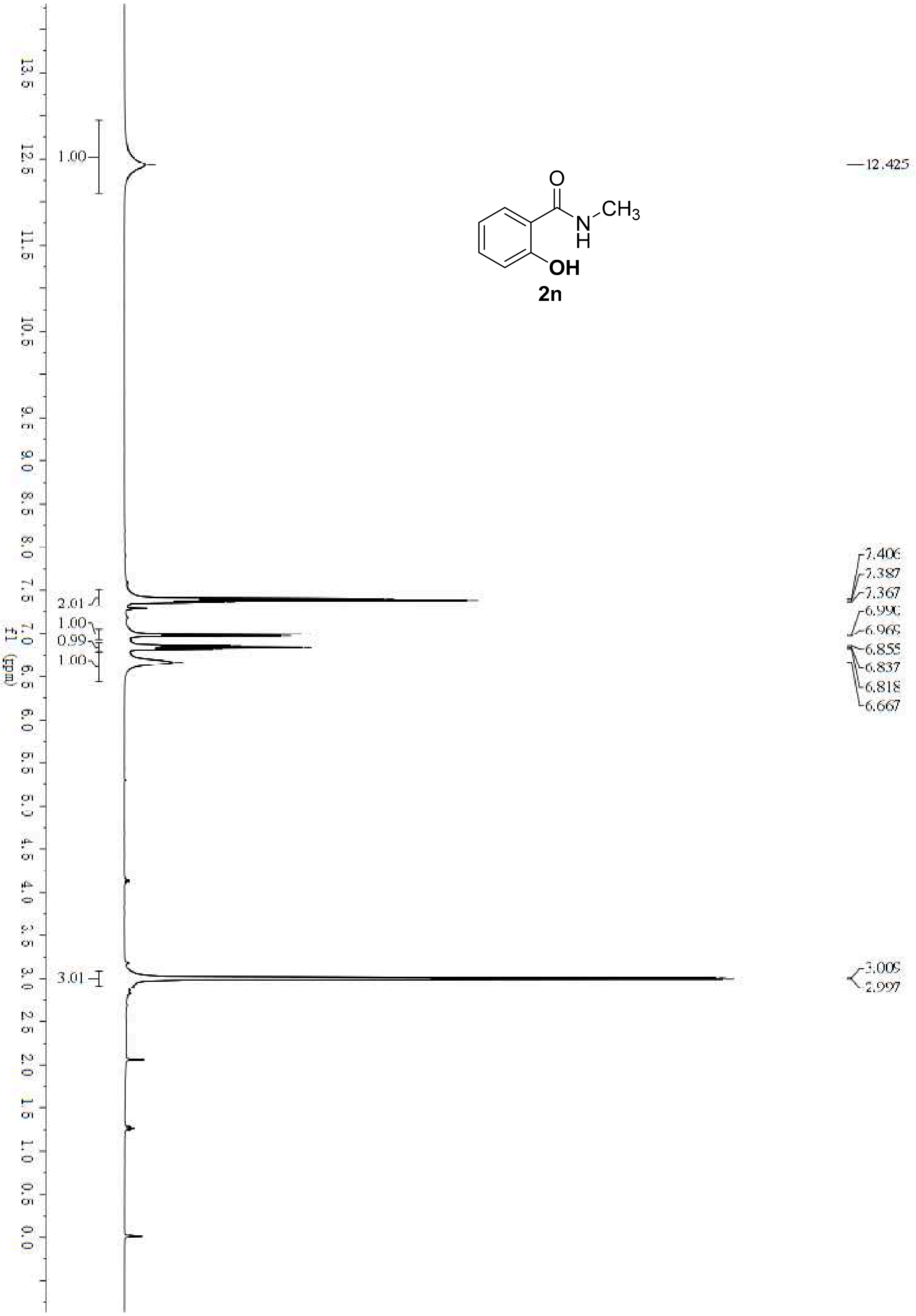

^{13}C {^1H} NMR (100 MHz, $CDCl_3$) spectrum of **2n**

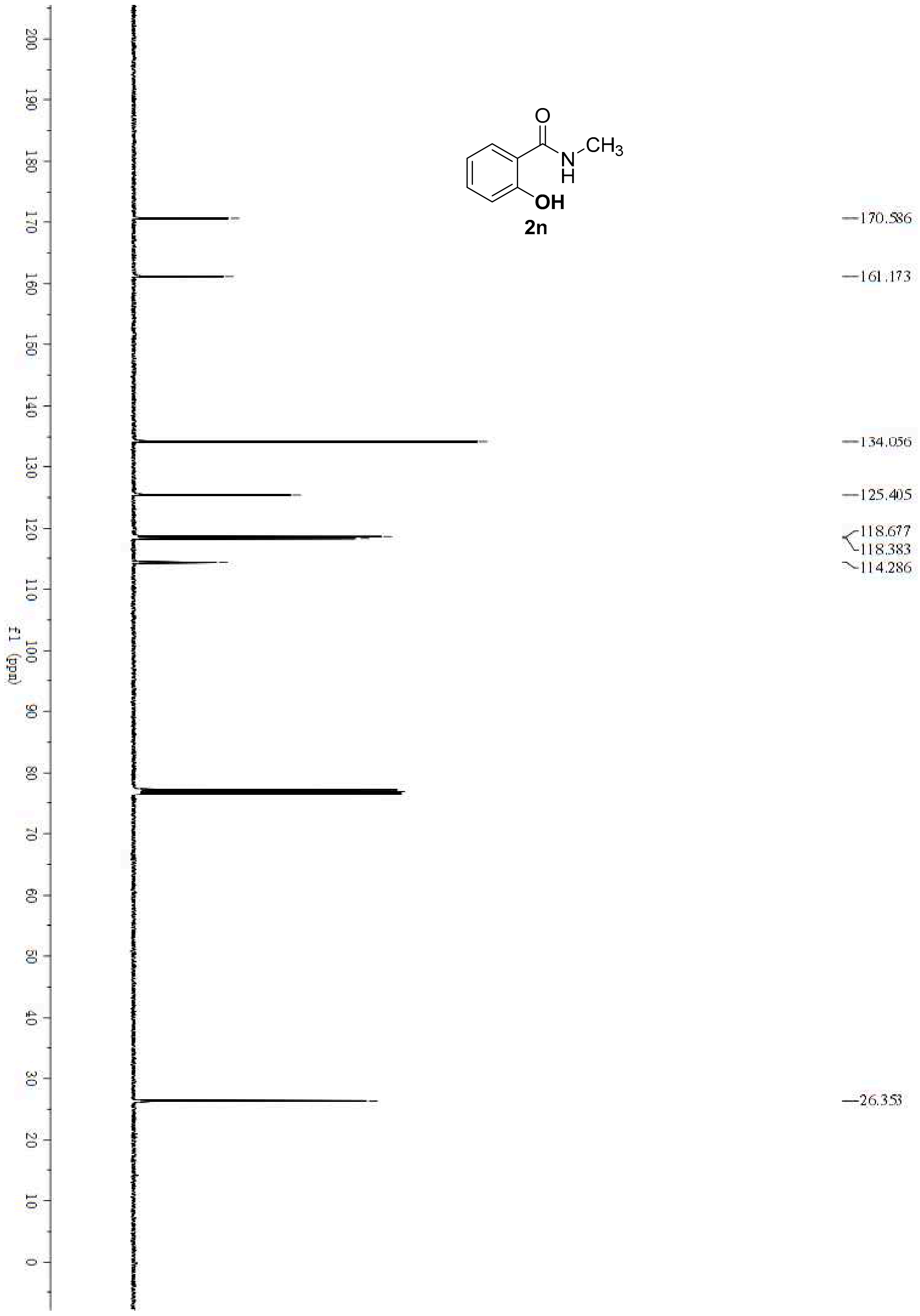

^{1}H NMR (400 MHz, $CDCl_3$) spectrum of **2o**

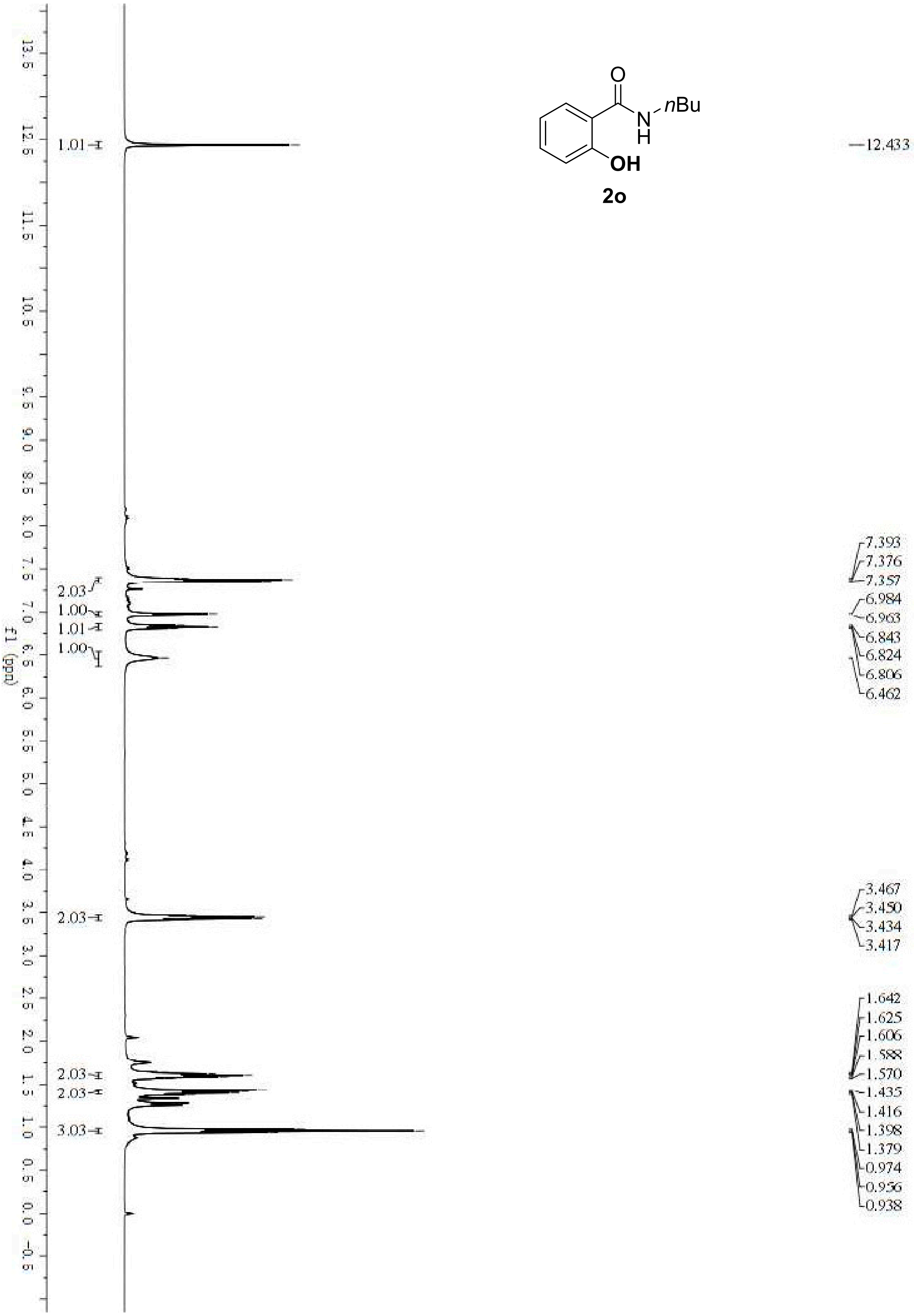

^{13}C {^{1}H} NMR (100 MHz, $CDCl_3$) spectrum of **2o**

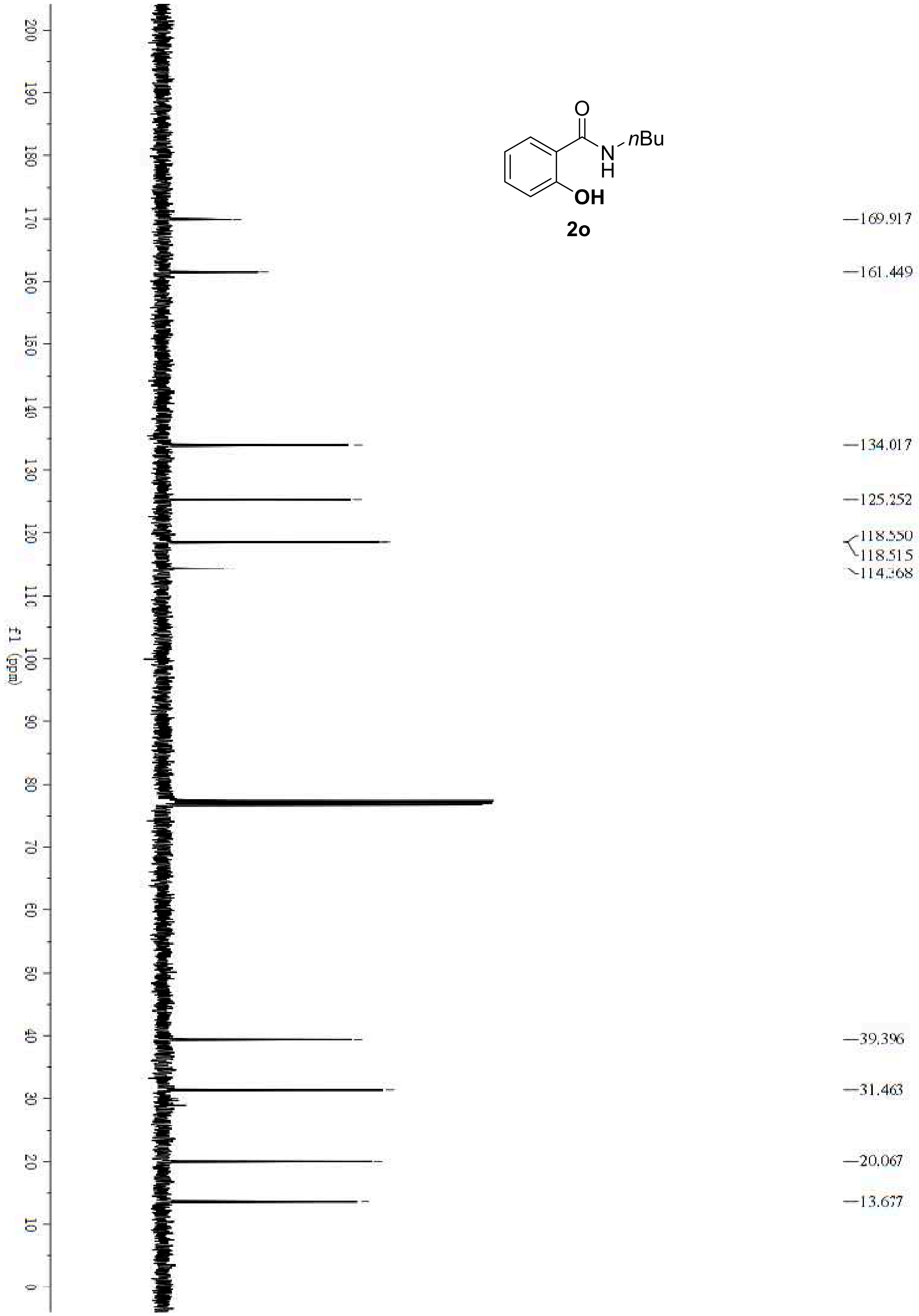

^{1}H NMR (400 MHz, $CDCl_3$) spectrum of **2p**

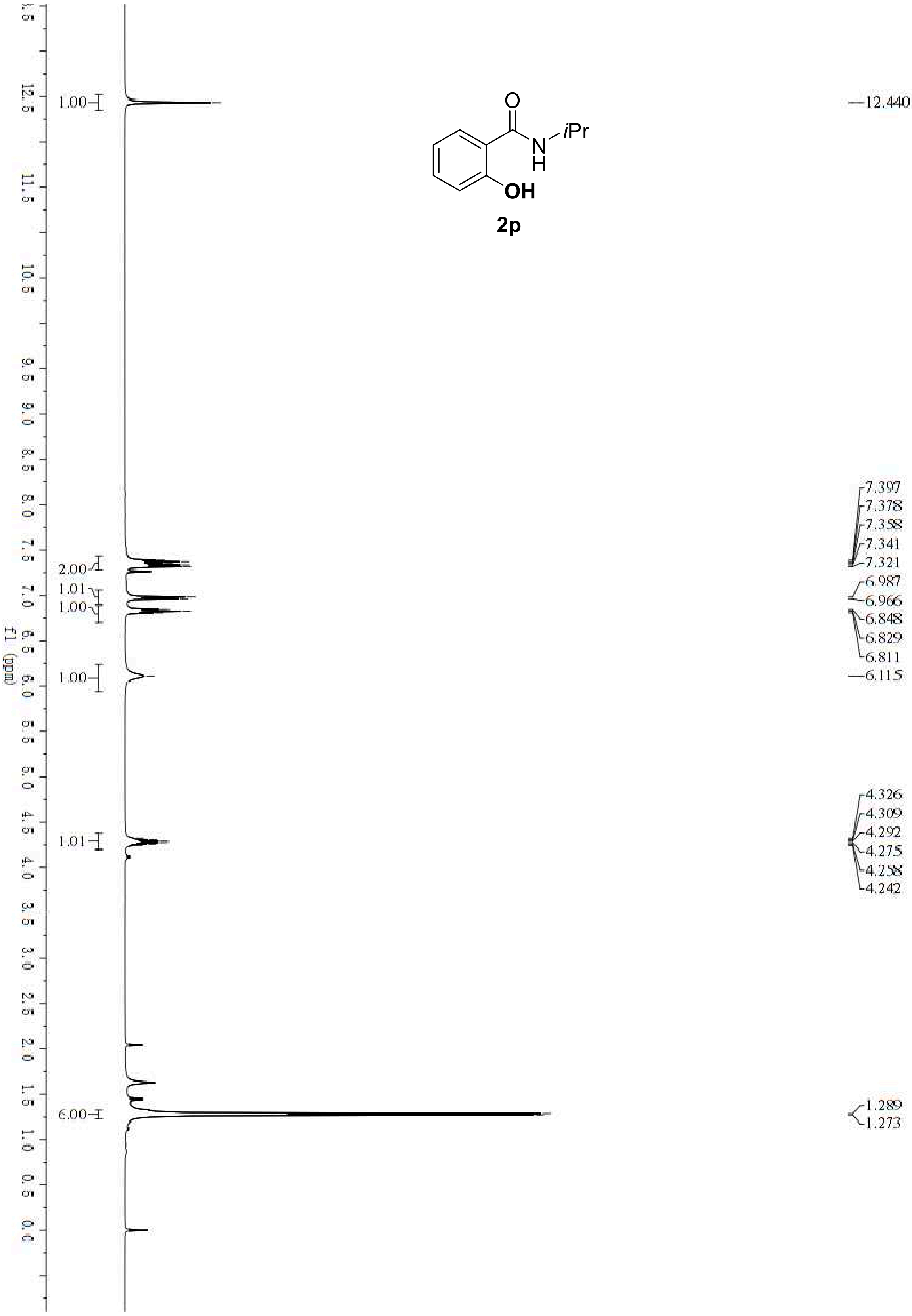

^{13}C {^{1}H} NMR (100 MHz, $CDCl_3$) spectrum of **2p**

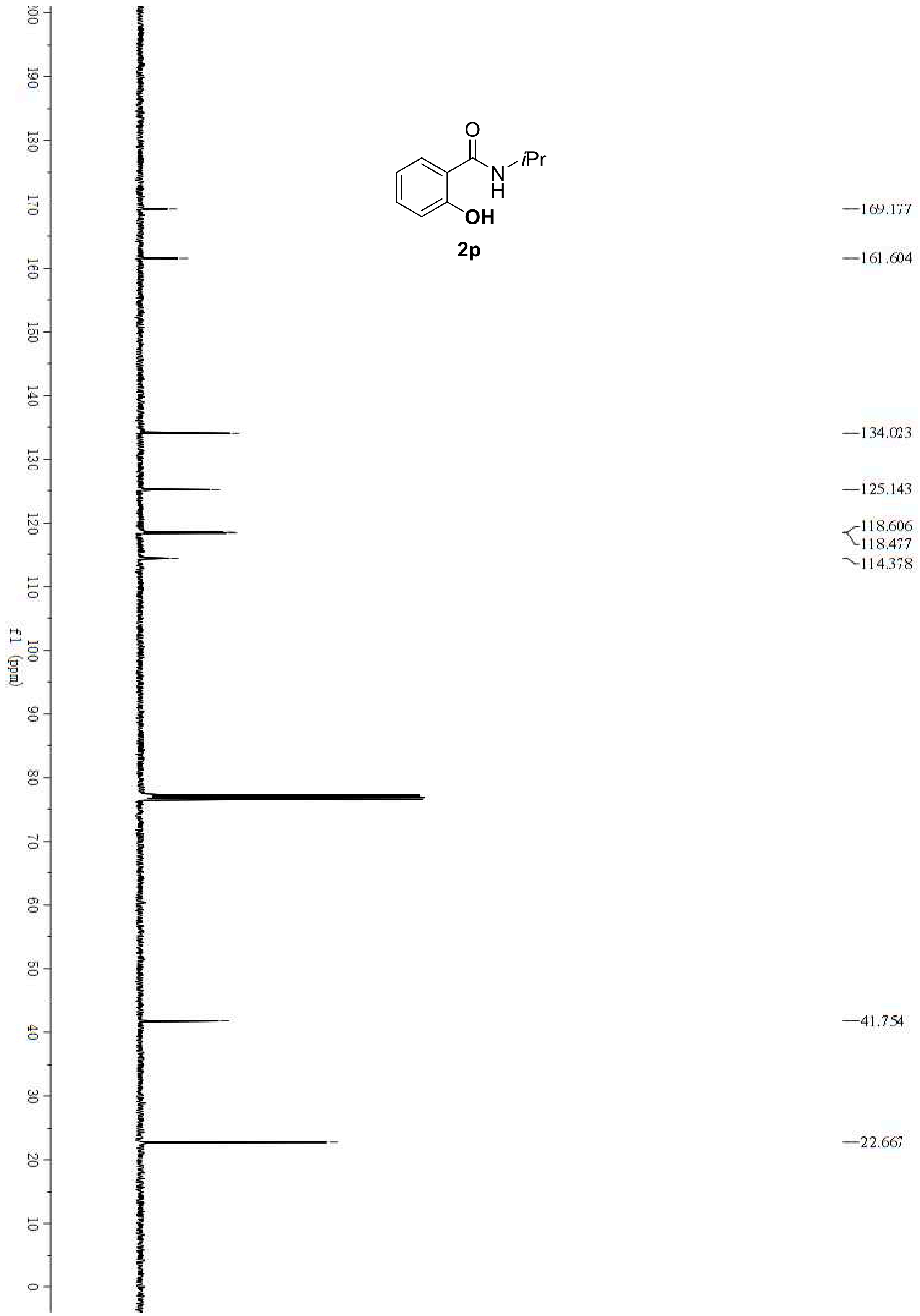

^{1}H NMR (400 MHz, $CDCl_3$) spectrum of **2q**

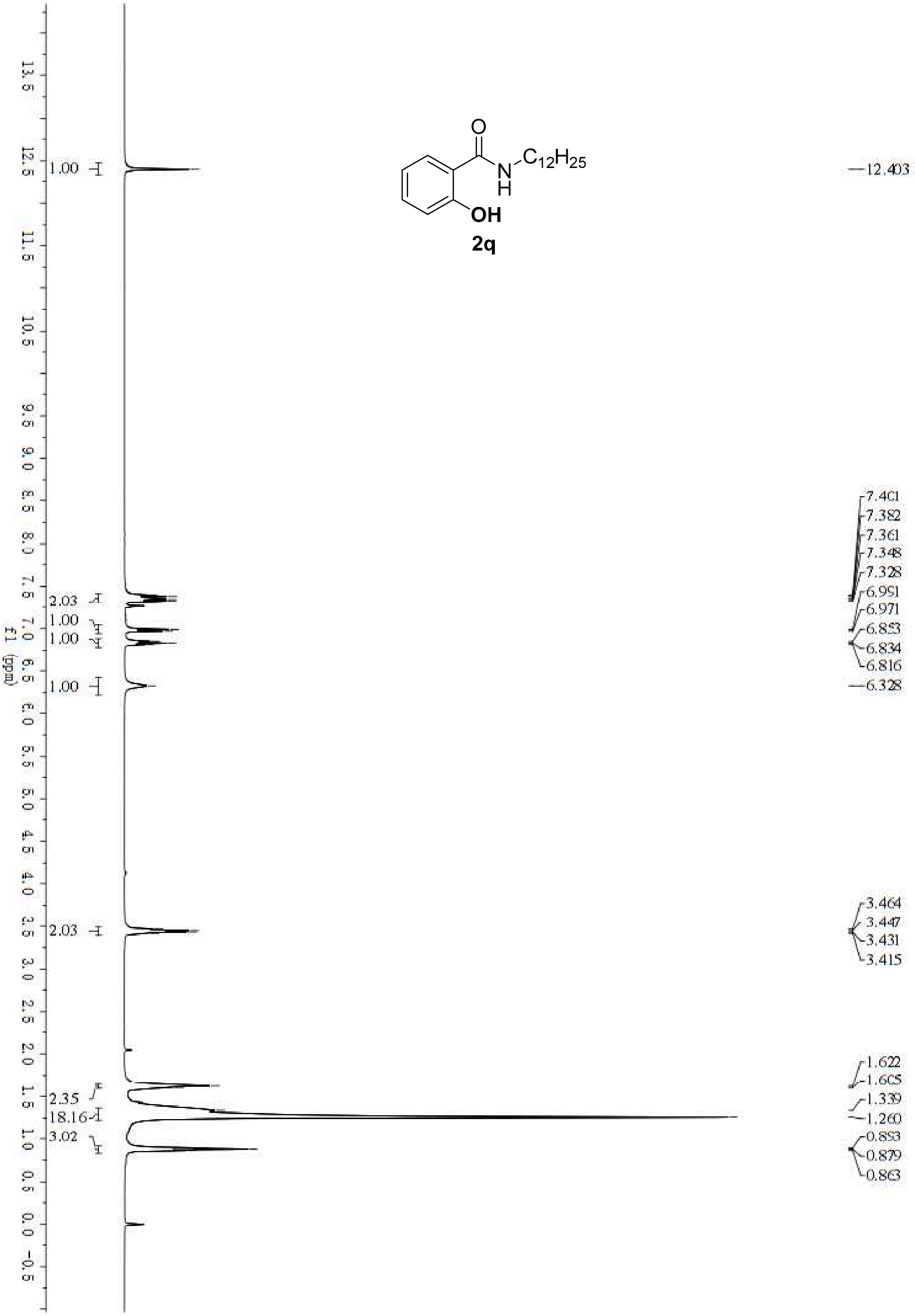

^{13}C {^{1}H} NMR (100 MHz, $CDCl_3$) spectrum of **2q**

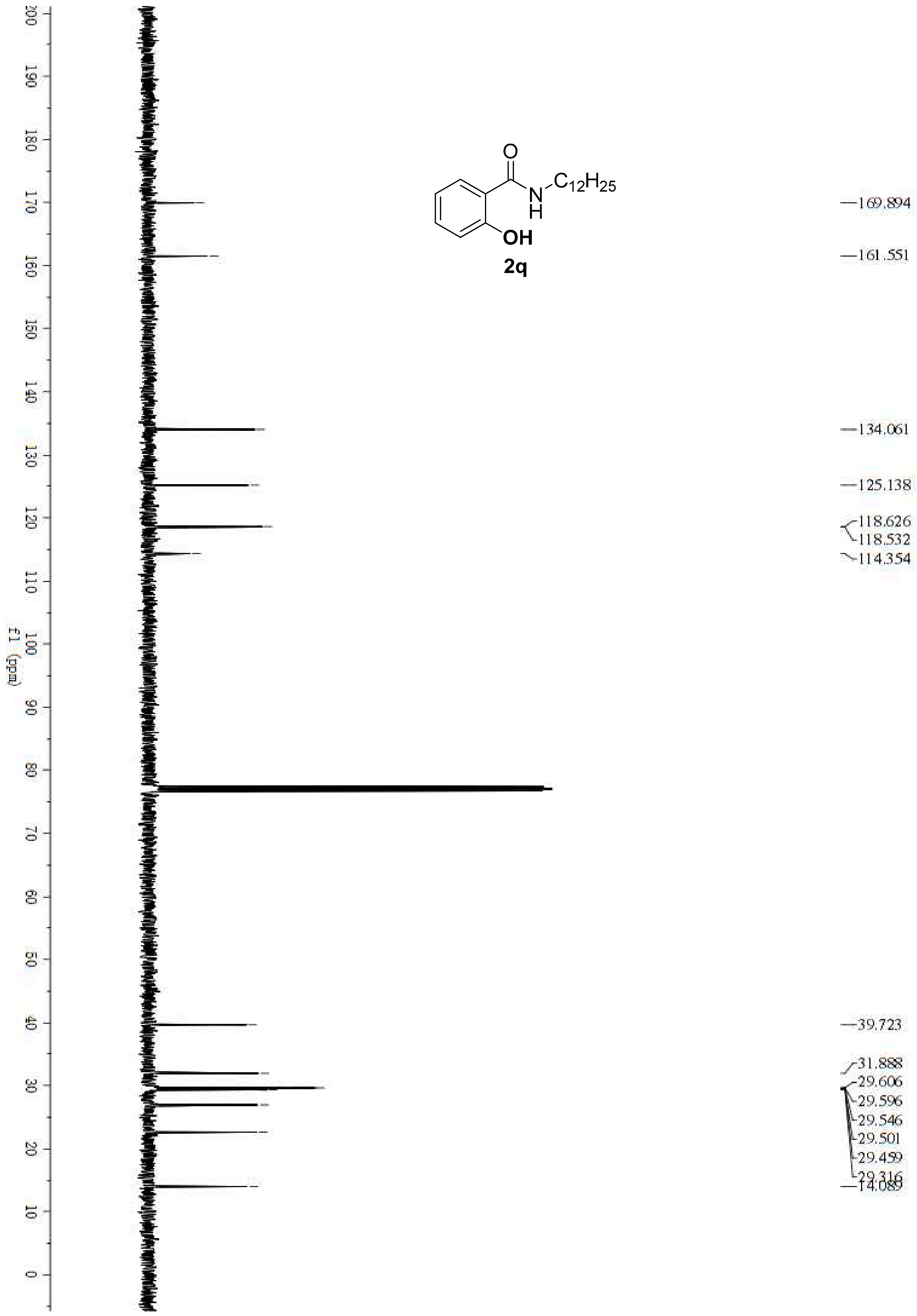

^{1}H NMR (400 MHz, $CDCl_3$) spectrum of **2r**

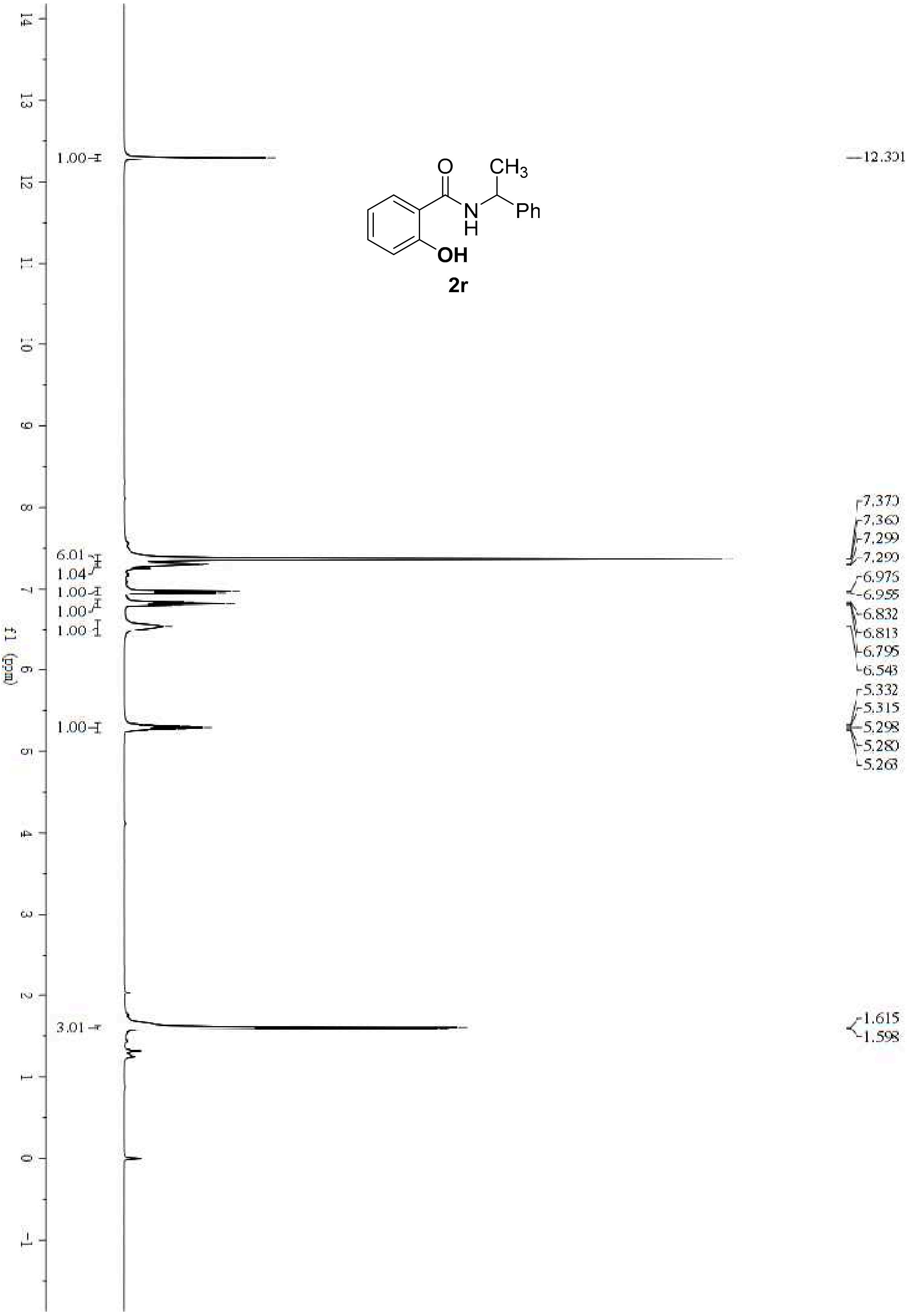

$^{13}C\ \{^{1}H\}$ NMR (100 MHz, $CDCl_3$) spectrum of **2r**

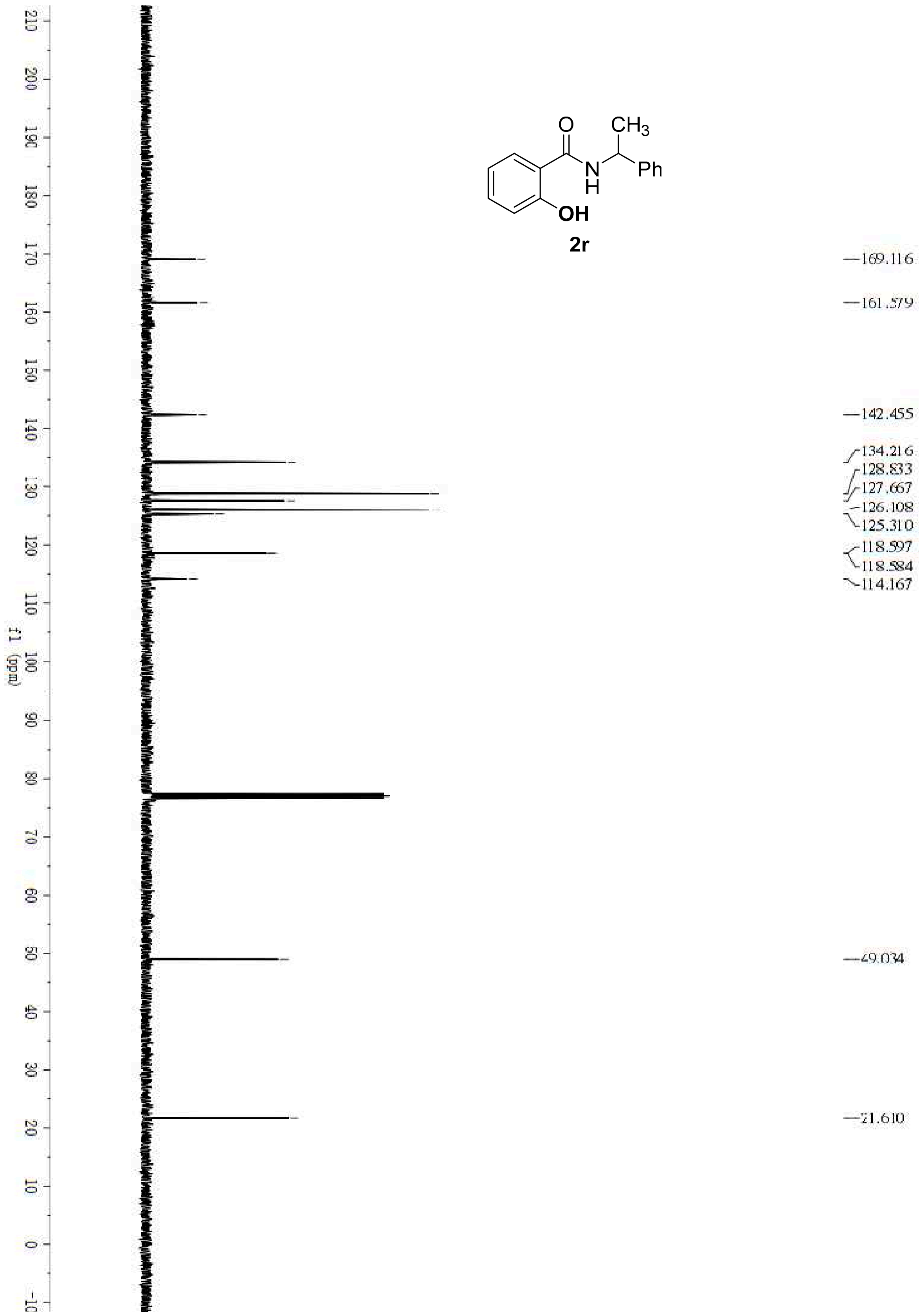

^{1}H NMR (400 MHz, $CDCl_3$) spectrum of **2s**

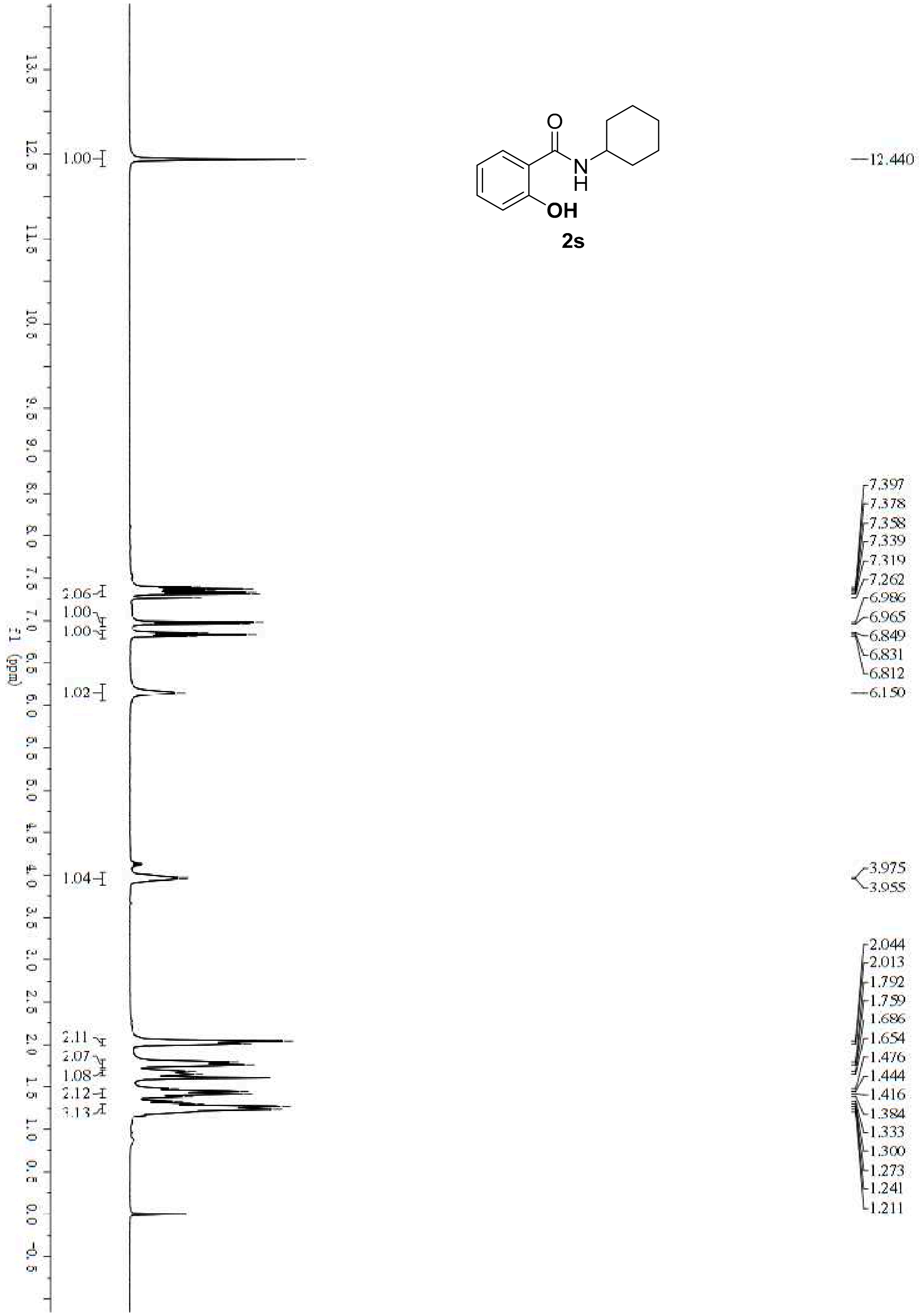

^{13}C {^{1}H} NMR (100 MHz, $CDCl_3$) spectrum of **2s**

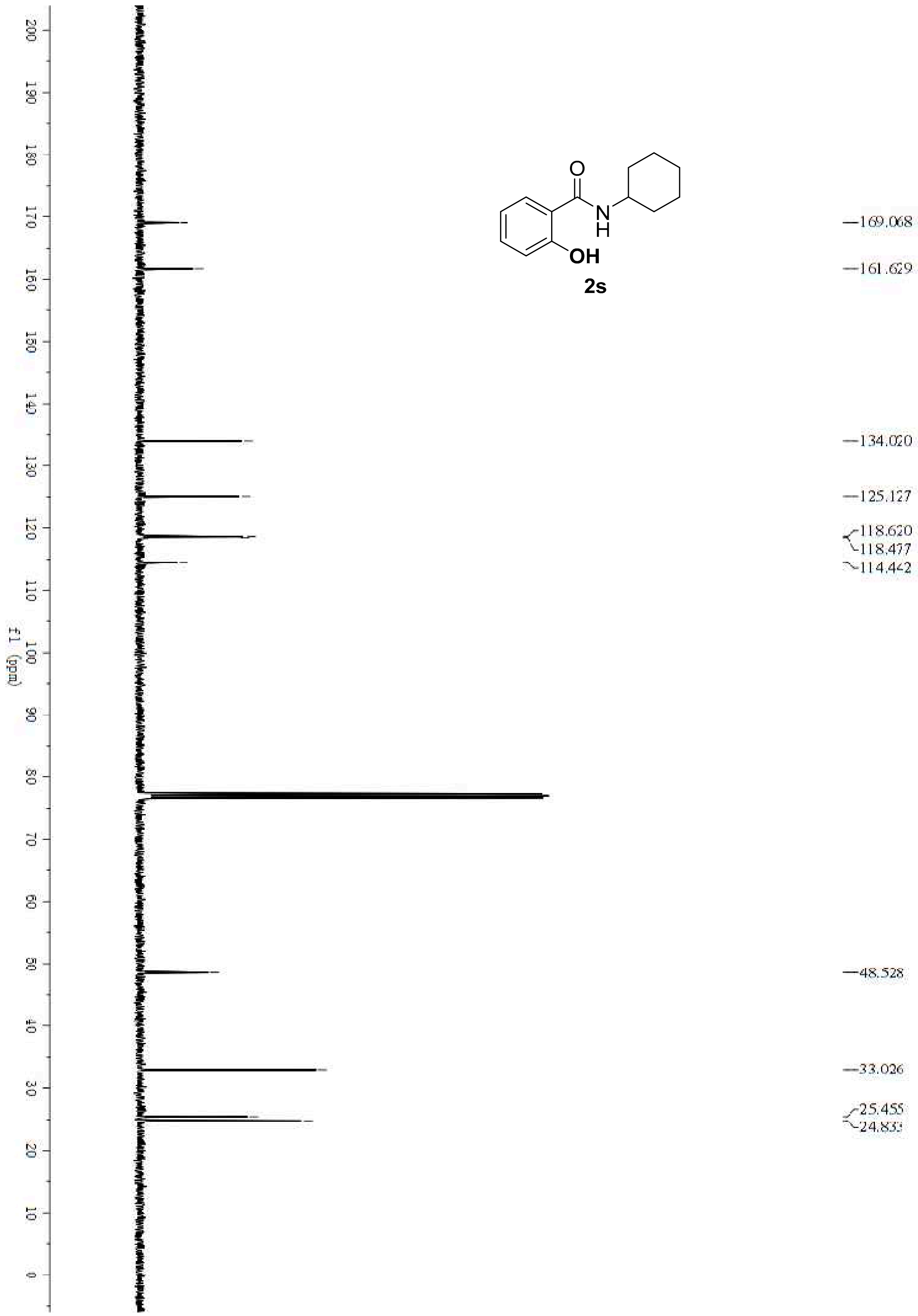

^{1}H NMR (400 MHz, $CDCl_3$) spectrum of **2t**

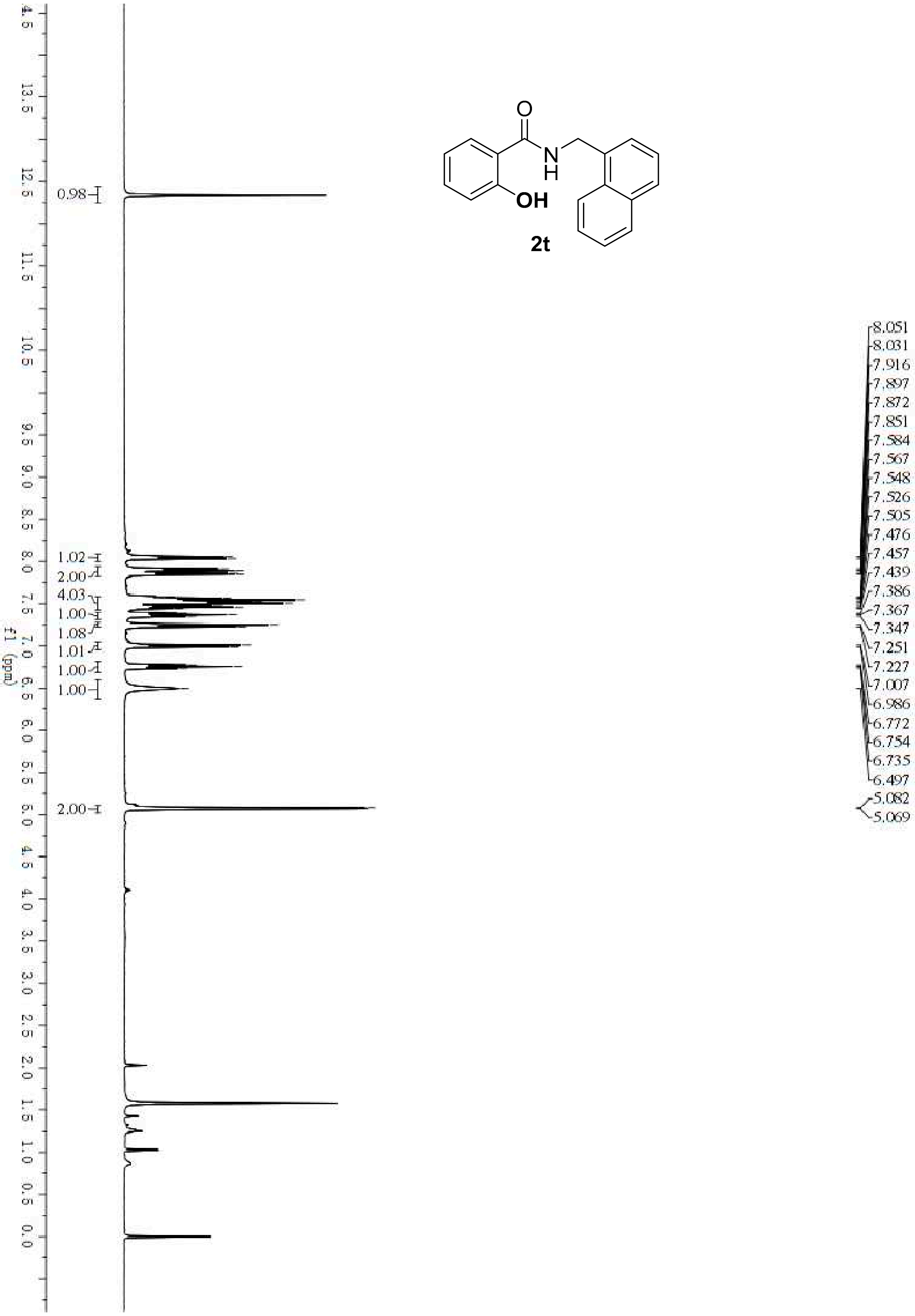

2t

^{13}C {^{1}H} NMR (100 MHz, $CDCl_3$) spectrum of **2t**

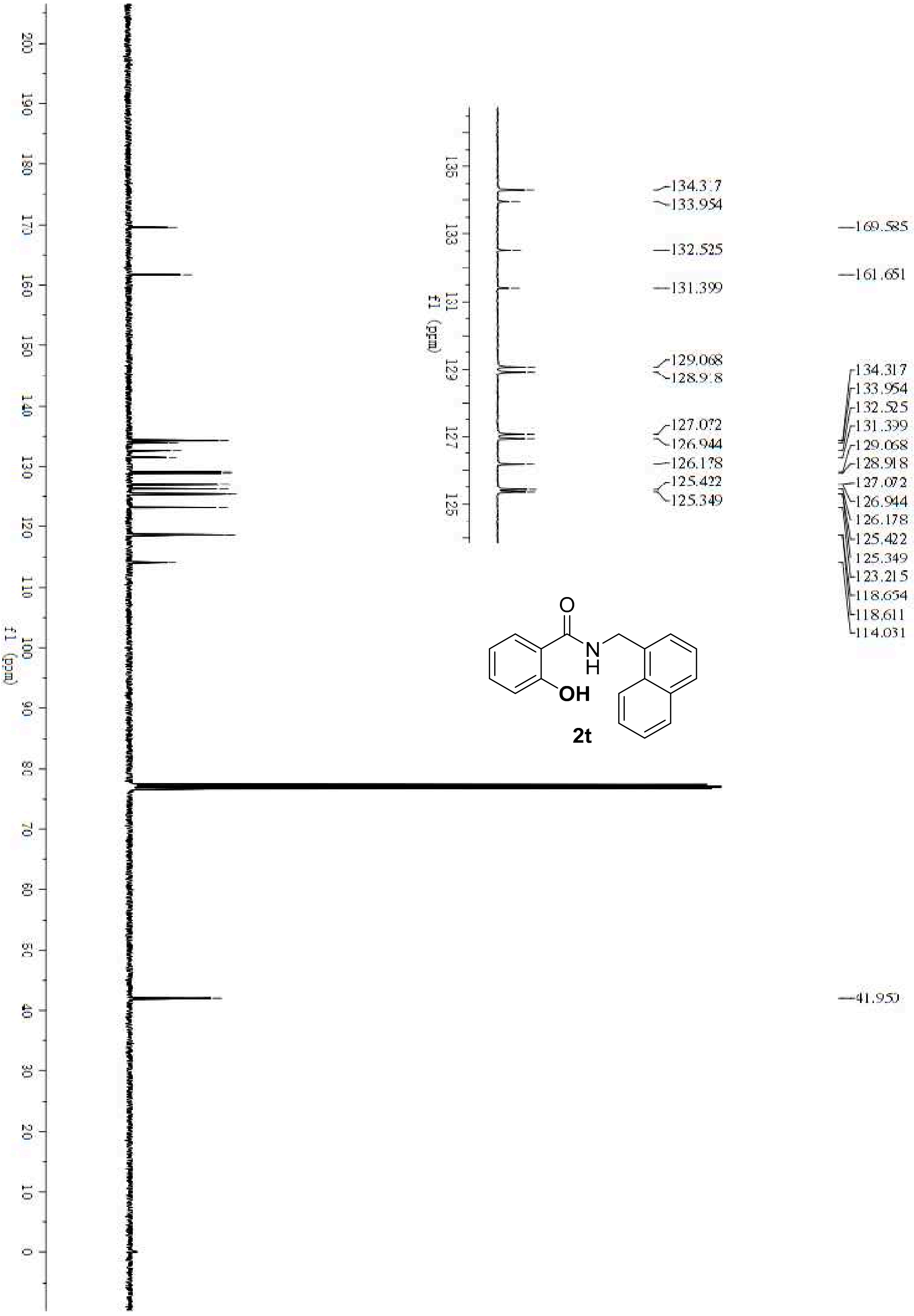

^{1}H NMR (400 MHz, $CDCl_3$) spectrum of **2u**

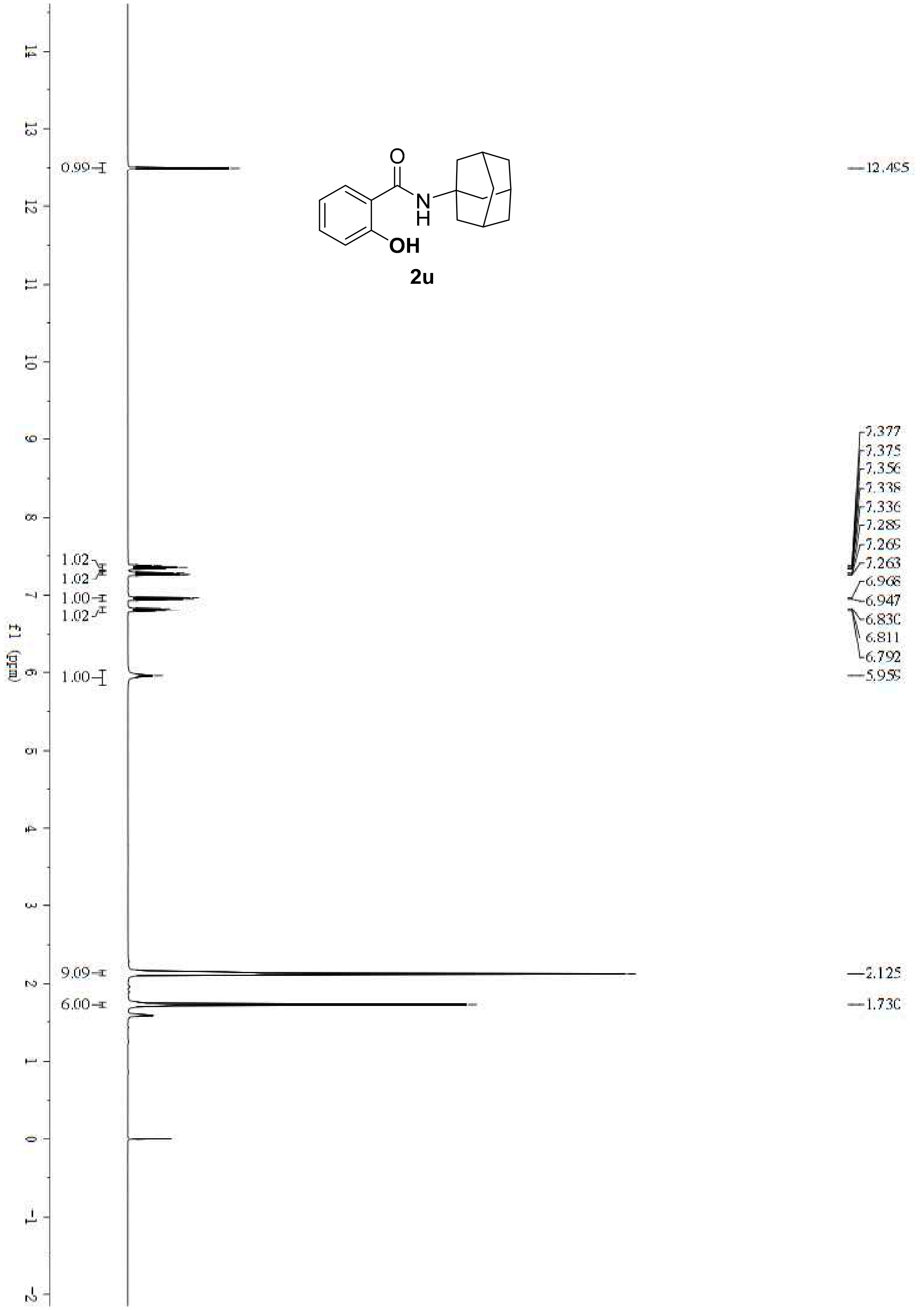

^{13}C {^{1}H} NMR (100 MHz, $CDCl_3$) spectrum of **2u**

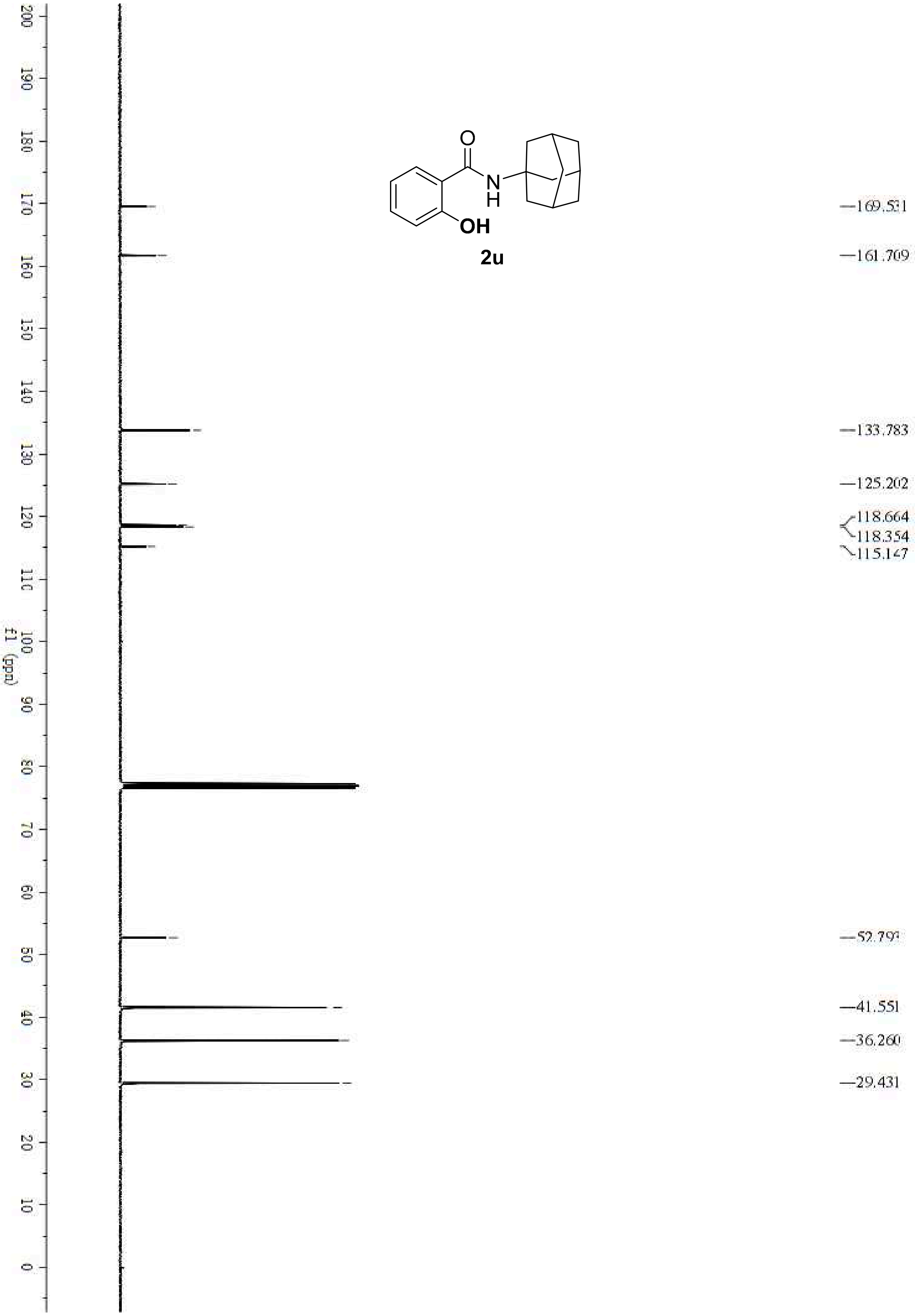

^{1}H NMR (400 MHz, $CDCl_3$) spectrum of **2v**

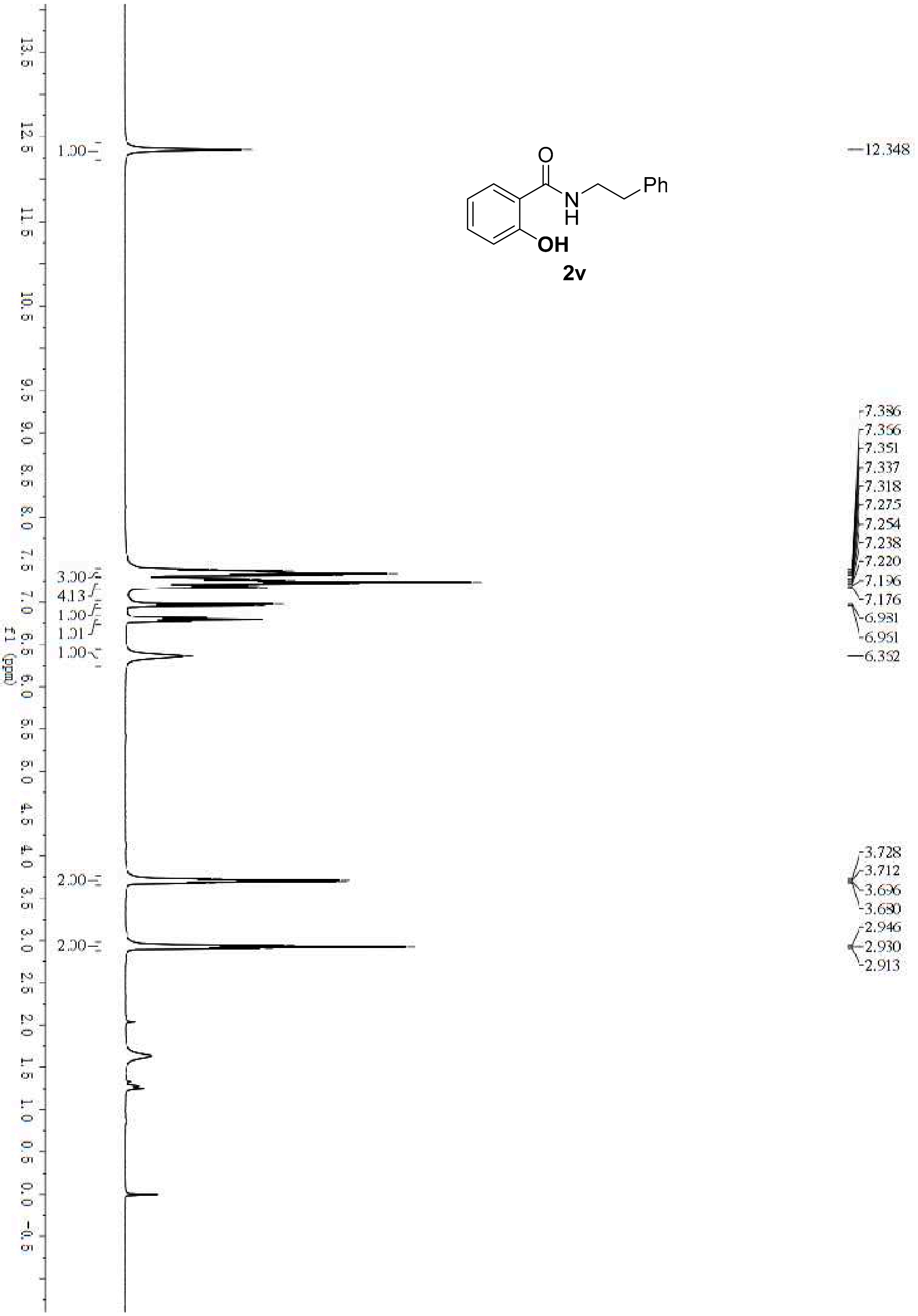

^{13}C {^{1}H} NMR (100 MHz, $CDCl_3$) spectrum of **2v**

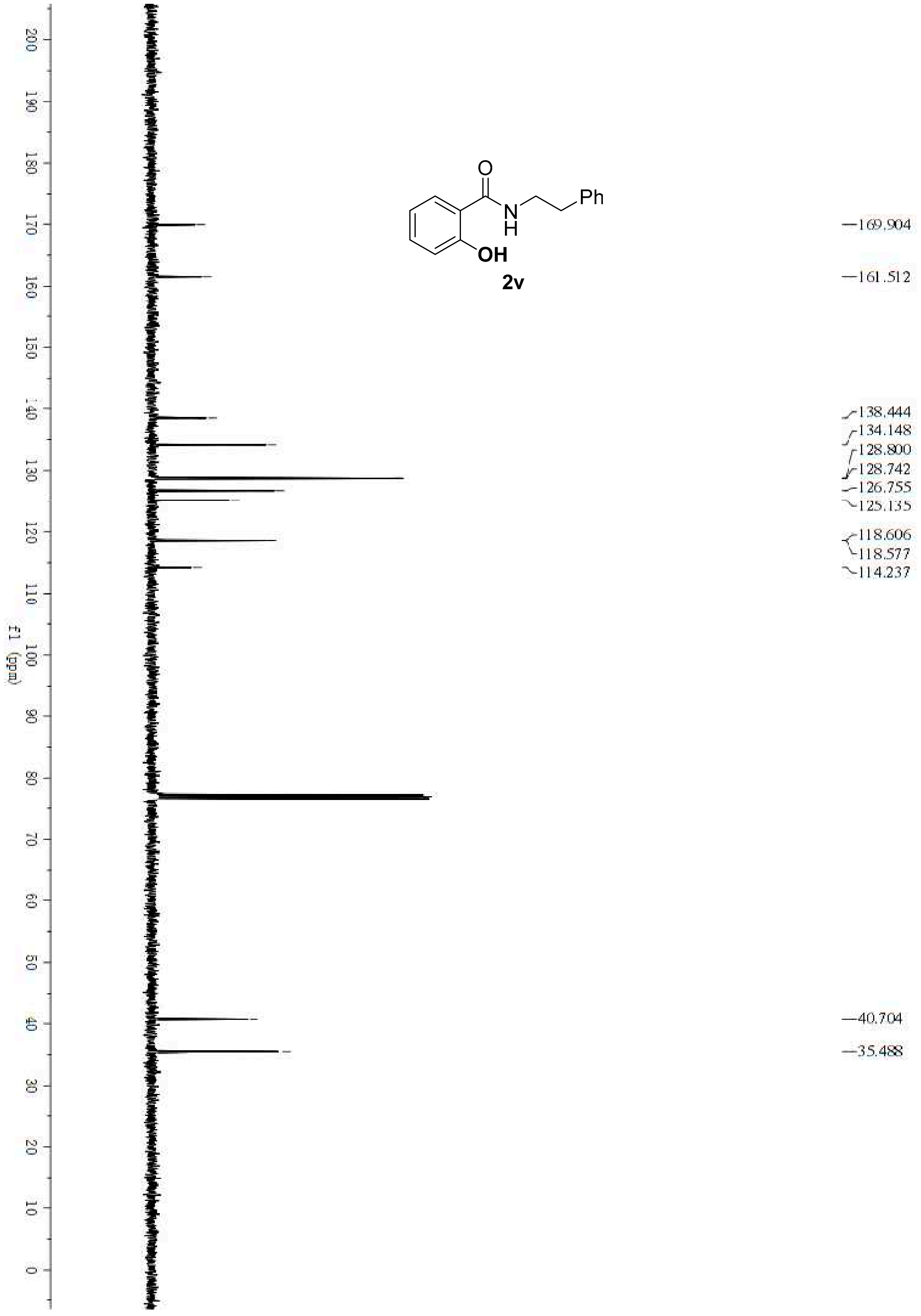

^{1}H NMR (400 MHz, $CDCl_3$) spectrum of **2w**

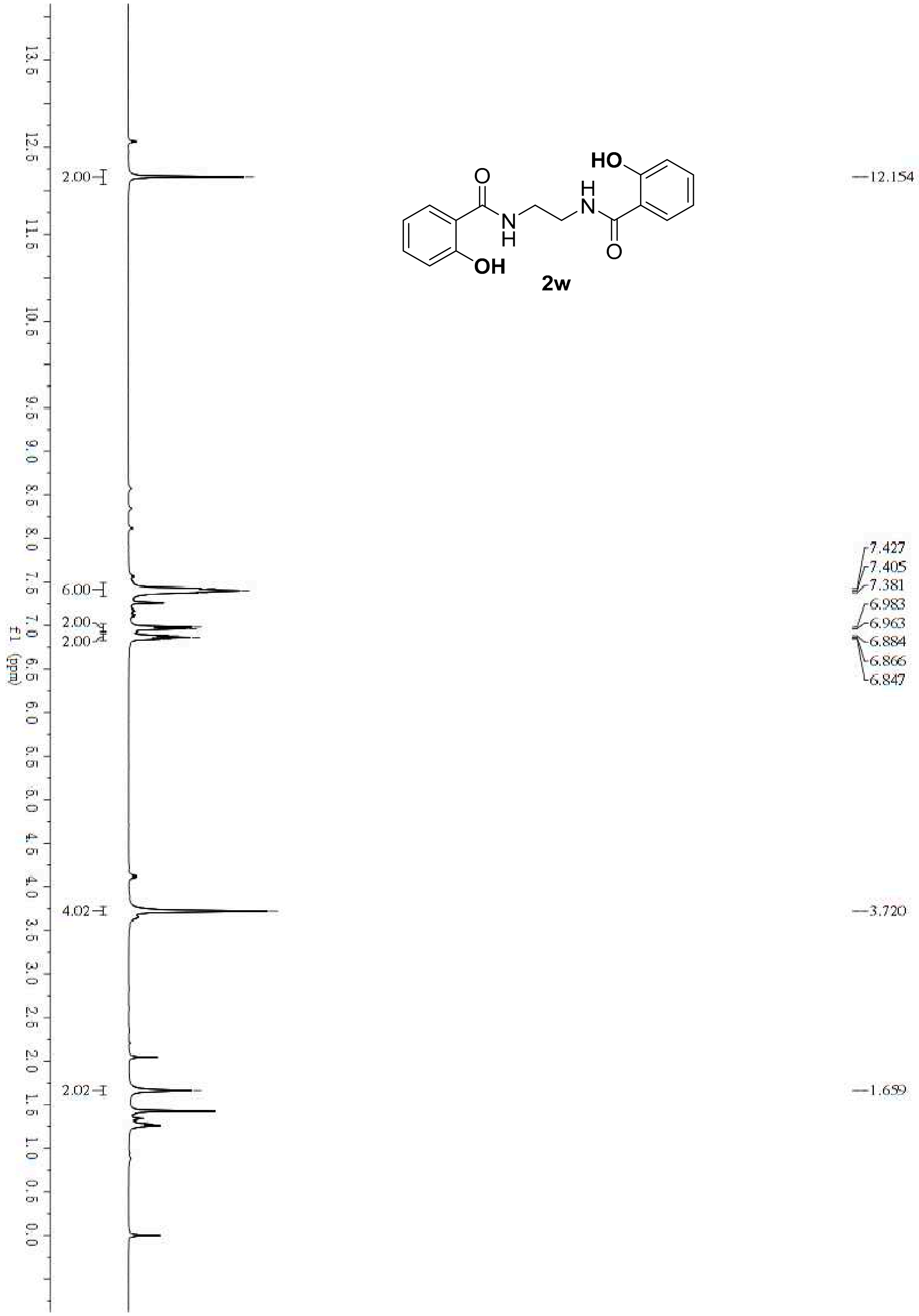

$^{13}C\ \{^{1}H\}$ NMR (100 MHz, $CDCl_3$) spectrum of **2w**

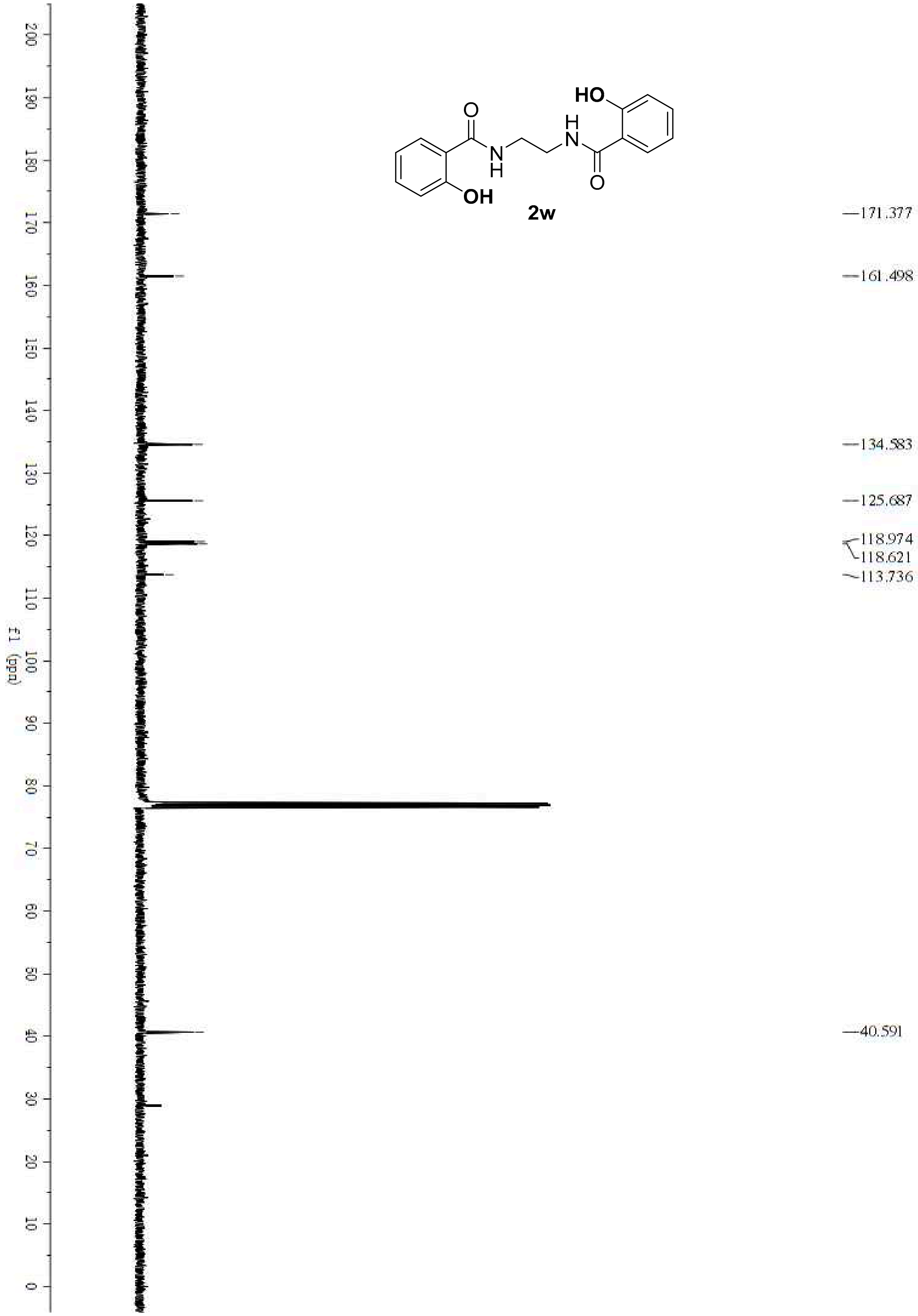

^{1}H NMR (400 MHz, DMSO-d$_6$) spectrum of **2x**

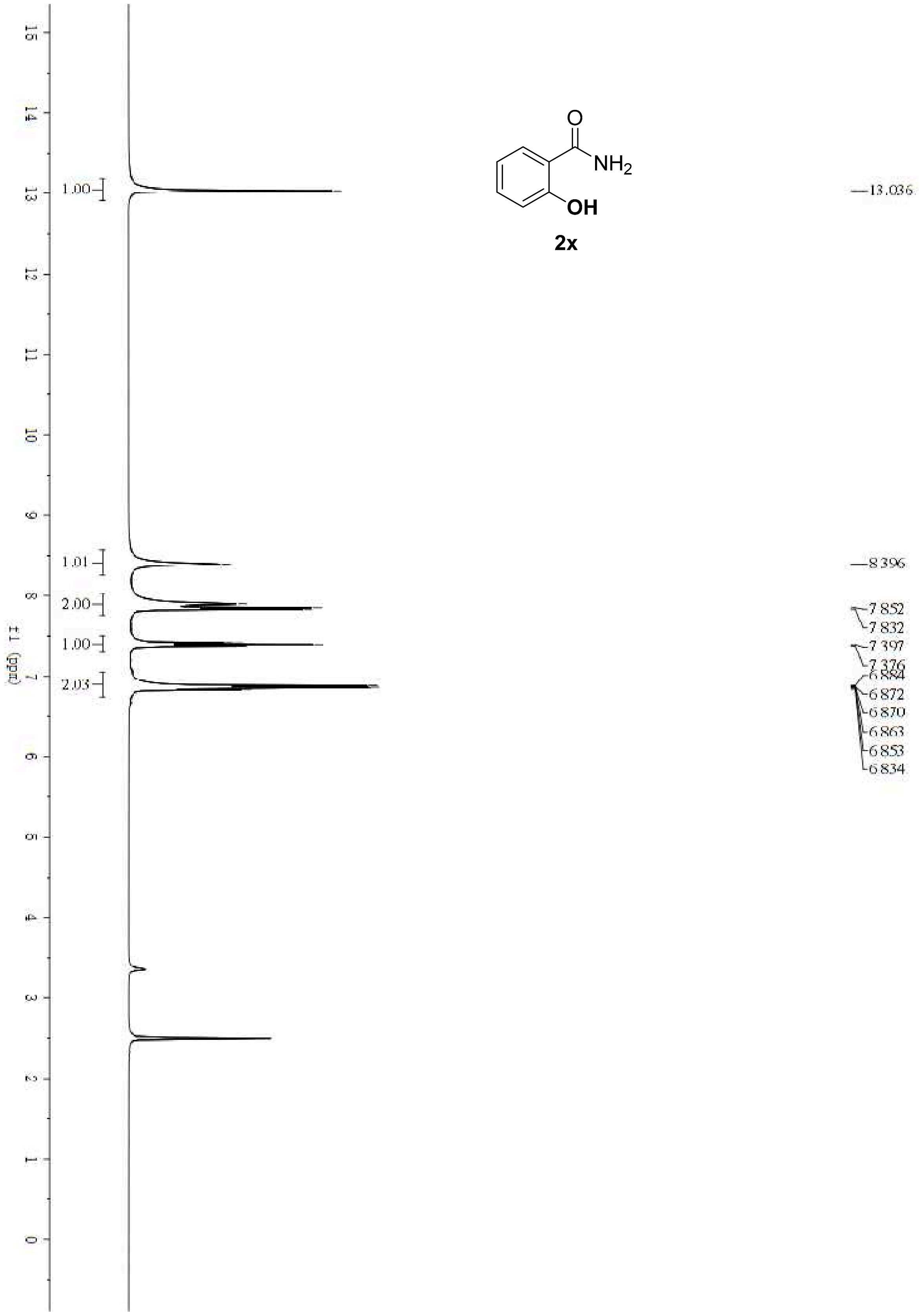

^{13}C {^{1}H} NMR (100 MHz, DMSO-d_6) spectrum of **2x**

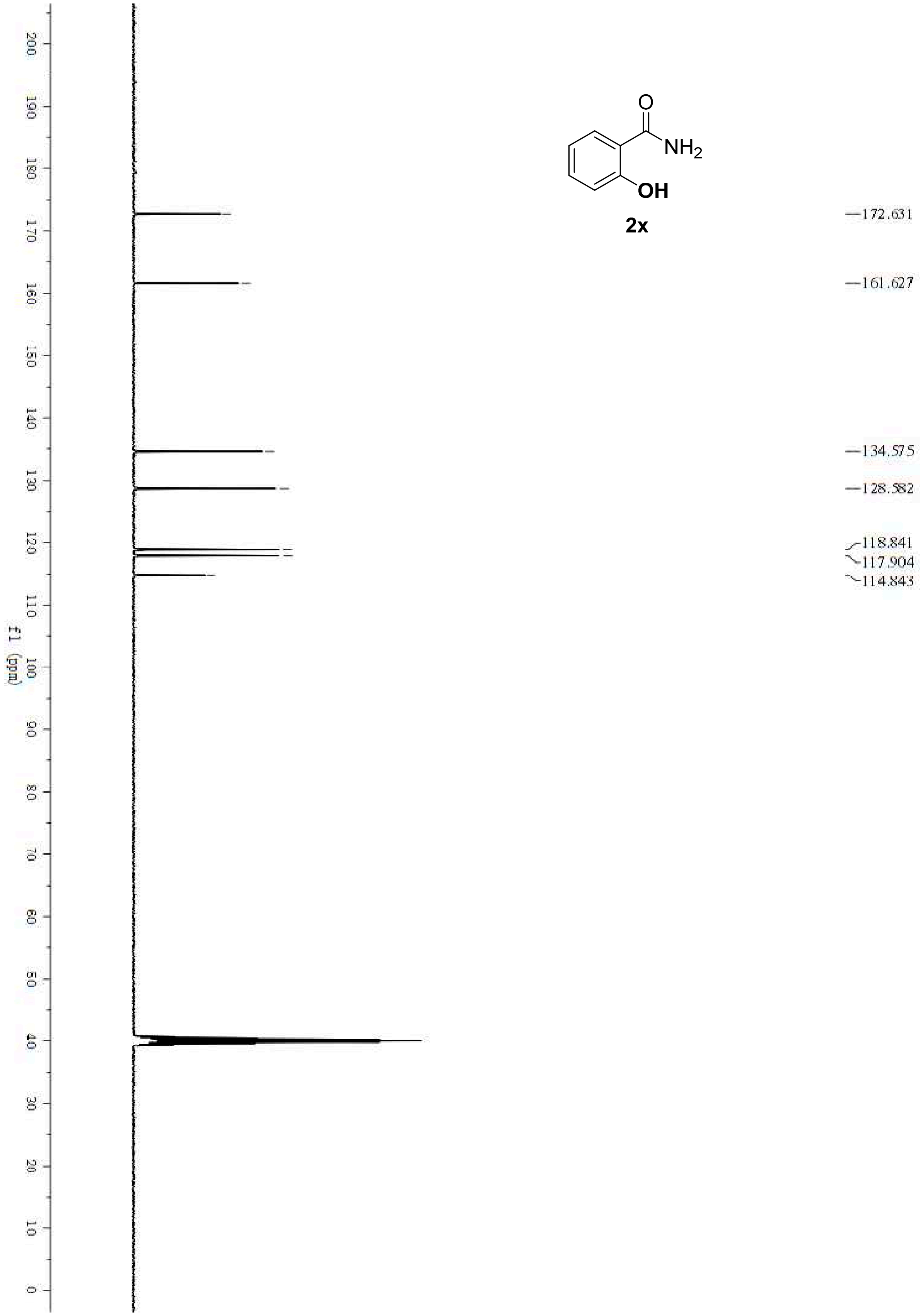

^{1}H NMR (400 MHz, $CDCl_3$) spectrum of **3a**

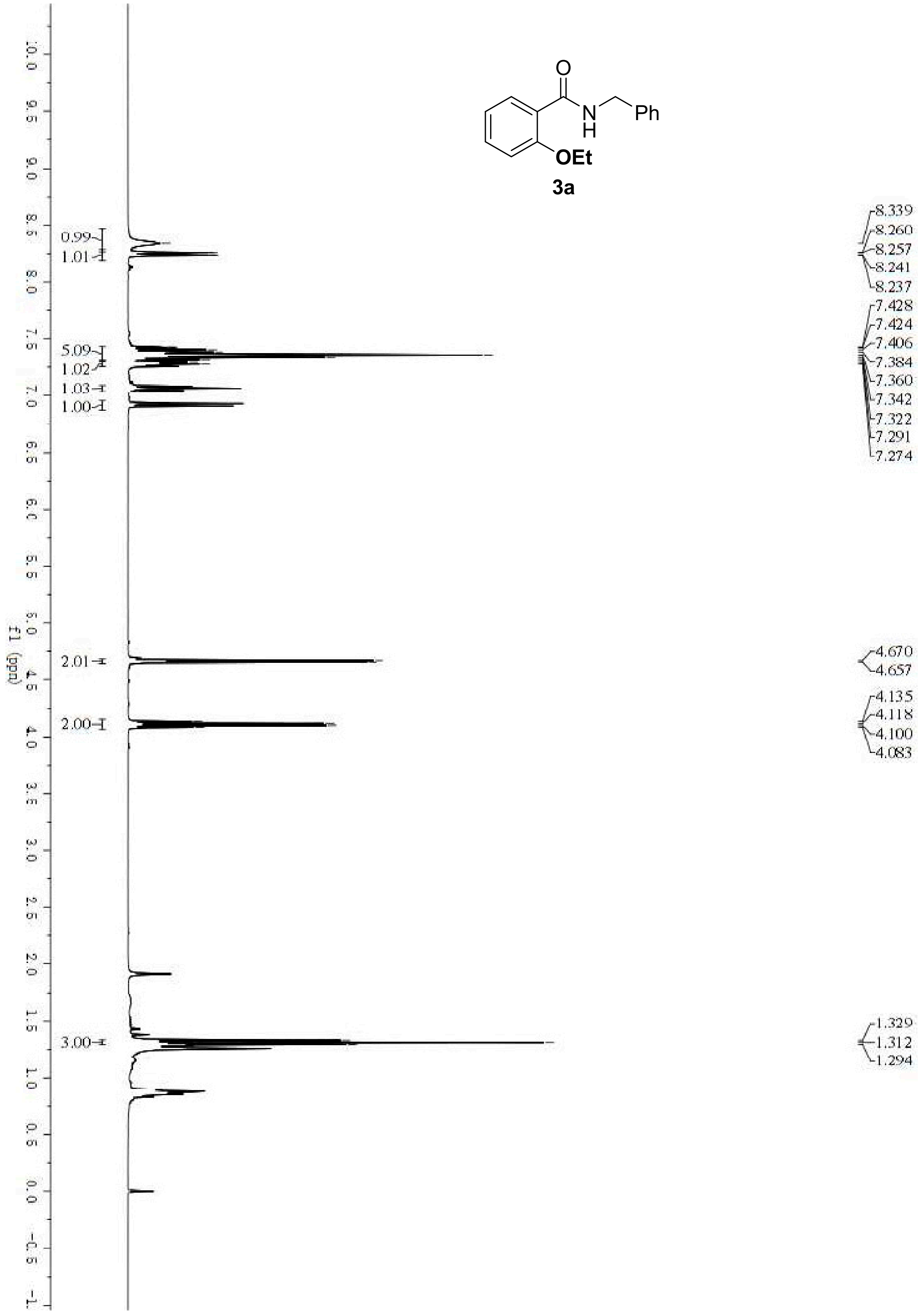

$^{13}C\ \{^1H\}$ NMR (100 MHz, $CDCl_3$) spectrum of **2a**

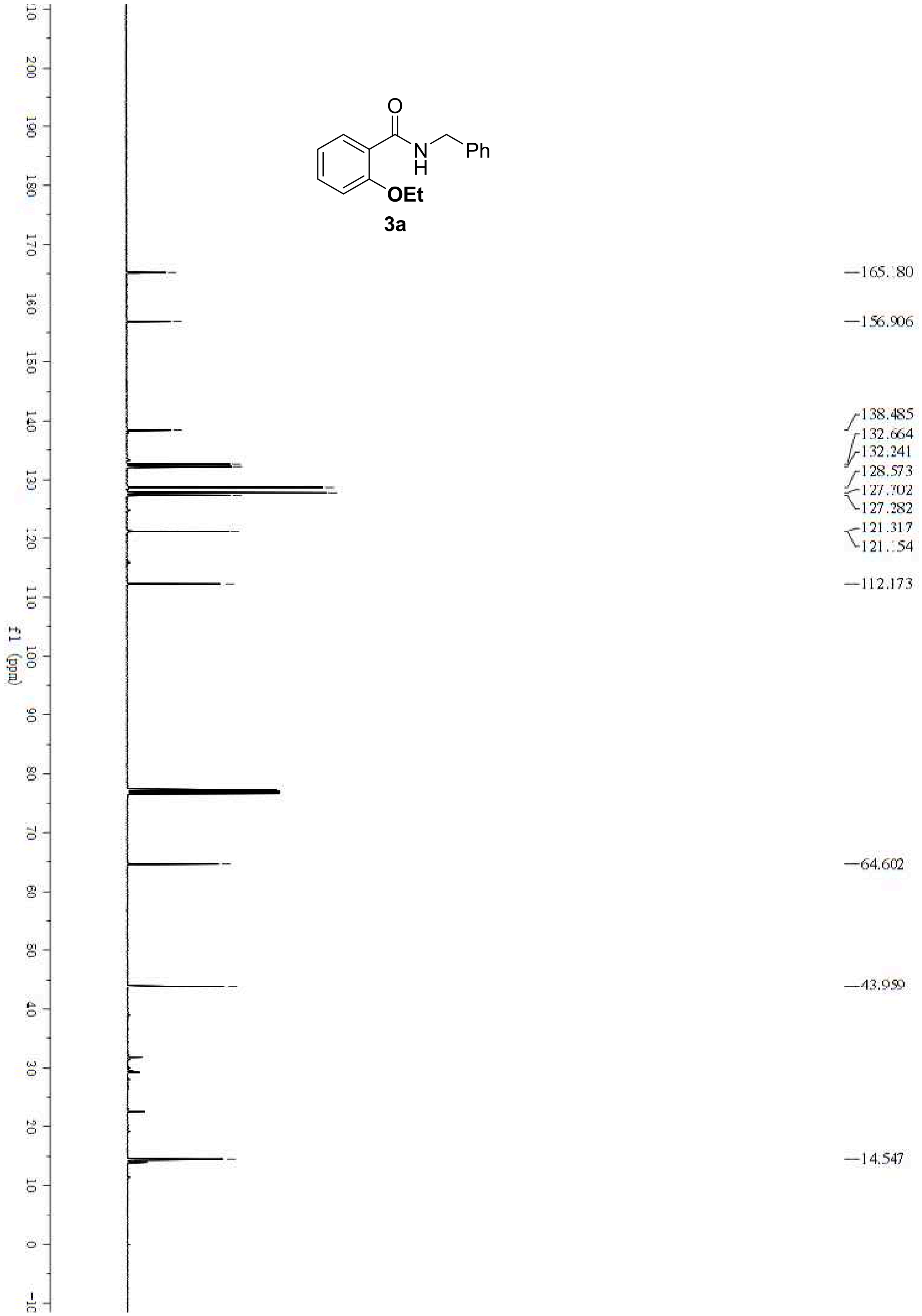

^{1}H NMR (400 MHz, $CDCl_3$) spectrum of **3b**

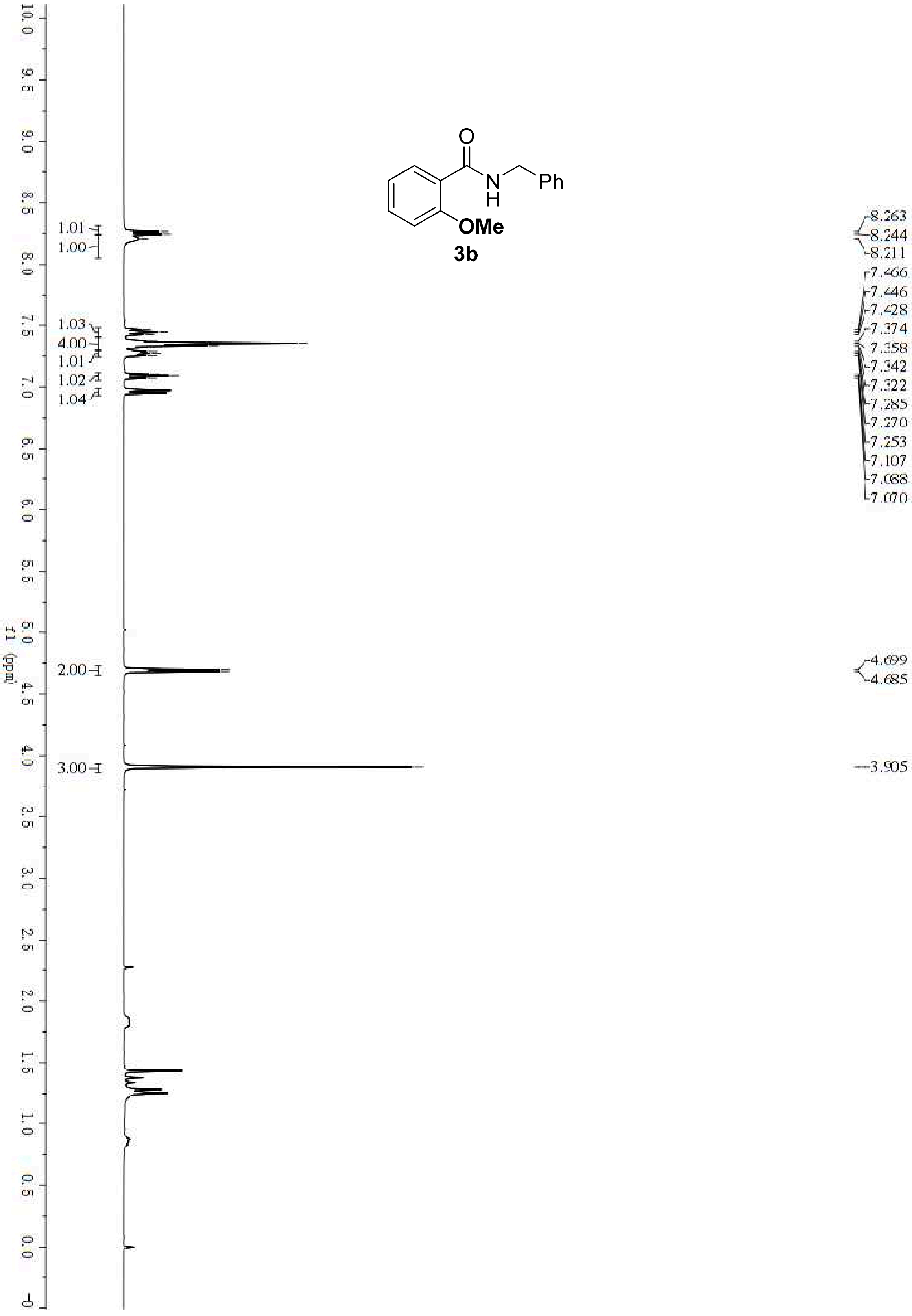

^{13}C {^{1}H} NMR (100 MHz, $CDCl_3$) spectrum of **3b**

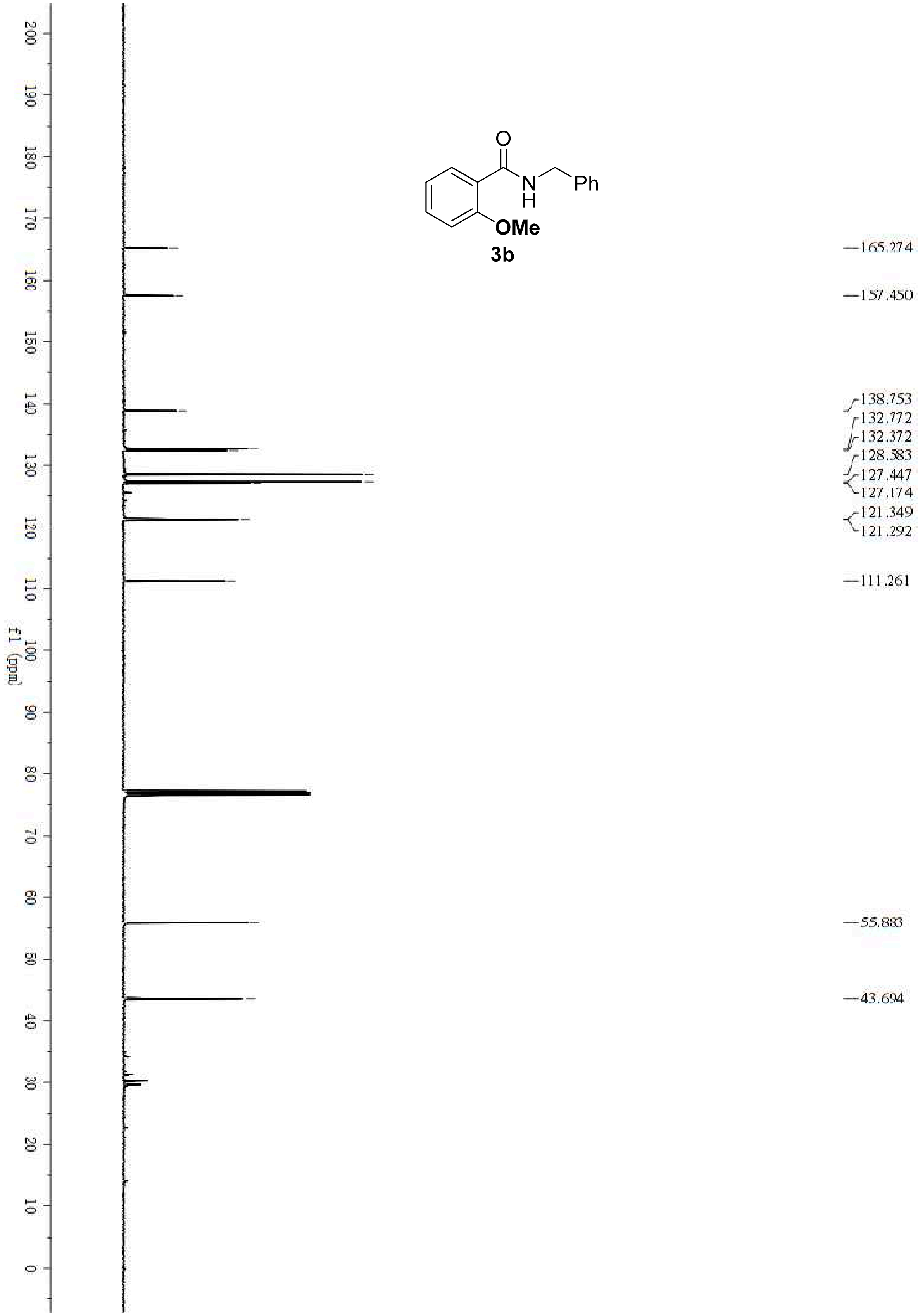

^{1}H NMR (400 MHz, $CDCl_3$) spectrum of **3c**

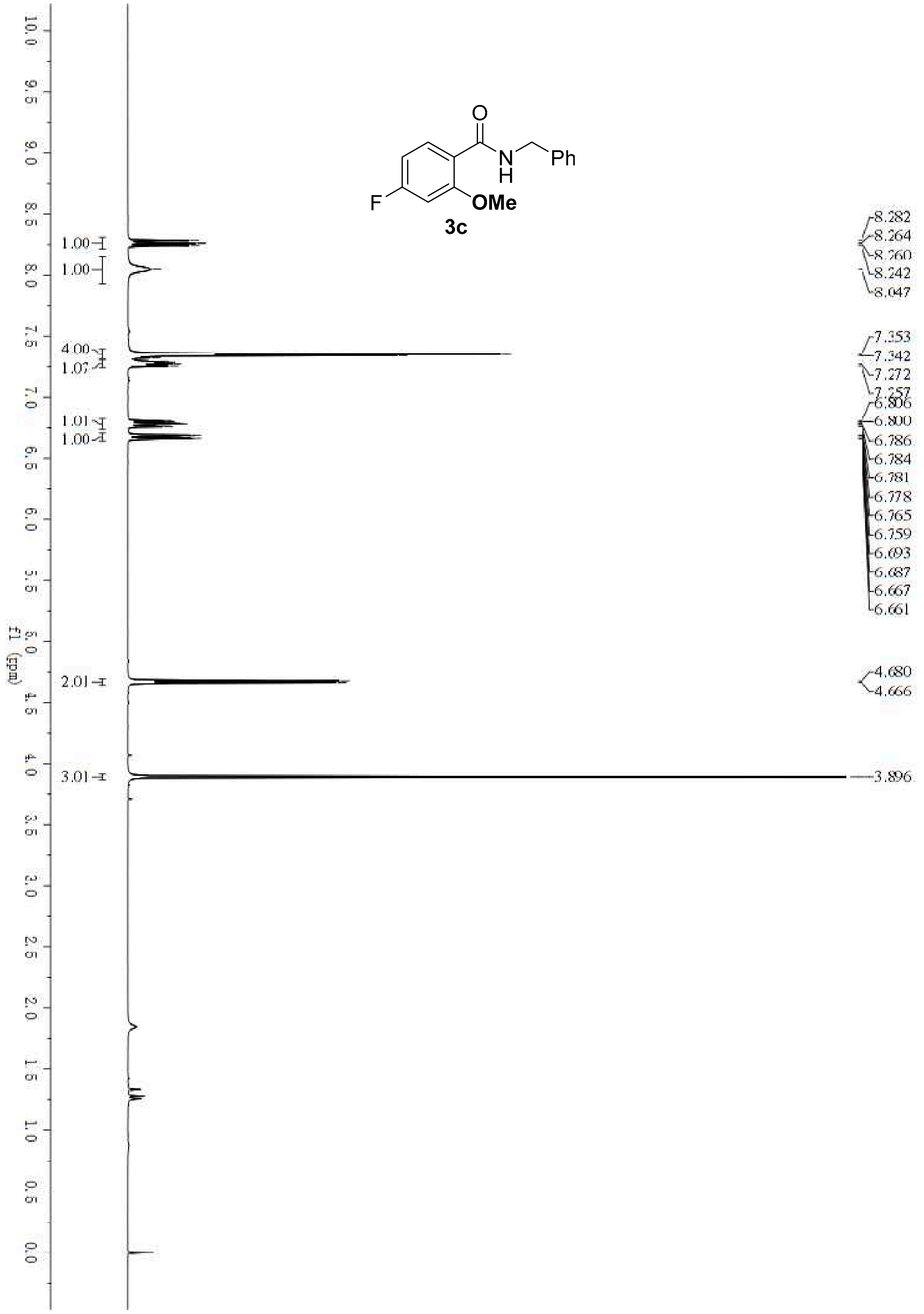

^{13}C {^{1}H} NMR (100 MHz, $CDCl_3$) spectrum of **3c**

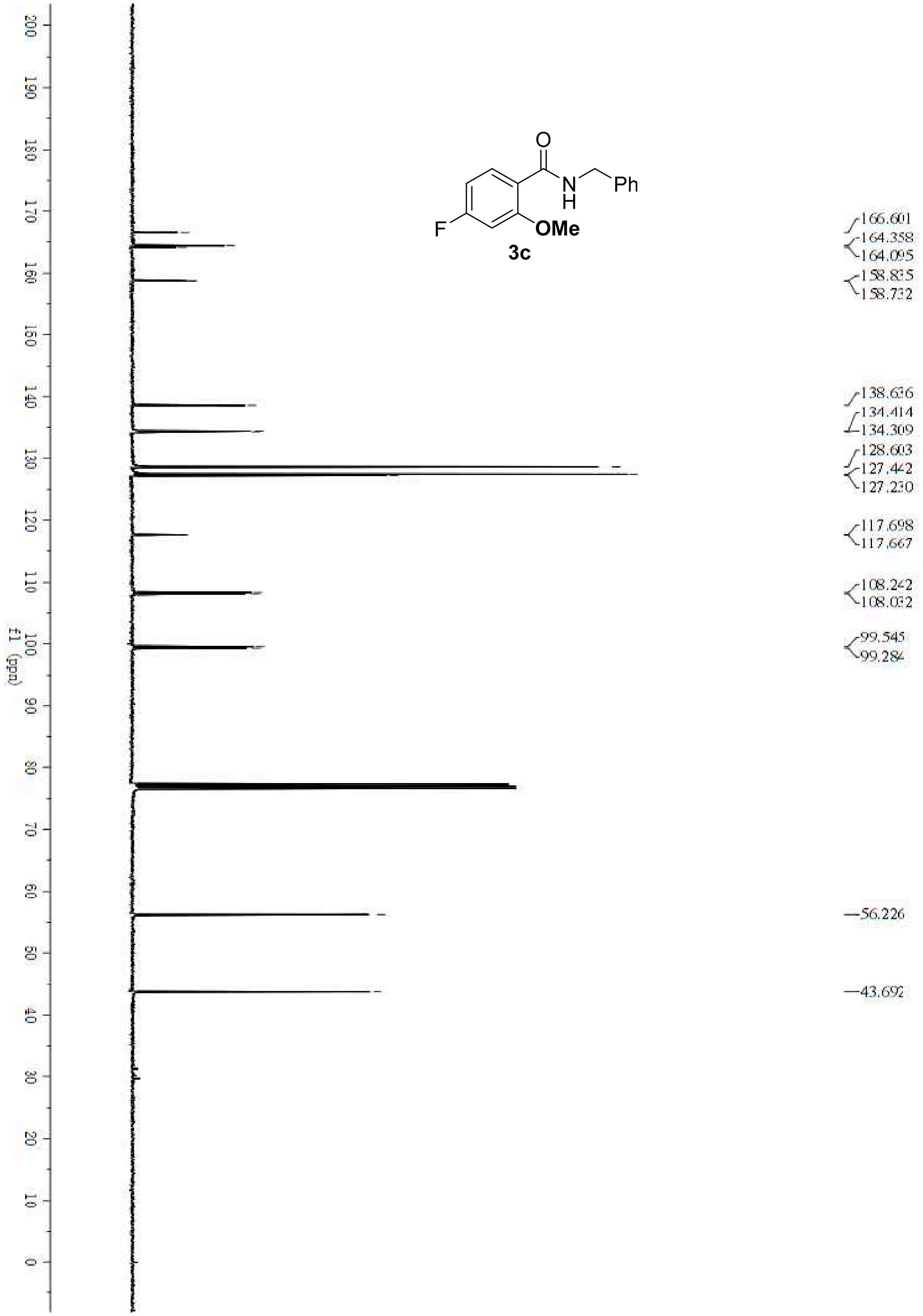

^{1}H NMR (400 MHz, DMSO-d$_6$) spectrum of **3d**

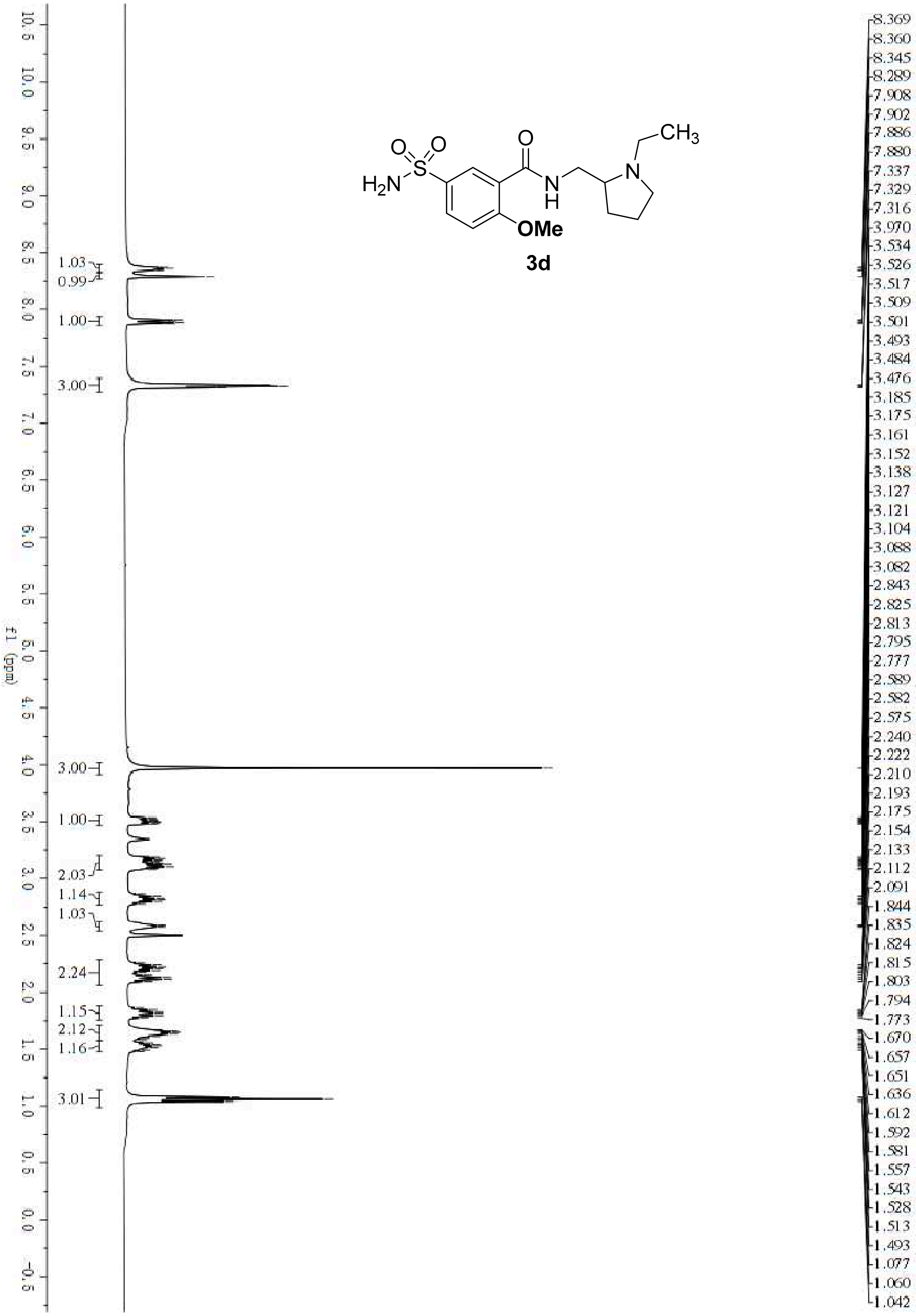

^{13}C {^{1}H} NMR (100 MHz, DMSO-d$_6$) spectrum of **3d**

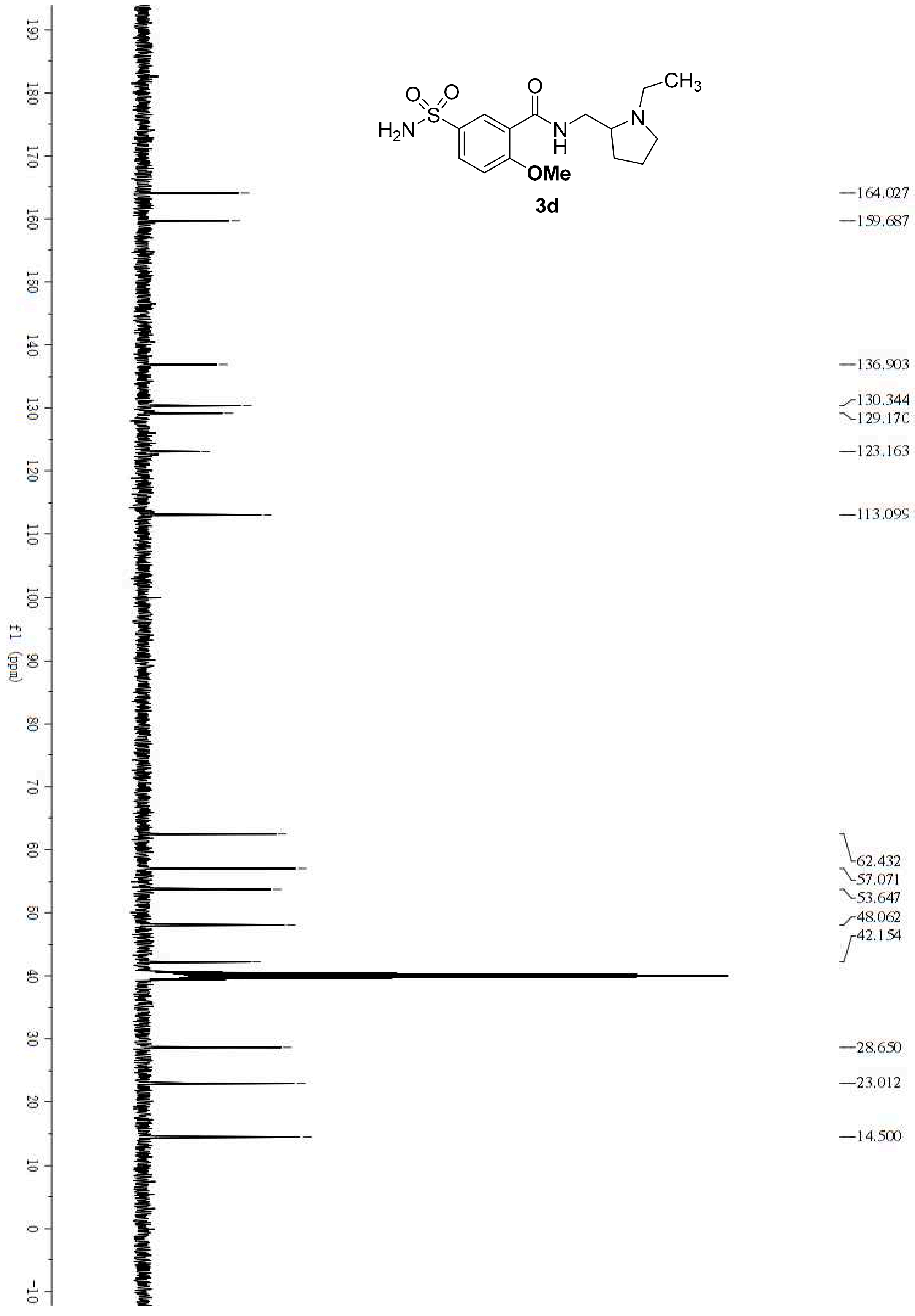

HRMS spectrum of **2e**

HRMS (ESI) m/z calcd for $C_{14}H_{13}BrNO_2$ (MH+) 306.0124, found 306.0119.

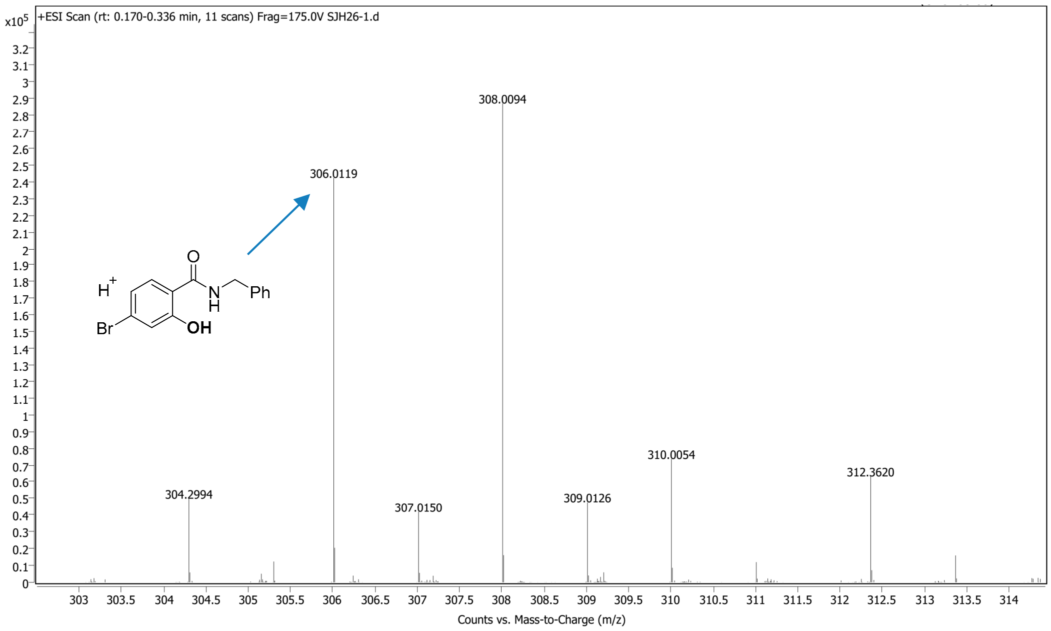

HRMS spectrum of **2f**

HRMS (ESI) m/z calcd for $C_{14}H_{13}FNO_2$ (MH+) 246.0925, found 246.0921.

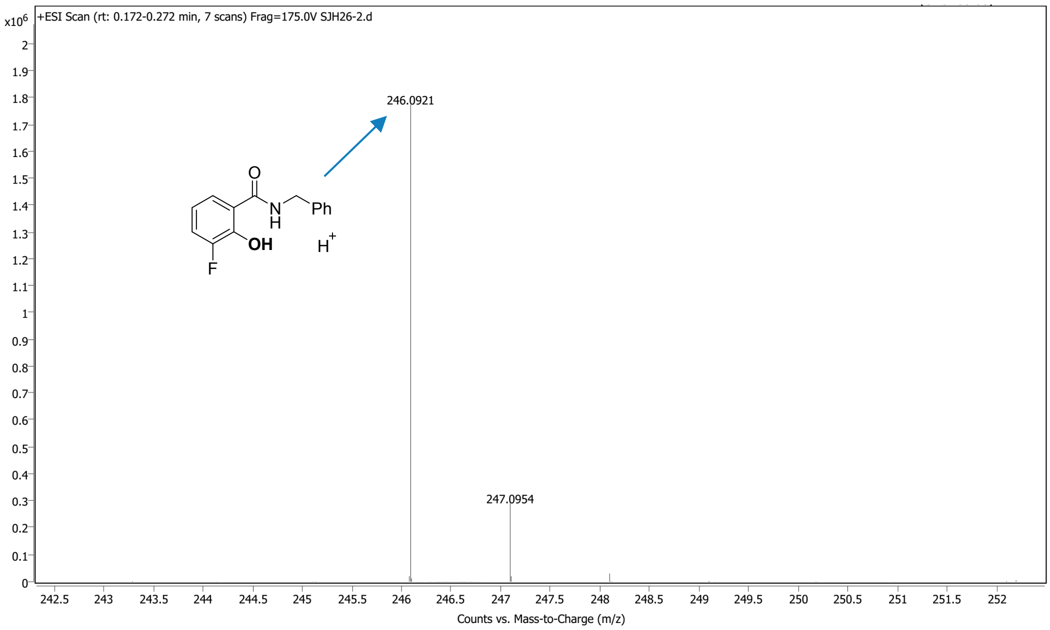

HRMS spectrum of **2h**

HRMS (ESI) m/z calcd for $C_{14}H_{13}FNO_2$ (MH+) 246.0925, found 246.0923.

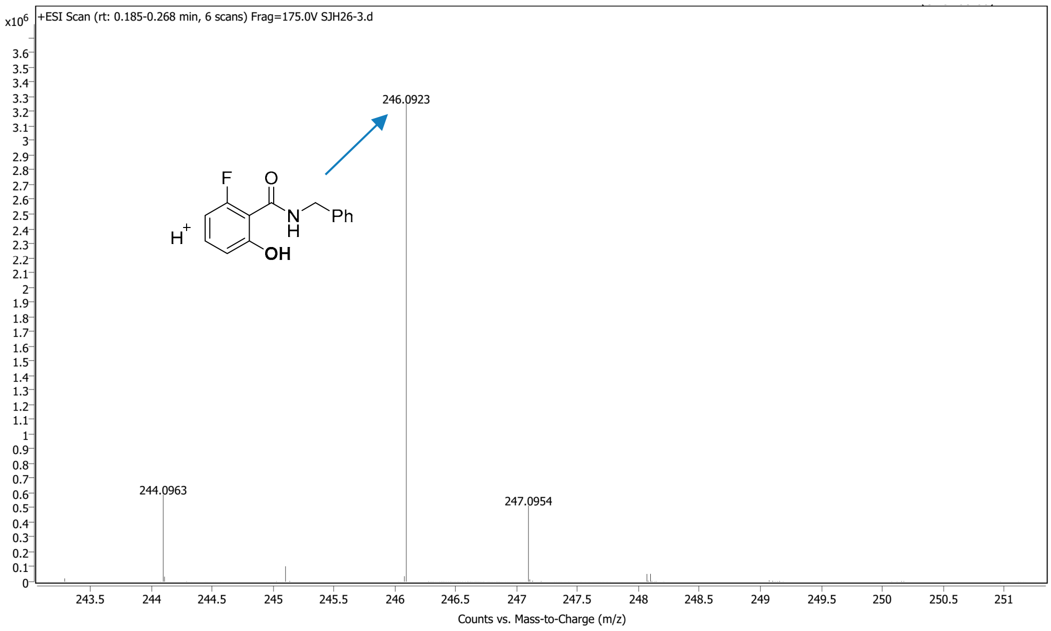

HRMS spectrum of **2t**

HRMS (ESI) m/z calcd for $C_{18}H_{16}NO_2$ (MH+) 278.1176, found 278.1170.

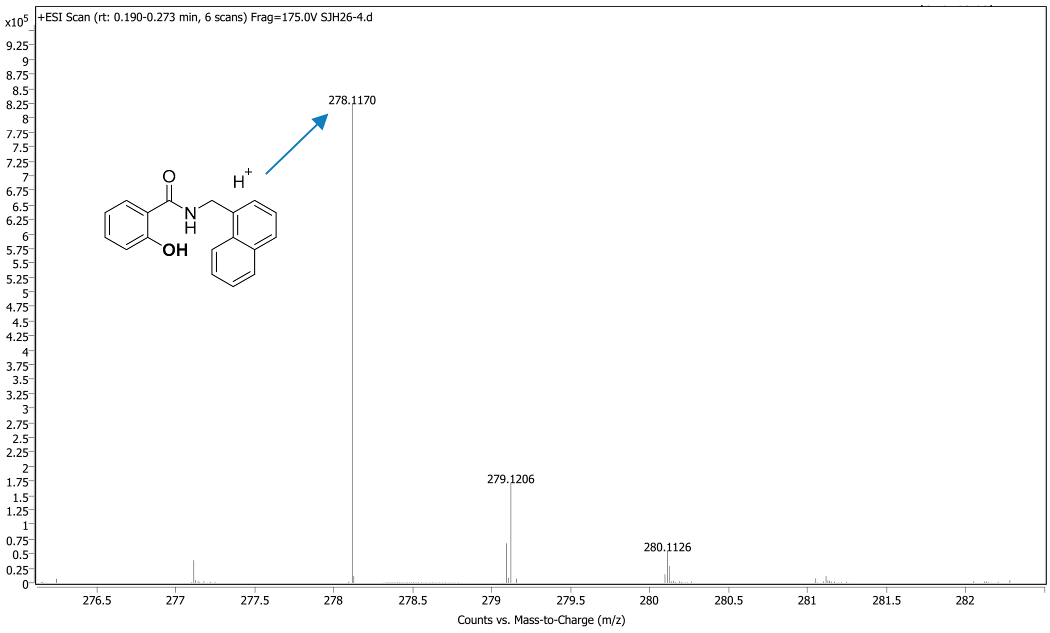

HRMS spectrum of **3c**

HRMS (ESI) m/z calcd for $C_{15}H_{15}FNO_2$ (MH+) 260.1081, found 260.1079.

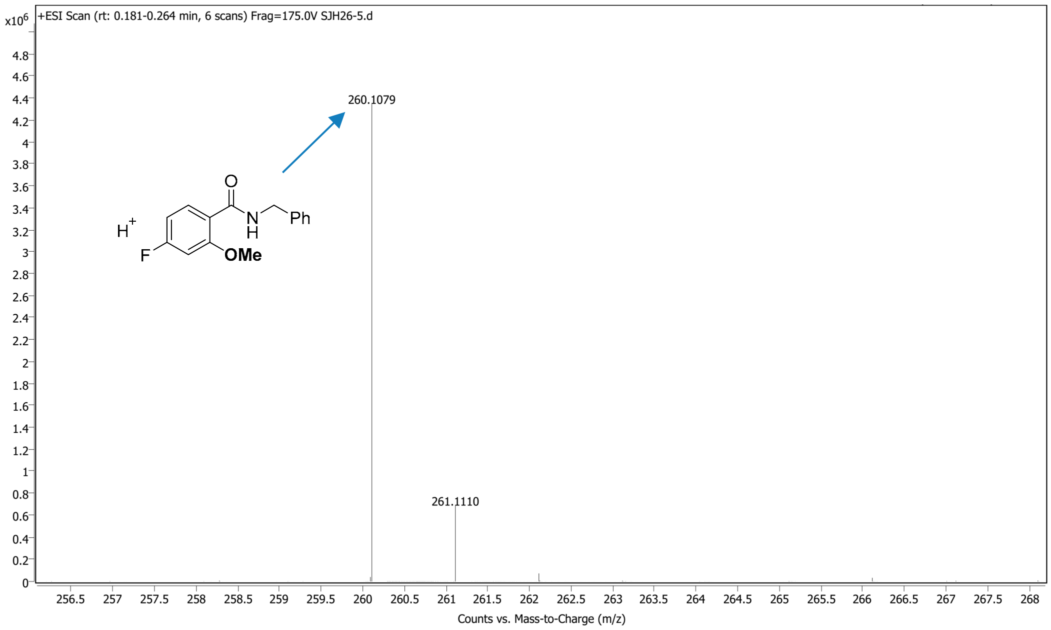

Supplement: Supplementary file 1 [file molecules-31-01887-s001.zip › molecules-4320177-supplementary.pdf]
